# Structural characterization of zebrafish Ngly2, an ovary-enriched acid PNGase required for egg-free glycan production

**DOI:** 10.1016/j.jbc.2025.110906

**Published:** 2025-11-05

**Authors:** Akinobu Honda, Katsuhiko Kamada, Junichi Seino, Hiroto Hirayama, Haruhiko Fujihira, Masashi Ueki, Toshiyuki Shiraki, Raymond N. Burton-Smith, Kazuyoshi Murata, Nozomi Ishii, Ichiro Matsuo, Tadashi Suzuki

**Affiliations:** 1 Glycometabolic Biochemistry Laboratory, RIKEN Pioneering Research Institute, RIKEN, Wako, Saitama, Japan; 2 Institute for Glyco-Core Research (iGCORE), Gifu University, Gifu, Japan; 3 Division of glycobiologics, Juntendo University Graduate School of Medicine, Tokyo, Japan; 4 Research Resources Division, RIKEN Center for Brain Science, RIKEN, Wako, Saitama, Japan; 5 Exploratory Research Center on Life and Living Systems (ExCELLS), and National Institute for Physiological Sciences (NIPS), National Institutes of Natural Sciences, Okazaki, Aichi, Japan; 6 Department of Physiological Sciences, School of Life Science, The Graduate University for Advanced Studies (SOKENDAI), Okazaki, Aichi, Japan; 7 Graduate School of Science and Technology, Gunma University, Kiryu, Gunma, Japan

**Keywords:** acid PNGase, *N*-glycanase, free oligosaccharide, zebrafish

## Abstract

Peptide:*N*-glycanase (PNGase) is a deglycosylating enzyme acting on asparagine(*N*)-linked glycans on glycoproteins. It is well established that fish possesses two PNGases with distinct properties. One is a cytosolic PNGase (NGLY1 in humans), active at neutral pH and widely conserved among eukaryotes. The other is called acid PNGase and is found in fish embryos; it is active at acidic pH and is believed to be of lysosomal origin. The gene encoding the acid PNGase has not been identified in animals, and its evolutionary distribution has remained unknown. In this study, we identified the gene encoding the acid PNGase, which we named Ngly2, in zebrafish (*Danio rerio*). Interestingly, zebrafish Ngly2 was found to have structural similarity with bacterial PNGase (PNGase F) and indeed appeared to share common catalytic residues, despite the fact that these two enzymes exhibit quite distinct pH profiles. The structure of zebrafish Ngly2 was determined by cryo-EM, showing that it forms homodimers and that its substrate is accommodated in the cleft between the protease-associated domain and PNGase domain, where the catalytic residues are located. Tissue distribution analysis indicated that *ngly2* was almost exclusively expressed in the ovary. A zebrafish *ngly2*-KO line was found to be fertile, survive well, and show no overt phenotypes, although it had significantly smaller fertilized eggs. It was also revealed that *ngly2* KO resulted in a substantial reduction in the level of free oligosaccharides in fertilized eggs, implying that Ngly2, not Ngly1, is responsible for the formation of most, if not all, egg-free glycans.

Glycans, along with nucleic acids, lipids, and proteins, are among the important biomacromolecules that make up our bodies. Glycans can be attached to polypeptides by a process known as glycosylation. The glycan structures attached to proteins can be highly complex, with numerous possibilities for branching in addition to two distinct anomeric linkages, resulting in much greater structural diversity than seen in linear nucleic acid or polypeptide structures (1). Among the different types of protein glycosylation, *N*-glycosylation is the most studied form in eukaryotes, and it has been estimated that more than half of human proteins are *N*-glycosylated (2). Glycosylation has been shown to play an important role in many biological processes, including intracellular–intercellular localization and quality control of proteins, cell–cell and cell–matrix interactions, cellular differentiation, fertilization, and the immune response (3).

Free *N*-glycans (FNGs), that is, free oligosaccharides that are structurally related to *N*-linked glycans, are found widely from bacteria to mammals (4). While the accumulation of complex-type, sialylated FNGs has been observed under various disease conditions, such as lysosomal storage diseases (5, 6, 7), autophagy-defective cells (8), and various cancer tissues/cells (9, 10), FNGs in normal tissues were also described in unfertilized eggs of freshwater trout (ayu; *Plecoglossus altivelis*) (11). Subsequently, FNGs were found in oocytes and embryos from not only various fish species, such as dace (*Tribolodon hakonensis*) (12), flounder (*Paralichthys olivaceus*) (13), medaka (*Oryzias latipes*) (14, 15), small-spotted catshark (*Scyliorhinus canicula*) (16) and zebrafish (*Danio rerio*) (17), but also in hen eggs (18). In most cases, these FNGs are predominantly of the Gn2 type, retaining the di-*N*-acetylchitobiose structure (GlcNAcβ1–4GlcNAc) at their reducing termini, whereas a minor portion of FNGs can occur as the Gn1 type, bearing only a single GlcNAc at their reducing termini (12, 17).

Peptide:*N*-glycanase (PNGase; Enzyme Commission number: 3.5.1.52) is a deglycosylating enzyme acting on *N*-glycans. PNGases from bacteria (called PNGase F) (19) and plants (called PNGase A) (20), both commercially available, have been extensively used as a tool reagent to analyze the structure–functions of *N*-glycans. By contrast, PNGases of animal origin were not identified until 1991, when PNGase activity was detected in medaka fish eggs and embryos (21). After this discovery, PNGase activity was also detected in mammalian cultured cells (22, 23), mouse organs (24), and hen oviduct (25). Among these, fish embryo enzymes were quite distinct from the others because they exhibit optimal activities at acidic pH (3.5–4.5) and were thus assumed to be of lysosomal origin (26), whereas the others show enzyme activities at neutral pH (22, 24, 25, 27). Later, the gene encoding the neutral PNGase was identified in budding yeast (*PNG1*), and a genome survey revealed that this gene is conserved among eukaryotes, including mammals (the human gene is now called *NGLY1*) (28). Regarding the physiological functions of Ngly1, it has been demonstrated that the enzyme is involved in the degradation of misfolded *N*-glycoproteins in the cytosol (29, 30, 31). Aside from this role, Ngly1 can play a role in “amino acid editing” by converting glycosylated Asn (N) to Asp (D) residues (23). This protein editing has been shown to be critical for the transcriptional activation of proteasome subunits in *Caenorhabditis elegans* (32). In 2012, a human hereditary disorder caused by mutations of the *NGLY1* gene was identified through an exome analysis, clearly demonstrating the functional importance of NGLY1 in human development (33). Symptoms of NGLY1 deficiency include global developmental delay, movement disorder, hypotonia, abnormalities in electroencephalogram, and the absence of tears (34, 35). Since the discovery of NGLY1 deficiency, Ngly1 research has accelerated around the world with the aim of elucidating the detailed molecular mechanism responsible for its pathogenesis as well as establishing therapeutic options for this genetic disorder.

In sharp contrast to the case with Ngly1, the gene encoding the acid PNGase in fish (26), the very first animal-origin PNGase reported, remained unknown even to this day. However, as mentioned above, FNGs are accumulated in the ovary and embryos of various fish species, suggesting that the de-*N*-glycosylation is a highly important biological process in fish. These FNGs can be generated from hyosophorin (13, 14), a polyprotein in cortical alveoli that can be proteolyzed upon cortical reaction, and from glycophosphoprotein (also known as phosvitin) (15), derived from vitellogenin, a liver-derived egg-yolk protein. Given that free glycans are generated from proteins presumably involved in fertilization or embryonic development, de-*N*-glycosylation by PNGases is expected to play a role in these processes.

In this study, we successfully identified the gene encoding the acid PNGase. The enzymatic properties of the enzyme, which we named Ngly2 after “*N*-glycanase 2,” were extensively characterized. Furthermore, we analyzed the structure of Ngly2 using cryo-EM, showing that the enzyme forms a homodimer and has catalytic residues located within a groove, implying limited substrate accessibility to this enzyme. We also generated *ngly2*-KO zebrafish and revealed that FNGs in fertilized eggs are predominantly generated by Ngly2.

## Results

### Identification of acid PNGase with the same domain as PNGase F in zebrafish (*D. rerio*)

**Figure 1:**
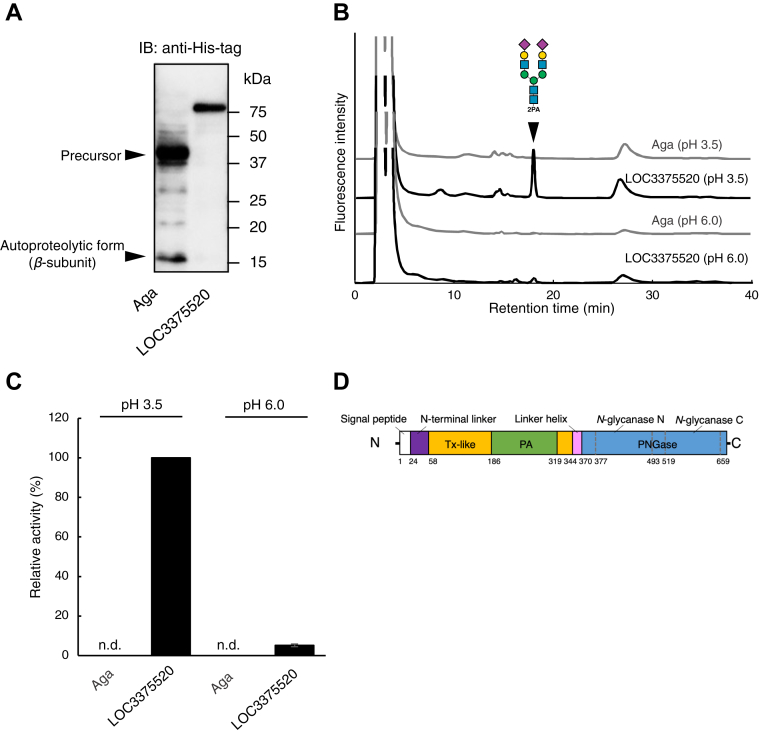
**Identification of acid PNGase in zebrafish.***A,* purified proteins prepared from cells transfected with Aga-His or LOC3375520-His were subjected to Western blotting. *B,* anion-exchange HPLC profile of 2PA-labeled disialylated glycan released by enzymatic reaction. The *black inverted triangle* represents the elution position of 2PA-labeled disialylated glycan (*i.e*., PNGase reaction product). *C,* measurement of PNGase activity toward sialoglycosylated hexapeptide. The peak area of disialylated fraction in LOC3375520 at pH 3.5 was set as 100%. *D,* schematic representation of the domain structure of Ngly2 (LOC3375520). Aga-His, hexahistidine-tagged Aga; PA, protease-associated domain (*green*); PNGase, peptide:*N*-glycanase (N and C domain [PF09112 and PF09113]) (*skyblue*); Tx-like, thioredoxin-like domain (*yellow*).

We initially hypothesized that, rather than there being a novel gene encoding an enzyme exerting acidic PNGase activity, an already-identified enzyme might possess such activity. Specifically, we considered that the substrate specificities of aspartylglucosaminidase (Aga; Enzyme Commission number: 3.5.1.26), another *N*-glycan–cleaving enzyme distinct from PNGases (see below), may have been altered in fish. Aga is a conserved enzyme in vertebrates that removes an l-asparagine moiety from glycosylated asparagine, producing l-aspartic acid, ammonia, and FNGs (36). Aga generally requires a free α-carboxyl and α-amino group on glycosylated asparagine for catalysis to occur (37), and thus, it cannot directly release *N*-glycans from *N*-glycoproteins or glycopeptides. However, in fish, we considered the possibility that Aga may have altered its substrate specificity and, accordingly, may exhibit activity not only toward glycosylated asparagine but also toward glycopeptides. To validate this hypothesis, zebrafish *aga* was cloned, hexahistidine-tagged Aga (Aga-His) was expressed in HeLa cells, and its expression was confirmed by Western blotting (Fig. 1*A*). It was found that zebrafish Aga exhibited de-*N*-glycosylation activity toward glycosylated asparagine (Fig. S1) but did not release sialoglycans from the glycosylated hexapeptide (Fig. 1, *B* and *C*). This suggested that, consistent with the findings for its mammalian counterpart, fish Aga does not possess the acid PNGase activity.

Next, we attempted to identify the acid PNGase through a homology search using known PNGases in other species. Accordingly, homology searches were performed using sequences of PNGase F from *Flavobacterium meningosepticum* (now called *Elizabethkingia meningoseptica*, hereafter referred to as PNGase F) (38) or PNGase A from almond (*Prunus dulcis*) (20) as queries. It should be noted that, unlike acid PNGase in fish, the activity of PNGase F preferentially occurs at neutral to alkaline pH (38). In terms of the primary structure of PNGase F, the protein contains two Pfam domains (*N*-glycanase N and C, PF09112 and PF09113, respectively) (39). Meanwhile, orthologs of almond PNGase A (20), containing a single Pfam domain (PF12222), were found to be widely distributed in plants and also in some fungi (*Aspergillus* and *Eurotiales*), and their activity preferentially occurs at acidic pH (19, 40, 41, 42, 43). We excluded an *ngly1* ortholog in this search, as the zebrafish *ngly1* ortholog has already been characterized and has been shown to be a functional ortholog of mammalian cytosolic, neutral PNGase (44). Overall, our results revealed no protein homologous to PNGase A in the zebrafish genome, but a protein with the same domain as PNGase F (NP_001373269.1) was found (Fig. 1*D*), as reported in a previous study (45). That previous study also indicated that this protein with the same domain as PNGase F is conserved not only in *E. meningoseptica* but also in *Deinococcus radiodurans* (WP_010889584.1), a gram-positive bacterium (45). Interestingly, the uncharacterized protein LOC3375520 possesses a PNGase F domain (amino acids 369–662) composed of PF09112 and PF09113 at its C terminus (Fig. 1*D*). Although PNGase F is not active at acidic pH (38), we nonetheless examined whether LOC3375520 exhibits acid PNGase activity.

To characterize the protein LOC3375520, the gene encoding was amplified using complementary DNA (cDNA) prepared from zebrafish-derived BRF41 cells. Comparing the amino acid sequences between the predicted and cloned LOC3375520 sequences, the presence of two nonsynonymous mutations (A461P and T618R) was noted. While the amino acid sequence deduced for protein LOC3375520 showed relatively low similarity to PNGase F (24%), the active site of PNGase F (Asp60 and Glu206 [46]) was conserved (Fig. S2), implying that, despite the drastic difference in their pH profile, the LOC3375520 warranted testing for PNGase activity.

Western blotting analysis using His-tag antibody with the purified recombinant LOC3375520 prepared from HeLa cells revealed a single band corresponding to a mass greater than 75 kDa, which is larger than the size predicted from the deduced amino acids (74.5 kDa) (Fig. 1*A*). The purified protein was subjected to the PNGase assay, and as shown in Figure 1*C*, it clearly exhibited PNGase activity at pH 3.5, but the activity was hardly detected at pH 6.0 (Fig. 1, *B* and *C*), which is consistent with the properties of native medaka acid PNGase (26). This result was somewhat counterintuitive, as the optimal pH for acid PNGase (pH 3.5–4.5) is distinct from that for PNGase F (pH 7.5–9) (38). Nevertheless, these results clearly indicated that the protein LOC3375520 is a *bona fide* acid PNGase. We thus named this protein Ngly2 and further characterized its enzymatic properties.

### Ngly2 is found not only in fish but also in many other aquatic organisms

A homology search of Ensembl genome browser 111 (https://asia.ensembl.org/index.html) was conducted using the zebrafish *ngly2* gene as a query to examine the phylogenetic distribution of *ngly2*. The results revealed no *ngly2* orthologs in Tetrapoda. Meanwhile, *ngly2* orthologs were found in 58 ray-finned fish species (Table S1). Among them, the presence of two *ngly2* orthologs was predicted in five species (brown trout, Chinook salmon, large yellow croaker, pikeperch, and rainbow trout). An ortholog of *ngly2* was also found in elephant shark, coelacanth, hagfish, and lamprey. These results clearly indicate that Ngly2, in sharp contrast to the case for Ngly1, is an enzyme specific to fish but not found in other vertebrates. Next, multiple sequence alignment analysis was carried out using Clustal Omega. The sequence alignment showed a high degree of similarity between zebrafish Ngly2 and other fish Ngly2 proteins (Fig. S2). While the N-terminal region of Ngly2 was diverse, the putative catalytic residues (Asp and Glu) in the C-terminal PNGase domain were found to be completely conserved among fish species (Fig. S2).

Homology searches for *ngly2* in Metazoa identified orthologous genes in Homoscleromorpha, Calcarea, Enteropneusta, Echinoidea, Holothuroidea, Leptocardii, Ascidiacea, Eutardigrada, Lingulata, Polychaeta, Cephalopoda, Polyplacophora, Bivalvia, Anthozoa, Scyphozoa, and Hydrozoa (Fig. S3). These results clearly indicated that Ngly2 is conserved not only in fish but also in other animal/bacteria species, particularly those that inhabit aquatic environments.

### Enzymatic characterization of Ngly2

**Figure 2:**
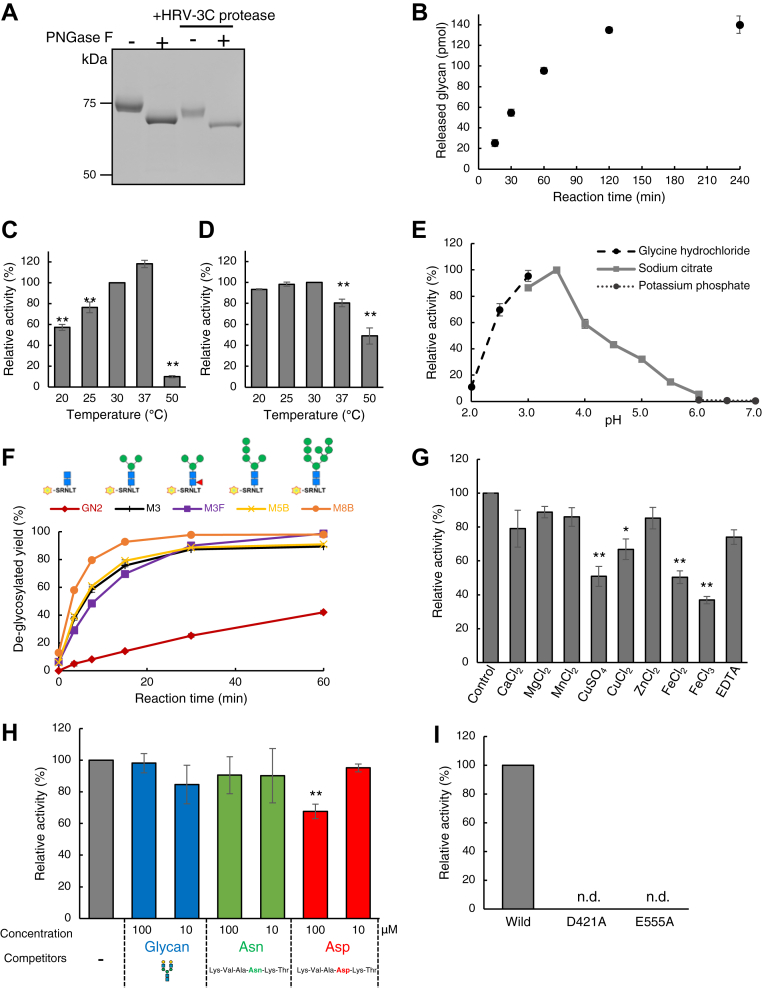
**Enzymatic properties of zebrafish Ngly2.***A,* CBB staining of purified recombinant proteins. Purified Ngly2 was treated by PNGase F and/or 3C protease. *B,* time-course analysis of PNGase activity. assay was carried out at 30 °C. *C,* effect of temperature. Ngly1 was assayed using 5-FAM GH at various temperatures for 30 min. Activity at 30 °C was set to 100%. *D,* thermal stability. After preincubation at various temperatures for 1 h, PNGase activity assay using 5-FAM GH was carried out at 30 °C for 30 min. Activity at 30 °C was set to 100%. *E,* effect of pH. Ngly2 was assayed using 5-FAM GH at various pH (glycine hydrochloride buffer [pH 2.0–3.0], sodium citrate buffer [pH 3.0–6.0], and potassium phosphate buffer [pH 6.0–7.0]). *F,* substrate specificity of Ngly2. Ngly2 was assayed at 30 °C for 60 min toward various substrates. *G,* effect of metal ions. Ngly2 was assayed using 5-FAM GH in the presence of indicated compounds (5 mM). Activity without compound was set to 100%. *H,* effect of peptide/glycans. Ngly2 was assayed using 5-FAM GH with various concentrations of the following compounds (Asn-peptide: Lys-Val-Ala-Asn-Lys-Thr, Asp-peptide: Lys-Val-Ala-Asp-Lys-Thr, and asialobiantennary glycan). Activity without compound was set to 100%. *I,* effect of mutations on the putative active sites. Ngly2 was assayed using WT protein as well as its point mutants, D421A and E555A. Activity of WT Ngly2 was set to 100%. These results are shown as mean ± standard deviation from three independent experiments. For (*C*), (*D*), (*G*), and (*H*), unpaired *t* test was used for statistical analysis. ∗*p* < 0.05, ∗∗*p* < 0.01, compared with 30 °C for (*C*) and (*D*) or control for (*G*) and (*H*). CBB, Coomassie Brilliant Blue; 5-FAM GH, 5-carboxyfluorescein-labeled glycosylated hexapeptide; PNGase, peptide:*N*-glycanase.

For the enzymatic characterization of Ngly2, it was expressed using an insect cell-baculovirus system, and the protein was purified through a series of chromatographic steps. The elution position by a gel filtration column was found to be around 150 kDa at both pH 4.0 and pH 8.0 (Fig. S4, *A* and *B*), implying that Ngly2 may form a homodimer under both acidic and basic conditions. No monomeric form was detected under either condition. This size is somewhat consistent with the medaka acid PNGase (26). As shown in Figure 2*A*, a single band of Ngly2 was detected, indicating that our three-step purification yielded a highly purified Ngly2 protein. The band associated with PNGase F treatment shifted to a lower molecular weight, indicating that Ngly2 possesses *N*-glycans. This is consistent with the finding that the Ngly2 protein expressed in HeLa cells also showed slower migration than expected when considering its predicted size (Fig. 1*A*). After the cleavage of Strep tag by 3C protease and PNGase F digestion, the single band of Ngly2 coincided with the size as predicted from the deduced amino acids (Fig. 2*A*). This pure Ngly2 protein was subjected to a standard PNGase assay using 5-carboxyfluorescein-labeled glycosylated hexapeptide (5-FAM GH). Time-course analysis of the PNGase activity using 5-FAM GH showed a linear rate up to 60 min (Fig. 2*B*). The reaction rate of Ngly2 under these reaction conditions was 12 nmol min^−1^ mg^−1^.

Next, the effects of temperature on the PNGase activity and its thermal stability were examined. While the activity of Ngly2 peaked at 37 °C for 30 min incubation, preincubation at 37 °C for 1 h resulted in a significant reduction of enzyme activity (Fig. 2, *C* and *D*). On the basis of these results, further measurement of PNGase activity was carried out at 30 °C for 30 min. Zebrafish Ngly2 exhibited maximal PNGase activity under acidic conditions, with the highest activity observed at pH 3.5 (Fig. 2*E*). In contrast, virtually no activity was detected at pH above 6.

We also investigated its substrate specificities regarding the glycan structures. To this end, various pentapeptides (Ser-Arg-Asn-Leu-Thr) with a variety of glycan structures were prepared through chemical synthesis. As shown in Figure 2*F*, Ngly2 activity was comparable for all substrates, except for the peptide having a di-*N*-acetylchitobiose structure, which showed somewhat compromised reactivity. These results indicate that Ngly2 exhibits relatively broad substrate specificity toward glycan structures. Notably, its activity was not affected by the presence of α1-6-linked core fucose, in contrast to mammalian Ngly1, which shows no activity against core α1,6-fucosylated substrates (23).

A previous study reported that medaka acid PNGase activity was inhibited by the presence of 5 mM FeCl_3_ or CuSO_4_ (26). We thus investigated the effect of metal ions on the PNGase activity of zebrafish Ngly2. It was found to be significantly decreased in the presence of CuSO_4_, CuCl_2_, FeCl_2_, and FeCl_3_ (Fig. 2*G*). Medaka acid PNGase was also shown to be strongly inhibited by a deglycosylated peptide (26). The effect of PNGase-deglycosylated peptide on zebrafish Ngly2 was therefore investigated using two types of hexapeptides (Asn-peptide: Lys-Val-Ala-Asn-Lys-Thr, representing the “unglycosylated form,” and Asp-peptide: Lys-Val-Ala-Asp-Lys-Thr, representing the “PNGase-deglycosylated form” of the substrate used). As shown in Figure 2*H*, the high concentration of Asp-peptide (100 μM) significantly inhibited the enzymatic activity of Ngly2. However, the degree of inhibition by a PNGase-deglycosylated peptide was much less pronounced compared with the medaka enzyme. This apparent discrepancy may be due to the use of a nonphysiological synthetic substrate in our assay, whereas the medaka enzyme was tested using a hyosophorin-derived product peptide, which likely serves as its endogenous substrate (26).

Notably, PNGase F, a distant homolog of Ngly2, exerts its activity under neutral to alkaline conditions, at which Ngly2 is nearly completely inactive, despite the fact that the catalytic residues for PNGase F are conserved in Ngly2 (Fig. S2). We therefore tested whether the residues equivalent to the catalytic residues of PNGase F are also essential for Ngly2. We successfully purified both Ngly2 active site mutants (Asp421Ala and Glu555Ala), similar to the case for WT Ngly2 protein (Fig. S4*C*). As shown in Figure 2*I*, both the Ngly2 active site mutants resulted in the complete loss of activity. These results clearly indicated that, while there is a quite distinct pH profile between PNGase F and Ngly2, they do appear to share the same catalytic residues.

### Dimer structure of zebrafish Ngly2 as revealed by cryo-EM analysis

**Figure 3:**
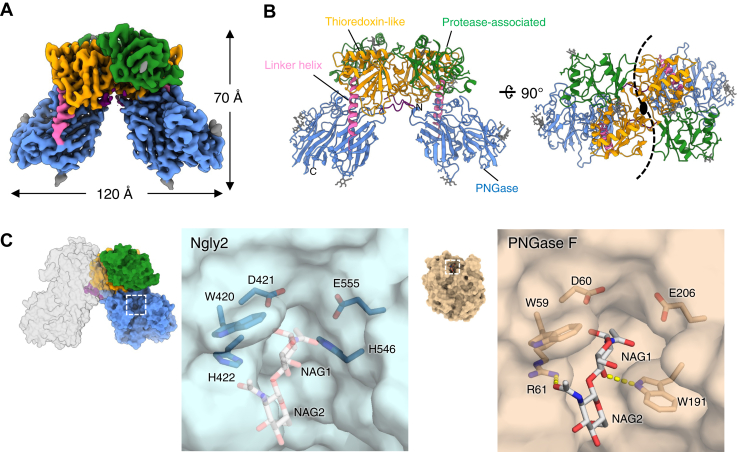
**Cryo-EM structure of zebrafish Ngly2 and comparison of its active site with PNGase F.***A,* cryo-EM density map of Ngly2 at 2.7 Å resolution. *B, cartoon representation* of the Ngly2 dimer viewed perpendicular to the twofold symmetry axis. The thioredoxin-like domain (Tx-like), the protease-associated domain (PA), the linker helix, and the PNGase domain are colored *yellow*, *green*, *pink*, and *sky blue*, respectively (as shown in Fig. 1*D*). *N*-linked glycans are partially modeled at four of seven predicted *N*-glycosylation sites are shown in *gray*. The *black dashed line* indicates the dimer interface. *C,* comparison of the active sites of Ngly2 and PNGase F (PDB ID: 1PNF, *wheat colored*). In the PNGase F structure, the cleaved product *N,N′*-diacetylchitobiose (GlcNAcβ1–4GlcNAc) is bound on the surface of the active site. In the apo Ngly2 structure, the same product is superimposed and displayed in transparent coloring. PDB, Protein Data Bank; PNGase, peptide:*N*-glycanase.

To elucidate the structural basis by which Ngly2 regulates its catalytic activity, we performed single-particle cryo-EM analysis. The 3D reconstruction, conducted with imposed *C2* symmetry, yielded a density map at an average nominal resolution of 2.7 Å. The resulting structure, shown in Figure 3, *A* and *B*, clearly forms a homodimer. At the electron density map, the N terminus starts at residue 51, whereas no interpretable density was observed for the preceding N-terminal region. Structural homology analysis using the Dali server revealed that Ngly2 possesses two additional domains in the N-terminal region, located upstream of the PNGase domain. One of these domains, composed of residues 58 to 185 and 319 to 343, exhibits significant structural similarity to a thioredoxin-like (Tx-like) domain found in peroxiredoxins (Figs. 1*D* and 3*B*, and S5*A*). However, the Tx-like domain in Ngly2 lacks the redox-active Cys-X-X-Cys motif (Fig. S2), suggesting that it does not function as an oxidoreductase. The other region, spanning residues 186 to 318, is identified as a protease-associated (PA) domain, showing structural similarity to domains found in certain peptidases (Figs. 1*D* and 3*B*, and S5*B*). A helical segment between the Tx-like and PNGase domains acts as a linker connecting these regions (Fig. 3, *A* and *B*).

Ngly2 homodimerizes primarily through interactions between the Tx-like and PA domains. Around the twofold axis, the Tx-like domains dimerize *via* extensive hydrophobic interactions (Fig. S5*C*), burying approximately 1500 Å^2^ of solvent-accessible surface area upon dimer formation. This structural observation is consistent with the results obtained from gel filtration chromatography (Fig. S4*A*).

### Structural basis of the catalytic site in Ngly2

The folding of the PNGase domain of Ngly2 is typical of PNGase family (45). The domain consists of two jelly-roll subdomains stabilized by hydrophobic core residues and disulfide bonds. Its overall structure is nearly identical to that of PNGase F (46), and the catalytic residues essential for the enzymatic activity are conserved in Ngly2 (Asp421 and Glu555) (*asterisks* in Figs. S2; 3*C*). In PNGase F, Trp59, Arg61, and Trp191 contribute to capturing the di-*N*-acetylchitobiose moiety of the glycan substrate near the active site (46, 47). In Ngly2, Trp420 corresponding to Trp191 of PNGase F is conserved and is expected to participate in hydrophobic interactions. In contrast, Trp59 and Arg61 are replaced by histidines, His422 and His546, respectively (Fig. 3*C*). These histidines could form hydrogen bonds with the chitobiose *via* protonated imidazole groups. Consistently, Ngly2 exhibits enzymatic activity at acidic pH (below 6), where histidine residues are likely protonated (Fig. 2*E*). To further explore the catalytic mechanism, we modeled the complex of Ngly2 bound to an *N*-glycosylated peptide by AlphaFold 3 (48). In the predicted structure, the whole glycan substrate is positioned just above the active site, and the peptide portion of the substrate is located in a deep cleft between the PA domain and the PNGase domain (Fig. S5*D*). Therefore, the linear peptide is likely accommodated within the cleft just above the active site. The model implies that the PA domain contributes to substrate binding and may regulate enzyme activity by stabilizing this interaction. Indeed, when a glycosylated cyclic heptapeptide (49) was used as a substrate in activity assays, Ngly2 showed no enzymatic activity (Fig. S4*D*). In contrast, PNGase F, which lacks the additional PA and Tx-like domains, exhibited clear activity (Fig. S4*D*). In addition, Trp480 of Ngly2 was predicted to play a key role in stabilizing NAG2 to BMA3 moieties within the model (Fig. S5*D*). To validate this, we prepared a W480A mutant and measured its enzymatic activity. The mutant retained only a feeble activity (Fig. S4*E*), highlighting the functional importance of Trp480 in substrate recognition or catalysis. This finding is also consistent with the aforementioned observation that Ngly2 shows higher catalytic efficiency against glycan chains with β-mannose residue (Fig. 2*F*).

### Lysosomal localization of Ngly2

**Figure 4:**
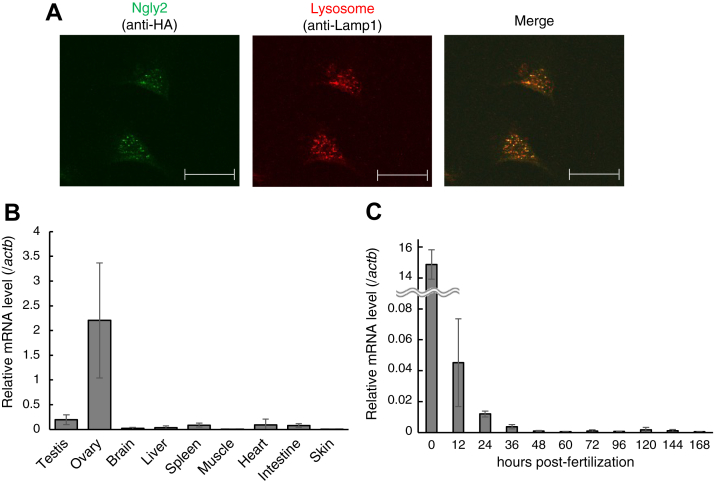
**Subcellular localization of Ngly2 in BRF41 cells and spatiotemporal expression of *ngly2* in zebrafish.***A,* subcellular localization was analyzed by indirect immunofluorescence staining in BRF41 cells transfected with zebrafish *ngly2*-HA. The scale bar represents 25 μm. *B,* tissue distribution of *ngly2* expression as determined by real-time PCR. The values are expressed as a relative level normalized with a house keeping gene (*actb*). *C,* expression profile of *ngly2* during zebrafish early developmental stages was determined by real-time PCR. The values are expressed as a relative level normalized with a house keeping gene (*actb*). These results are shown as mean ± standard deviation from four biological replicates.

To verify the subcellular localization, *ngly2*-hemagglutinin (HA) was transiently transfected into BRF41 cells. As shown in Figure 4*A*, Ngly2 was found to colocalize with Lamp1, a lysosomal marker. This suggested that zebrafish Ngly2 is localized in the lysosomes, in accordance with its preference for acidic pH (Fig. 2*E*).

### High mRNA expression of *ngly2* in the ovary and fertilized eggs

To determine the tissue distribution of zebrafish *ngly2*, quantitative real-time PCR was carried out. While the expression of *ngly2* was detected in tested tissues from adult zebrafish, the level of expression was found to be far higher in the ovary (Fig. 4*B*). Furthermore, the expression of *ngly2* was investigated during embryonic and early larval development in zebrafish. The level of *ngly2* mRNA, most likely maternal, was very high in the fertilized eggs, but after fertilization, the level remained relatively low during development (Fig. 4*C*). The mRNA of *ngly2* appeared to be rapidly degraded after fertilization. These results suggested that Ngly2 plays a role in rather specific tissues and developmental stages, namely, the ovary and fertilized eggs.

### *ngly2*-KO zebrafish exhibited normal development

**Figure 5:**
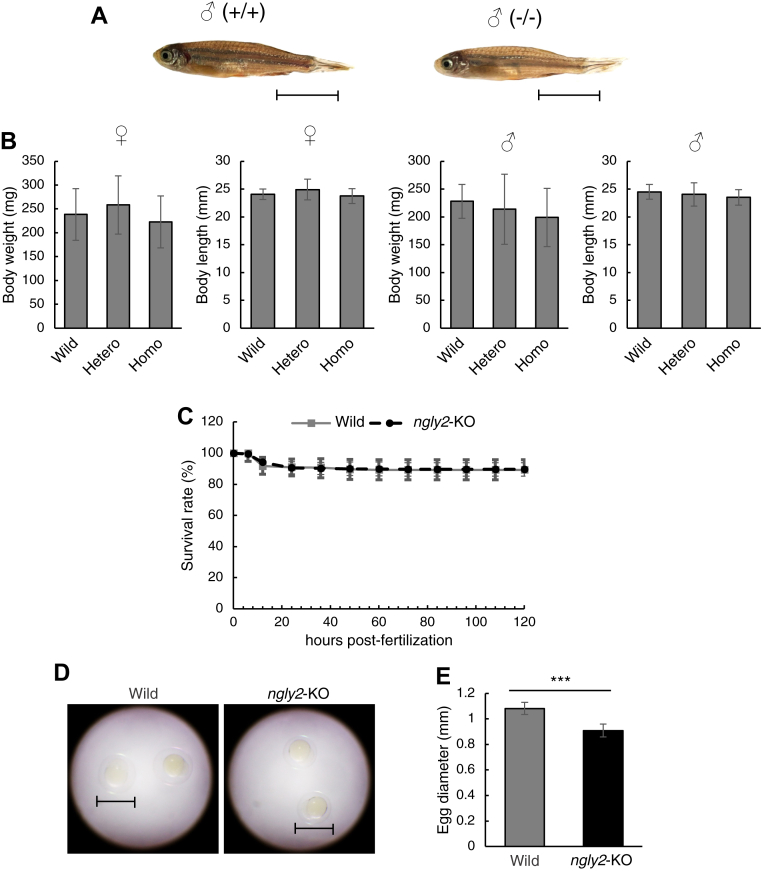
**Phenotype analysis of *ngly2*-KO zebrafish.***A,* morphological observations of WT (+/+) and *ngly2*-KO zebrafish (−/−) in 8 months. The scale bar represents 10 mm. *B,* body weight and body length of WT (+/+) (♀ n = 7, ♂ n = 11), hetero (+/−) (♀ n = 20, ♂ n = 31), and *ngly2*-KO (−/−) (♀ n = 10, ♂ n = 12). These results are shown as mean ± standard deviation. *C,* survival rate of maternal–zygotic mutant of *ngly2*-KO zebrafish. *D,* morphological observation of WT and *ngly2*-KO zebrafish-fertilized eggs. The scale bar represents 1 mm. *E,* size measurement of WT (n = 61) and *ngly2*-KO (n = 74) zebrafish-fertilized eggs. These results are shown as mean ± standard deviation from independent samples. Unpaired *t* test was used for statistical analysis. ∗∗∗*p* < 0.0001.

To further explore the physiological function of Ngly2, we generated an *ngly2*-KO zebrafish strain using CRISPR–Cas9 gene engineering to edit the *ngly2* gene. To avoid off-target effects as much as possible, two single-guide RNAs (sgRNAs) were designed using CRISPRdirect (50) (Fig. S6*A*). The mutations resulted in a frameshift within the open reading frame and an inactive enzyme (Fig. S6*B*). The F_0_ founder was crossed with WT zebrafish to obtain the F_1_ generation. Two F_1_ pairs bearing an indel mutation, as confirmed by DNA sequencing, in the coding region were then used to obtain the F_2_ generation. Genotyping analysis of F_2_ fish was then carried out by agarose gel electrophoresis (Fig. S6*C*). Macroscopic analysis indicated that the homozygous KO fish did not show any morphological abnormalities and reached adulthood with no overt phenotypes (Fig. 5*A*). There was no significant difference in body weight or length of homozygotes (at ∼8 months old) compared with heterozygotes and the WT fish (Fig. 5*B*). In addition, the homozygous mutant could be maintained by crossing homozygous males and females, indicating that the homozygous fish were fertile.

### Disappearance of free glycans in smaller fertilized eggs of *ngly2*-KO zebrafish

**Figure 6:**
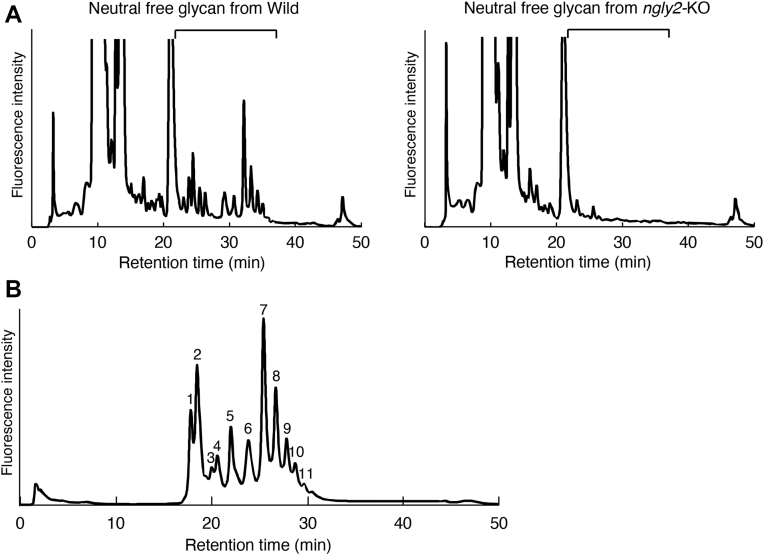
**Disappearance of free glycans in fertilized eggs of *ngly2*-KO zebrafish.***A,* size-fractionation HPLC profile of 2PA-labeled neutral free glycans with glucoamylase-treated samples. Free *N*-glycan fractions were indicated by *square bracket* and were subjected to LC–MS analysis. *B,* LC–MS HPLC profile of fractionated 2PA-labeled neutral free glycans from the fertilized eggs of WT. Numbered peaks were subjected to MS analysis (Table 1).

For further detailed analysis, the maternal–zygotic mutant obtained by crossing homozygous females and males was used. The survival rate of the *ngly2*-KO maternal–zygotic mutant was comparable to that of WT zebrafish (Fig. 5*C*). However, the fertilized eggs of the *ngly2*-KO strain were significantly smaller than those of the WT strain (Fig. 5, *D* and *E*). Furthermore, the level of free glycans prepared from the fertilized eggs of *ngly2*-KO zebrafish was greatly reduced in both neutral and acidic fractions (Figs. 6*A* and S7), clearly indicating that the formation of free glycans is impaired by the absence of Ngly2 proteins. Interestingly, many peaks of acidic-free glycans observed in diethylaminoethyl (DEAE) anion exchange HPLC did not disappear upon sialidase treatment but could be removed by TFA treatment (Fig. S7). This suggests that these free glycans in zebrafish-fertilized eggs included sialidase-resistant species, such as glycans with phosphate, sulfate, KDN, or modified sialic acid resistant to sialidase (*e.g.*, *O*-acetylation, apart from a 9-*O*-acetyl group or 8-*O*-sulfation). To perform further structural analysis, we used the neutral-free glycan fractions from both WT and *ngly2*-KO strains.

To investigate the detailed glycan structures, the fractionated neutral fractions were analyzed by LC–MS. In the *ngly2*-KO zebrafish, essentially no glycans were detected (Fig. S8), whereas in the WT, various glycans were observed, the structures of which were similar to the free glycans in zebrafish embryos reported previously (17) (Fig. 6*B* and Table 1; detailed mass spectrometry (MS) and HPLC data are shown in Figs. S9–S13). In this study, most of the observed glycans were found to be of the Gn1 type. It should be noted that the Galβ1–4Gal structure showed resistance to β1-3,4 galactosidase from bovine testis and additionally conferred resistance to α1-3,4 fucosidase on the Fucα1–3GlcNAc.

**Table 1:** Neutral free oligosaccharides observed in zebrafish-fertilized eggs

Peak ID	Observed MS	Composition	Ion species	Theoretical MS	Estimated structure
1	1135.4598 1192.4730	Hex_3_HexNAc_2_dHex_1_+2PA Hex_3_HexNAc_3_+2PA	[M+H]^+^ [M+H]^+^	1135.451 1192.474	
2	1135.4595 1395.5589	Hex_3_HexNAc_2_dHex_1_+2PA Hex_3_HexNAc_4_+2PA	[M+H]^+^ [M+H]^+^	1135.451 1395.552	
3	660.2512 668.2391 688.7627 696.7503 1297.5136 1354.5411	Hex_4_HexNAc_2_dHex_1_+2PA Hex_4_HexNAc_2_dHex_1_+2PA Hex_4_HexNAc_3_+2PA Hex_4_HexNAc_3_+2PA Hex_4_HexNAc_2_dHex_1_+2PA Hex_4_HexNAc_3_+2PA	[M+H+Na]^2+^ [M+H+K]^2+^ [M+H+Na]^2+^ [M+H+K]^2+^ [M+H]^+^ [M+H]^+^	660.234 668.234 688.757 696.744 1297.504 1354.525	
4	660.2530 668.2405 1297.5134	Hex_4_HexNAc_2_dHex_1_+2PA Hex_4_HexNAc_2_dHex_1_+2PA Hex_4_HexNAc_2_dHex_1_+2PA	[M+H+Na]^2+^ [M+H+K]^2+^ [M+H]^+^	660.234 668.234 1297.504	
5	761.7938 769.7802 1500.5938	Hex_4_HexNAc_3_dHex_1_+2PA Hex_4_HexNAc_3_dHex_1_+2PA Hex_4_HexNAc_3_dHex_1_+2PA	[M+H+Na]^2+^ [M+H+K]^2+^ [M+H]^+^	761.786 769.773 1500.583	
6	842.8189 850.8073 1662.6520	Hex_5_HexNAc_3_dHex_1_+2PA Hex_5_HexNAc_3_dHex_1_+2PA Hex_5_HexNAc_3_dHex_1_+2PA	[M+H+Na]^2+^ [M+H+K]^2+^ [M+H]^+^	842.813 850.800 1662.636	
7	915.8513 923.8371 1808.7066	Hex_5_HexNAc_3_dHex_2_+2PA Hex_5_HexNAc_3_dHex_2_+2PA Hex_5_HexNAc_3_dHex_2_+2PA	[M+H+Na]^2+^ [M+H+K]^2+^ [M+H]^+^	915.842 923.829 1808.694	
8	996.8770 1004.8647	Hex_6_HexNAc_3_dHex_2_+2PA Hex_6_HexNAc_3_dHex_2_+2PA	[M+H+Na]^2+^ [M+H+K]^2+^	966.868 1004.855	
9	1077.9031 1085.8902	Hex_7_HexNAc_3_dHex_2_+2PA Hex_7_HexNAc_3_dHex_2_+2PA	[M+H+Na]^2+^ [M+H+K]^2+^	1077.894 1085.881	
10	1171.4472 1179.4343	Hex_6_HexNAc_4_dHex_3_+2PA Hex_6_HexNAc_4_dHex_3_+2PA	[M+H+Na]^2+^ [M+H+K]^2+^	1171.437 1179.424	
11	1252.4726 1260.4619	Hex_7_HexNAc_4_dHex_3_+2PA Hex_7_HexNAc_4_dHex_3_+2PA	[M+H+Na]^2+^ [M+H+K]^2+^	1252.463 1260.450	

The MS results for each fraction separated by HPLC shown in Figure 6*B* are summarized. The peak ID in the table is the peak number on the HPLC profile in Figure 6*B*. Note that the proximal GlcNAc residue has been reduced upon labeling with 2PA.

## Discussion

While the basic biosynthetic–catabolic pathways of glycans have been extensively studied in organisms, such as budding yeast and mammals, pathways in other organisms can be species specific and have remained poorly understood outside these organisms. In this study, we identified a gene encoding a novel *N*-glycanase in zebrafish, which we named Ngly2. This corresponds to the first animal-origin PNGase described in the 1990s (21, 26). Among vertebrates, the *ngly2* gene is widely distributed only in fish, including coelacanth, hagfish, and lamprey, and is thus expected to be involved in rather fish-specific glycan catabolism. In addition to vertebrates, the *ngly2* gene ortholog was found in Porifera, Cnidaria, Echinodermata, Chordata, Mollusca, and even in some bacteria. Many aquatic organisms possess *ngly2*, suggesting that Ngly2 may be somehow involved in adaptation to aquatic environments.

Zebrafish Ngly2 was found to be composed of three main domains: Tx-like domain, PA domain, and PNGase domain (Figs. 1*D* and 3, *A* and B). In zebrafish Ngly2, the Tx-like domain is devoid of the redox-active Cys-X-X-Cys motif (51), and the absence of this motif is conserved among fish Ngly2s (Fig. S2). According to the cryo-EM results, the Tx-like domain is involved in homodimer formation. Notably, a previous report indicated that a Tx-like domain lacking an active site in ERp29 acts as a homodimerization module (52). The PA domain is found in proteases, such as subtilase (peptidase family S8) and metalloproteases (peptidase families M20, M28, and M33), mammalian transferrin, and plant vacuolar-sorting receptors (53, 54). It is an ancillary domain that probably facilitates its interaction with itself or other proteins, such as substrates, inhibitors, and receptors, or plays a regulatory role in its functions (53, 54, 55). Our structural analysis (Fig. S5*C*) showed that the PA domain in zebrafish Ngly2 is involved in homodimer formation, consistent with a report on tomato subtilase 3 (56). The PA domain in zebrafish Ngly2 also appears to regulate substrate specificity because it is located right next to the active site of Ngly2 (Fig. S5*D*).

In the PNGase domain of zebrafish Ngly2, active sites in common with PNGase F were identified. The alanine mutants of active site residues completely lost their activity (Fig. 2*I*), as was the case for PNGase F (46). Despite them sharing common active sites, Ngly2 showed different optimal pH from PNGase F (Fig. 2*E*). Histidine residues (H422 and H546) uniquely present in the vicinity of the active site of Ngly2 may contribute to its activity under acidic conditions. Further structural analysis, such as of complexes between Ngly2 and substrates, will be required to resolve why Ngly2, but not PNGase F, is active under acidic conditions. To explore potential structural similarities, we compared Ngly2 with the bacterial acidic PNGase H^+^ from *Terriglobus roseus* (57) and found that the sequence identity was low (19.63%). It is worth noting here that PNGase H+ has rather similarity with PNGase A (plant acid PNGase) (57). In this regard, it would be interesting to obtain the pH profiles of bacterial PNGase orthologs other than PNGase F. It is possible that some of these bacterial PNGases exhibit pH preferences similar to that of Ngly2. In this connection, a recent report showed that *Flavobacterium akianinvivens–*derived PNGase L, which has similar active site with Ngly2 or PNGase F, exhibited optimal pH at 6.0 (58).

Zebrafish Ngly2 was active only under acidic conditions (Fig. 2*E*). This is a typical property of lysosomal enzymes, and indeed, Ngly2-HA was shown to colocalize with lysosomal Lamp1 (Fig. 4*A*). These results indicated that fish Ngly2 is involved in the deglycosylation of glycoproteins or glycopeptides in lysosomes. However, we can safely assume that it does not function in general glycoprotein catabolism, as the KO animals did not show any overt phenotypes, whereas a defect in a lysosomal glycan catabolic enzyme generally results in phenotypes typical of lysosomal storage diseases, such as curved vertebrae and a small body size/length (59, 60). Instead, Ngly2 may be involved in the deglycosylation of specific substrates, such as hyosophorin or glycophosphoprotein (phosvitin) (26). Notably, both hyosophorin and glycophosphoprotein undergo proteolytic processing during fertilization and oogenesis, respectively (61, 62). It is therefore tempting to speculate that the deglycosylation event facilitates the subsequent proteolytic processing, as proposed previously (63, 64, 65). During this process, Asn-to-Asp conversion by Ngly2 may be critical, at least in a certain biological context. In this regard, it should also be noted that this enzyme was found to be inactive against glycosylated cyclic heptapeptide (49) (Fig. S4*D*), despite the fact that this Ngly2 appears to have broad substrate specificity regarding the glycan structures (Fig. 2*F*). This result is somewhat consistent with a previous study showing that medaka acid PNGase exerted its activity toward hyosophorin and glycophosphoprotein but not toward other intact glycoproteins such as fetuin and ovalbumin (26). Hyosophorin has very high glycan content (>90%), and glycophosphoproteins are highly phosphorylated, and thus in both cases, the protein part may not form a specific secondary structure. This may be critical for the substrate to fit into the cleft of Ngly2 in which the catalytic residues are located, as shown by the predicted complex with the substrate (Fig. S5*D*). The PA domain above the active site probably maintains the protein in a rigid 3D structure to prevent access to the active sites. Because the cyclopeptide clearly has a structural constraint on the peptide part, it will have difficulty fitting into the substrate-binding groove.

In the case of vitellogenin, it is produced in the liver and then transported into the ovaries *via* the bloodstream. Vitellogenin incorporated by vitellogenin receptor–mediated endocytosis is proteolyzed by cathepsin D during oocyte growth, forming glycophosphoproteins (66, 67). It is tempting to speculate that glycans on vitellogenin protect core proteins from proteases during transport and/or influence affinity with receptors. After uptake, the removal of *N*-glycans by Ngly2 could provide the specific proteases with easy access to the vitellogenin, thus regulating the timing of processing. In fact, the mRNA expression of *ngly2* was predominantly detected in the ovary (Fig. 4*B*).

To clarify the physiological function of Ngly2 in zebrafish, we generated *ngly2*-KO zebrafish. The fertilized eggs from *ngly2*-KO maternal–zygotic mutants were significantly smaller than those of the WT, although the survival rate was not affected. Moreover, free glycans from *ngly2*-KO zebrafish disappeared almost completely. Therefore, one could safely assume that zebrafish Ngly2, not Ngly1, is involved in the generation of free glycans in fertilized eggs. At present, it remains unclear how the disappearance of free glycans affects the fertilized egg size, and further studies will be required to clarify their functional importance. LC–MS analysis revealed the detailed structures of neutral free oligosaccharides from WT fertilized eggs. The unique glycan structures were consistent with those found in previous analyses of free glycans in zebrafish embryos and glycomes in adult zebrafish (Table 1) (17, 68). In future works, it would be interesting to clarify the functional importance of Ngly2 to shed light on the evolutionary conservation of this enzyme in aquatic organisms, not only in the animal kingdom but also in bacteria.

## Experimental procedures

### Animal

RIKEN WT (RW) zebrafish were used in this study, unless noted otherwise (RIKEN-Wako). The fish were maintained in 7 l tanks with continuous water exchange at 28.5 °C under a 14 h light–10 h dark cycle. These fish were fed brine shrimp twice a day. Fertilized eggs were obtained from natural crosses. The obtained fertilized eggs were kept in breeding water with 10^−5^% methylene blue. The fish were anesthetized with 0.1% tricaine, and tissues were immediately excised, followed by storage at −80 °C until use. All protocols were reviewed and approved by the Animal Care and Use Committee of RIKEN.

### Cell culture

HeLa cells were maintained in Dulbecco's modified Eagle's medium (Nacalai Tesque; catalog no.: 08457-55) containing 10% fetal bovine serum (Nichirei; catalog no.: 175012, Lot no.: 19G00F) with 5% CO_2_ at 37 °C. BRF cells (zebrafish fin fibroblasts) were cultured in Leibovitz's L-15 medium (FUJIFILM Wako Pure Chemical Corporation; catalog no.: 128-06075) with 15% fetal bovine serum (Sigma–Aldrich; catalog no.: 173012, Lot no.: 0001653208) at 33 °C. Sf9 cells (Gibco; catalog no.: B82501) were cultured in Sf-900III SFM (Thermo Fisher Scientific; catalog no.: 1258027) supplemented with penicillin–streptomycin mixed solution (Nacalai Tesque; catalog no.: 26253-84). Transfection was carried out using FuGENE HD Transfection Reagent (Promega; catalog no.: E2311), in accordance with the manufacturer's instructions.

## Method details

### *In silico* analysis

To search for a PNGase gene candidate in the zebrafish genome, PNGase F from *E. meningoseptica* (GenBank: J05449.1) or PNGase A from *P. dulcis* (GenBank: BBG96203.1) was used as a query for a homologous protein search using Protein BLAST (https://blast.ncbi.nlm.nih.gov/Blast.cgi?PROGRAM=blastp&PAGE_TYPE=BlastSearch&LINK_LOC=blasthome). Once we identified the *ngly2* gene, its cDNA sequence was used to search for orthologous genes in other vertebrates using the Ensembl genome browser (https://asia.ensembl.org/index.html) and using BLAST for Metazoa. Assembly of the deduced nucleotide and amino acid sequences was carried out using Clustal Omega (https://www.ebi.ac.uk/jdispatcher/msa/clustalo). The Dali server (http://ekhidna2.biocenter.helsinki.fi/dali/) was used to analyze the domain structure of Ngly2 (69). The phylogenetic tree information of Metazoa was obtained from TimeTree 5 (http://timetree.org) (70). The construction and editing of the phylogenetic tree were performed using TreeViewer (developed by Giorgio Bianchini and Patricia Sánchez‑Baracaldo) (71).

### RNA extraction and cDNA synthesis

Total RNA was extracted from BRF41 cells, adult tissues, or whole embryos using RNeasy mini kit (QIAGEN; catalog no.: 74134), followed by the synthesis of cDNAs using SuperScript IV First-Strand Synthesis system (Invitrogen; catalog no.: 1809115), in accordance with the manufacturer's instructions.

### Molecular cloning of zebrafish *aga* and *ngly2*

Zebrafish *aga* and *ngly2* (si:dkey-256h2.1) were cloned using gene-specific primers based on the nucleotide sequence deposited in GenBank with accession numbers NM_001110281 and NM_001386340.1, respectively. The cDNA derived from BRF41 was used for the amplification of *aga* and *ngly2* genes using Prime STAR Max DNA Polymerase (Takara;catalog no.: R045A) with the specific primers 5′-CAGTGTGGTGGAATTATGATTCAGCGATTATTTATCA-3′ and 5′-TAGACTCGAGCGGCCTTAAAAGCAGTTCACTGTATG-3′ for *aga* and 5′-CAGTGTGGTGGAATTATGTTTTATTGTCGATTTCTGC-3′ and 5′-TAGACTCGAGCGGCCTCATTGATAAAACACTAGATAG-3′ for *ngly2*, respectively. cDNA thus amplified was subcloned into the pcDNA 3.1 plasmid (Invitrogen; catalog no.: V79020), and its sequence was confirmed using the ABI 3730xl DNA analyzer at the Support Unit for Bio-Material Analysis in RIKEN Center for Brain Science, Research Resources Division.

### Purification of six-His-tagged Aga and Ngly2 from HeLa cells

HeLa cells cultured in 10 cm dishes were harvested at 24 h after the transfection of vector expressing Aga tagged with 6×His (Aga-His) or Ngly2 tagged with 6×His (Ngly2-His). C-terminal 6×His tagging of Aga and Ngly2 was carried out by inverse PCR using specific primers 5′-TCATCATCATTAAGCGGCCGCTC-3′ and 5′-TGATGATGAAAGCAGTTCACTGTATGCAA-3′, and 5′-ATTGAGCGGCCGCTCGAGTC-3′ and 5′-GATGATGATGATGATGTTGATAAAACACTAGAT-3′, respectively. The cell pellets were lysed by lysis buffer (50 mM Tris–HCl [pH 7.5], 1 mM EDTA [pH 8.0], 1× cOmplete protease inhibitor cocktail [Roche; catalog no.: 11873580001], 1 mM Pefabloc [Roche; catalog no.: 11429868001], and 1% [w/v] Triton X-100). The cell extract was sonicated and centrifuged at 20,000*g* for 10 min at 4 °C. The supernatants were incubated with a Ni Sepharose 6 Fast Flow histidine-tagged protein purification resin (Cytiva; catalog no.: GE17-5318-06) with end-over-end rotation for 1 h at 4 °C. The Aga-His- or Ngly2-His-bound resins were washed three times with wash buffer (50 mM Tris–HCl [pH 7.5], 200 mM NaCl, and 1× cOmplete protease inhibitor cocktail) containing 10 mM imidazole, and the purified proteins were eluted with elution buffer (wash buffer containing 500 mM imidazole). Eluted sample containing Aga-His or Ngly2-His was ultrafiltrated by Amicon Ultra 0.5 ml (molecular cutoff: 10 kDa; Millipore; catalog no.: UFC5010), concentrated, and the buffer was exchanged with wash buffer.

### Immunoblotting

Heat-denatured samples were loaded and separated on SDS-PAGE gels. Protein samples were transferred to a polyvinylidene difluoride membrane. The membrane was blocked with 5% skim milk in Tris-buffered saline containing 0.05% Tween-20 and incubated with rabbit anti-His-tag antibody (1/1000 dilution, Cell Signaling Technology; catalog no.: 12698). After incubation with goat anti-rabbit IgG secondary antibody conjugated with horseradish peroxidase (1/5000 dilution, Cell Signaling Technology; catalog no.: 7074), target protein bands were detected by Immobilon Western chemiluminescent horseradish peroxidase substrate (Millipore; catalog no.: WBKLS0500). Images were captured using FUSION-SOLO.7S.EDGE (M&S Instruments).

### Activity assays for AGA or PNGase

For the detection of AGA or PNGase activity, sialoglycosylated asparagine [NeuAcα2-6Galβ1-4GlcNAcβ1-2Manα1-3(NeuAcα2-6Galβ1-4GlcNAcβ1-2Manα1-6)Manβ1-4GlcNAcβ1-4GlcNAcβ1-Asn], which contains biantennary glycan with sialic acid at the nonreducing end, or sialoglycosylated hexapeptide [Lys-Val-Ala-Asn(CHO)-Lys-Thr; CHO=NeuAcα2-6Galβ1-4GlcNAcβ1-2Manα1-3(NeuAcα2-6Galβ1-4GlcNAcβ1-2Manα1-6)Manβ1-4GlcNAcβ1-4GlcNAcβ1-] was used, respectively. These glycosylasparagine/glycopeptides were prepared by GlyTech, Inc. An aliquot (2 μl) of purified Aga-His or Ngly2-His was used as an enzymatic solution. Enzymatic reaction was conducted with 50 μl of 50 mM sodium citrate buffer (pH 3.5) or potassium phosphate buffer (pH 6.0), 1× cOmplete protease inhibitor cocktail, and 200 pmol substrates for 6 h at 37 °C (AGA assay) or for 1 h at 25 °C (PNGase assay), respectively. The reaction was terminated by adding 100 μl of 100% EtOH, followed by centrifugation at 20,000*g* for 10 min. The supernatant thus obtained was evaporated to dryness. The resulting pellet was dissolved in water and desalted using a PD-10 column (GE Healthcare; catalog no.: 17085101), in accordance with the manufacturer's protocol. The desalted samples, including free glycan released by the enzymatic reaction, were dried and subjected to labeling with 2-aminopyridine for HPLC analysis, as described previously (72, 73). 2-Aminopyridine-labeled glycans generated by enzymatic reaction were separated by anion-exchange HPLC with a TSKgel DEAE-5PW column (7.5 ϕ × 75 mm; Tosoh Bioscience). The following elution conditions were used: eluent A, 10% acetonitrile (ACN) containing 0.01% triethylamine; eluent B, 10% ACN with 7.4% triethylamine and 3% acetic acid at a flow rate of 1 ml/min at 25 °C. The gradient program was as follows (indicated as % of eluent B): 0 to 5 min, isocratic 0%; 5 to 45 min, 0–20%; 45 to 50 min, isocratic 100%; and 50 to 60 min, isocratic 0%. Fluorescence was detected at an emission wavelength of 380 nm with an excitation wavelength of 310 nm.

### Preparation of recombinant proteins using insect cell-baculovirus system

The DNA constructs were prepared by PCR and In-Fusion reaction using the specific primer pair 5′-AGGACCAGCGGAATTCAAAACCAGGCCCAGCGA-3′ and 5′-ACTTCTCGACAAGCTTTCATTGATAAAACACTAGATAG-3′. The DNA region corresponding to the zebrafish Ngly2 (amino acids: 45–663) was cloned into the expression vector pFastBacAH1, which is based on pFastBac1 (Gibco; catalog no.: 10360014), containing a signal sequence of the baculovirus envelope glycoprotein GP64, a HAT (His) tag, and a Strep-Tag II at the N terminus. The detailed plasmid map is shown in Figure S14. Point mutants (D421A, W480, and E555A) were generated by inverse PCR with the specific primers 5′-CCCAATGAGCACACGTCTCATC-3′ and 5′-CTCACACAGTGCAGCTGTTCG-3′ for *ngly2* (D421A), 5′-CCAGCGGCAATGCCATGGATGACTACTCTC-3′ and 5′-CGCAGTCTTCATGGTGAAGGAGCATTTCTTGT-3′ for *ngly2* (W480A), and 5′-CATTTTGTGTGACGTCGCATTACTT-3′ and 5′-CCCCACAGTTGTTGTCATCGG-3′ for *ngly2* (E555A). The cloning region follows a human rhinovirus 3C protease cleavage site, and thus those tags are removable at subsequent purification steps. The constructed plasmids for Ngly2 (amino acids: 45–663) and its mutants were inserted into the EMBacY bacmid genome by Tn7 transposition in the DH10BacY strain (Geneva Biotech; catalog no.: EMBacY). Transfection of bacmid into Sf9 cells was performed using FuGENE HD transfection reagent, basically in accordance with the Bac-to-Bac manuals (Gibco; catalog no.: 10359016). The Ngly2 protein was secreted in the culture medium. The pellet was removed from the medium by centrifugation at 1200*g* for 5 min at 25 °C, and the recovered supernatant was loaded onto a 0.45 μm disk filter (Millipore; catalog no.: SLHP033RB), and the filtrate was immediately subjected to purification, as described later.

For the purification of proteins, the supernatant was directly applied to a HisTrap HP column (Cytiva; catalog no.: 17524802), and samples were eluted with a linear gradient of 20 to 500 mM imidazole buffer containing 20 mM Tris–HCl (pH 8.0) and 500 mM NaCl. The peak fractions were collected, then loaded onto a StrepTrap HP column (Cytiva; catalog no.: 28907548) and manually eluted using 3 mM desthiobiotin buffer containing 50 mM Tris–HCl (pH 7.5) and 250 mM NaCl. In cases requiring further purification, N-terminal tags were removed by 3C protease during overnight dialysis at 4 °C. The sample was then concentrated using Amicon Ultra Centrifugal Filter 4 ml (50 kDa molecular weight cutoff; Millipore, UFC805024) and subjected to size-exclusion chromatography using a Superdex200 increase 10/300 GL column (Cytiva; catalog no.: 28990944) equilibrated in 20 mM Tris–HCl (pH 8.0) and 150 mM NaCl. The enzyme active fractions were collected and used for subsequent enzymatic assays.

### PNGase activity assay using 5-FAM-labeled glycosylated hexapeptide

For the enzymatic characterization of Ngly2, 5-FAM-labeled glycosylated hexapeptide was synthesized by GlyTech, Inc. 5-FAM GH possesses a single asialobiantennary glycan. PNGase activity was assayed using a 50 μl reaction mixture containing 200 pmol 5-FAM GH, 50 mM sodium citrate buffer (pH 3.5), and 0.15 μg of enzymatic solution. The reaction mixtures were incubated at 30 °C for 30 min unless noted otherwise, and the reaction was terminated by adding 100 μl of 100% EtOH. After centrifugation at 20,000*g* for 10 min, the resulting supernatant was evaporated to dryness and then subjected to HPLC analysis using InertSustain C18 HP (3 μm, 3.0 × 150 mm; GL Science, catalog no.: 5020-14425). For the separation of 5-FAM GH, the following HPLC conditions were applied: eluent A, distilled water containing 0.1% TFA; eluent B, 100% ACN containing 0.1% TFA. The column temperature was set to 40 °C. The column was equilibrated with eluent A/eluent B (90/10) at a flow rate of 0.45 ml/min. After injecting a sample, the concentration of eluent B was increased linearly from 10% to 50% over 20 min 5-FAM GH, and its reaction products were detected by measuring fluorescence (excitation 494 nm, emission 518 nm). Optimal Ph was investigated with an appropriate buffer (50 Mm glycine hydrochloride buffer for Ph 2.0–3.0, 50 Mm sodium citrate buffer for Ph 3.0–6.0, and 50 mM potassium phosphate buffer for pH 6.0–7.0). The effect of enzymatic products on the enzymatic activity of Ngly2 was determined by incubation with the following compounds: 100 μM or 10 μM asialobiantennary glycan (Glycan), purchased from Fushimi Pharmaceutical Ltd (catalog no.: 171817), Asn-peptide: Lys-Val-Ala-Asn-Lys-Thr, or Asp-peptide: Lys-Val-Ala-Asp-Lys-Thr. Asn- and Asp-peptides were chemically synthesized by the Support Unit for Bio-Material Analysis in RIKEN CBS, Research Resources Division.

### Preparation of the pentapeptide (Ser-Arg-Asn-Leu-Thr) with different glycan structures

All solvents and the various reagents were purchased from Kanto Chemical Co, Inc, Tokyo Chemical Industry, and Wako Pure Chemical Industries Ltd and were used without further purification. Amino acid derivatives and reagents for peptide synthesis were purchased from Watanabe Chemical and AAPPTEC. Reversed-phase flash column chromatography was performed on a Combi Flash Rf200 (TELEDYNE ISCO). HPLC was performed on a Prominence HPLC system equipped with a fluorescence detector RF-20Axs and photodiode array detector (Shimadzu) or a preparative HPLC system equipped with a UV detector UV/Vis-156 (Gilson). Microwave-assisted peptide synthesis was performed on a Biotage initiator + synthesizer. MALDI-TOF MS was recorded in high-resolution mode with positive ion mode on an AXIMA Performance (Shimadzu). NMR spectra were recorded with JNM-ECA600 (^1^H: 600 MHz; JEOL). Chemical shifts are given in parts per million and referenced to residual internal HOD (partially deuterated water; δH 4.67 in D_2_O).

### *N*-(9-Fluorenylmethyloxycarbonyl)-*N*-{α-D-mannopyranosyl-(1 → 3)-[α-D-mannopyranosyl-(1 → 6)]-β-D-mannopyranosyl-(1 → 4)-2-acetamido-2-deoxy-β-D-glucopyranosyl-(1 → 4)-2-acetamido-2-deoxy-β-D-glucopyranosyl}-L-asparagine (Fmoc-Asn(Man_3_GlcNAc_2_)-OH)

To a solution of Man_3_GlcNAc_2_ (5.0 mg, 4.0 μmol) in H_2_O was added saturated aqueous NH_4_HCO_3_ (4.8 ml) and then stirred at 40 °C for 13 h. The reaction mixture was concentrated *in vacuo* three times and lyophilized. The residue was dissolved in dimethyl sulfoxide (DMSO) (370 μl), and then Fmoc-Asp(OPfp)-O^*t*^Bu (20 mg, 34.4 μmol), *N*,*N*-diisopropylethylamine (DIPEA) (7.1 μl, 37.9 μmol) were added. The reaction mixture was stirred at room temperature under an Ar atmosphere for 23 h. The reaction mixture was diluted with water, and the aqueous layer was washed with EtOAc. The aqueous layer was purified by reversed-phase flash column chromatography (H_2_O/CH_3_CN = 100/0 to 50/50) to afford glycosyl asparagine derivative. A solution of glycosyl asparagine derivative in TFA (600 μl) was stirred at room temperature for 30 min. Then, the reaction mixture was diluted with CH_2_Cl_2_ and dried up by nitrogen (N_2_) gas. The residue was purified by HPLC using a TOSOH TSKgel ODS80Ts column (H_2_O in 0.1% TFA/CH_3_CN in 0.1% TFA = 100/0 to 50/50) to afford Fmoc-Asn(Man_3_GlcNAc_2_)-OH (Fig. S15*A*) (4.5 mg, 3.6 μmol, 61%); ^1^H-NMR (600 MHz, D_2_O) δ 7.77 (d, *J* = 7.6 Hz, 2H, Fmoc-*H*), 7.56 (d, *J* = 7.2 Hz, 2H, Fmoc-*H*), 7.36 (t, *J* = 7.4 Hz, 2H, Fmoc-*H*), 7.30-7.27 (m, 2H, Fmoc-*H*), 4.96 (s, 1H), 4.87 (d, *J* = 9.6 Hz, 1H), 4.77 (s, 1H), 4.64 (s, 1H), 4.48-4.41 (m, 2H), 4.28-4.26 (m, 1H), 4.20-4.19 (m, 1H), 4.12 (s, 1H), 3.92 (s, 1H), 3.83 (s, 1H), 3.79-3.37 (m, 28H), 2.62-2.52 (m, 2H, -C*H*_*2*_Asn), 1.93 (s, 3H, -COC*H*_*3*_), 1.76 (s, 3H, -COC*H*_*3*_) (Fig. S16*A*); MALDI-TOF MS *m/z*: [M+Na]+ Calcd for C_46_H_78_NaN_2_O_36_ 1269.43; found 1271.33.

### *N*-(9-Fluorenylmethyloxycarbonyl)-*N*-[[α-D-mannopyranosyl-(1 → 3)]-[α-D-mannopyranosyl-(1 → 6)]-β-D-galactopyranosyl-(1 → 4)-2-acetamido-2-deoxy-β-D-glucopyranosyl-(1 → 4)-[α-L-fucosyl-(1 → 6)]-2-acetamido-2-deoxy-β-D-glucopyranosyl)]-L-asparagine (Fmoc-Asn(Man_3_GlcNAc(Fuc)GlcNAc)-OH)

To a solution of Man_3_GlcNAc(Fuc)GlcNAc (8.1 mg, 7.7 μmol) in H_2_O was added saturated aqueous NH_4_HCO_3_ (3 ml) and then stirred at 40 °C for 24 h. The reaction mixture was concentrated *in vacuo* three times and lyophilized. The residue was dissolved in DMSO (400 μl), and then Fmoc-Asp(OPfp)-O^*t*^Bu (47 mg, 81 μmol) and DIPEA (17 μl, 87 μmol) were added. The reaction mixture was stirred at 40 °C under an Ar atmosphere for 24 h. The reaction mixture was diluted with water, and the aqueous layer was washed with EtOAc. The aqueous layer was purified by reversed-phase flash column chromatography (H_2_O/CH_3_CN = 100/0 to 60/40) to afford glycosyl asparagine derivative. A solution of glycosyl asparagine derivative in TFA (500 μl) was stirred at room temperature for 30 min. The reaction mixture was diluted with CH_2_Cl_2_ and dried up by N_2_ gas. The residue was purified by HPLC using a TOSOH TSKgel ODS80Ts column (H_2_O in 0.1% TFA/CH_3_CN in 0.1% TFA = 76/24) to afford Fmoc-Asn(Man_3_GlcNAc(Fuc)GlcNAc)-OH (Fig. S15*B*) (2.7 mg, 1.96 μmol, 25%); ^1^H-NMR (600 MHz, D_2_O) δ 7.77 (d, *J* = 6.2 Hz, 2H, Fmoc-*H*), 7.56-7.54 (m, 2H, Fmoc-*H*), 7.36 (t, *J* = 7.6 Hz, 2H, Fmoc-*H*), 7.29 (q, *J* = 7.7 Hz, 2H, Fmoc-*H*), 4.96 (s, 1H), 4.89 (d, *J* = 9.6 Hz, 1H), 4.77 (s, 1H), 4.64 (s, 1H), 4.55-4.51 (m, 1H), 4.46-4.44 (m, 1H), 4.39-4.36 (m, 1H), 4.20 (t, *J* = 6.7 Hz, 2H), 4.12 (s, 1H), 3.92 (s, 1H), 3.83-3.40 (m, 33H), 2.58-2.51 (m, 2H, -C*H*_*2*_Asn), 1.95 (s, 3H, -COC*H*_*3*_), 1.77 (bs, 3H, -COC*H*_*3*_), 0.89 (d, *J* = 6.2 Hz, 3H, -C*H*_*3*_) (Fig. S16*B*); MALDI-TOF MS *m/z*: [M+Na]+ Calcd for C_59_H_84_NaN_3_O_34_ 1415.49; found 1415.67.

### *N*-(9-Fluorenylmethyloxycarbonyl)-*N*-[[α-D-mannopyranosyl-(1 → 2)-α-D-mannopyranosyl-(1 → 2)-α-D-mannopyranosyl-(1 → 3)]-[α-D-mannopyranosyl-(1 → 6)]-β-D-mannopyranosyl-(1 → 4)-2-acetamido-2-deoxy-β-D-glucopyranosyl-(1 → 4)-2-acetamido-2-deoxy-β-D-glucopyranosyl)]-L-asparagine (Fmoc-Asn(Man_5_GlcNAc_2_)-OH)

To a solution of Man_5_GlcNAc_2_ (10.0 mg, 8.1 μmol) in H_2_O was added saturated aqueous NH_4_HCO_3_ (3 ml) and then stirred at 40 °C for 11 h. The reaction mixture was concentrated *in vacuo* three times and lyophilized. The residue was dissolved in DMSO (265 μl), and then Fmoc-Asp(Opfp)-O^*t*^Bu (28 mg, 48 μmol) and DIPEA (10 μl, 53 μmol) were added. The reaction mixture was stirred at 40 °C under an Ar atmosphere for 14 h. The reaction mixture was diluted with water, and the aqueous layer was washed with EtOAc. The aqueous layer was purified by reversed-phase flash column chromatography (H_2_O/CH_3_CN = 100/0 to 50/50) to afford glycosyl asparagine derivative. A solution of glycosyl asparagine derivative in TFA (500 μl) was stirred at room temperature for 30 min. The reaction mixture was diluted with CH_2_Cl_2_ and dried up by N_2_ gas. The residue was purified by HPLC using a TOSOH TSKgel ODS80Ts column (H_2_O in 0.1% TFA/CH_3_CN in 0.1% TFA = 74/26) to afford Fmoc-Asn(Man_5_GlcNAc_2_)-OH (Fig. S15*C*) (2.6 mg, 1.7 μmol, 21%); ^1^H-NMR (600 MHz, D_2_O) δ 7.77 (d, *J* = 7.6 Hz, 2H, Fmoc-*H*), 7.56 (d, *J* = 7.2 Hz, 2H, Fmoc-*H*), 7.36 (t, *J* = 7.4 Hz, 2H, Fmoc-*H*), 7.30-7.28 (m, 2H, Fmoc-*H*), 5.20 (s, 1H), 5.16 (s, 1H), 4.90 (s, 1H), 4.87 (d, *J* = 10.0 Hz, 1H), 4.77 (s, 1H), 4.63 (d, *J* = 6.9 Hz, 1H,), 4.49-4.43 (m, 2H), 4.29 (t, *J* = 6.0 Hz, 1H), 4.20 (s, 1H), 4.09 (s, 1H), 3.96-3.92 (m, 3H), 3.86-3.38 (m, 40H), 2.57-2.53 (m, 1H, -C*H*_*2*_Asn), 1.93 (s, 3H, -COC*H*_*3*_), 1.76 (bs, 3H, -COC*H*_*3*_) (Fig. S16*C*); MALDI-TOF MS *m/z*: [M+Na]+ Calcd for C_65_H_94_NaN_4_O_40_ 1593.53; found 1593.89.

### *N*-(9-Fluorenylmethyloxycarbonyl)-*N*-[[α-D-mannopyranosyl-(1 → 2)-α-D-mannopyranosyl-(1 → 2)-α-D-mannopyranosyl-(1 → 3)]-[[α-D-mannopyranosyl-(1 → 3)]-[α-D-mannopyranosyl-(1 → 2)-α-D-mannopyranosyl-(1 → 2)-α-D-mannopyranosyl-(1 → 6)]]-β-D-mannopyranosyl-(1 → 4)-2-deoxy-2-acetamido-β-D-glucopyranosyl-(1 → 4)-2-deoxy-2-acetamido-D-glucopyranosyl]-L-asparagine (Fmoc-Asn(Man_8_GlcNAc_2_)-OH)

To a solution of Man_8_GlcNAc_2_ (5.5 mg, 3.2 μmol) in H_2_O was added saturated aqueous NH_4_HCO_3_ (3 ml) and then stirred at 40 °C for 21 h. The reaction mixture was concentrated *in vacuo* three times and lyophilized. The residue was dissolved in DMSO (265 μl), and then Fmoc-Asp(OPfp)-O^*t*^Bu (11 mg, 19 μmol) and DIPEA (4.0 μl, 21 μmol) were added. The reaction mixture was stirred at room temperature under an Ar atmosphere for 24 h. The reaction mixture was diluted with water, and the aqueous layer was washed with EtOAc. The aqueous layer was purified by reversed-phase flash column chromatography (H_2_O/CH_3_CN = 100/0 to 60/40) to afford glycosyl asparagine derivative. A solution of glycosyl asparagine derivative in TFA (200 μl) was stirred at room temperature for 30 min. The reaction mixture was diluted with CH_2_Cl_2_ and dried up by N_2_ gas. The residue was purified by HPLC using a TOSOH TSKgel ODS80Ts column (H_2_O in 0.1% TFA/CH_3_CN in 0.1% TFA = 75/25) to afford Fmoc-Asn(Man_8_GlcNAc_2_)-OH (Fig. S15*D*) (3.3 mg, 1.6 μmol, 50%); ^1^H-NMR (600 MHz, D_2_O) δ 7.78 (d, *J* = 7.2 Hz, 2H, Fmoc-*H*), 7.57 (d, *J* = 7.2 Hz, 2H, Fmoc-*H*), 7.37 (t, *J* = 7.2 Hz, 2H, Fmoc-*H*), 7.31-7.28 (m, 2H, Fmoc-*H*), 5.21 (s, 1H), 5.18 (s, 1H), 5.02 (s, 1H), 4.95 (s, 1H), 4.91 (s, 2H), 4.88 (d, *J* = 9.3 Hz, 1H), 4.73 (d, *J* = 7.9 Hz, 1H), 4.64 (s, 1H), 4.50-4.46 (m, 2H), 4.33 (t, *J* = 6.5 Hz, 1H), 4.21 (d, *J* = 5.5 Hz, 1H), 4.10 (d, *J* = 2.4 Hz, 1H), 4.01-3.39 (m, 59H), 2.57 (m, 1H, -C*H*_*2*_Asn), 1.93 (d, 3H, -COC*H*_*3*_), 1.77 (bs, 3H, -COC*H*_*3*_) (Fig. S16*D*); MALDI-TOF MS *m/z*: [M+Na]+ Calcd for C_83_H_124_NaN_4_O_55_ 2079.69; found 2081.30.

### Synthesis of glycopeptides

Glycopeptides were synthesized by the Fmoc solid-phase method on a Rink amide PEG resin XV (0.21 mmol/g) with 1-[bis(dimethylamino)methylene]-1*H*-1,2,3-triazolo[4,5-*b*]pyridinium 3-oxide hexafluorophosphate (HATU) as a coupling reagent. Side-chain protections for amino acids were as follows: Ser(^*t*^Bu), Arg(Pbf), Asp(^*t*^Bu), and Thr(^*t*^Bu). The Fmoc group was removed with 20% piperidine in *N*-methyl-2-pyrrolidone (NMP) at room temperature (1 min ×1, 15 min ×1). The coupling conditions of Fmoc amino acids, except for Fmoc-Asn(glycan)-OH, were as follows: Fmoc-AA-OH (3 equivalents [eq.]), HATU (3 eq.), and DIPEA (6 eq.) in NMP stirred at room temperature for 45 min. Fmoc-Asn(glycan)-OH (1 eq.), (7-azabenzotriazol-1-yloxy)tripyrrolidinophosphonium hexafluorophosphate (4 eq.), 1-hydroxy-7-azabenzotriazole(4 eq.), and collidine (5 eq.) in NMP were used under microwave heating at 60 °C for 60 min. After the introduction of a MANT group using *N*-methylanthranilic acid (5 eq.), HATU (5 eq.), and DIPEA (10 eq.) at the N terminus, the peptidyl resin was washed with NMP and dichloromethane and then dried *in vacuo*. The peptidyl resin was treated with 2.5% triisopropylsilane and 2.5% water in TFA for 2 h at room temperature. The resin was removed by filtration, and the filtrate was added into Et_2_O to precipitate the peptide. The solid was then washed with Et_2_O and dried *in vacuo*. The crude peptides were purified by HPLC using a TOSOH TSKgel ODS80Ts column (H_2_O in 0.1% TFA/CH_3_CN in 0.1% TFA = 86/14) to afford GlcNAc_2_-peptide (1.68 mg, 1.49 μmol, 14%). The glycopeptides were identified by electrospray ionization–MS: MANT-Ser-Arg-Asn(GlcNAc_2_)-Leu-Thr, *m/z*: [M+H]^+^ Calcd for C_47_H_78_N_13_O_19_ 1128.55; found 1128.43 (Fig. S16*E*); MANT-Ser-Arg-Asn(Man_3_GlcNAc_2_)-Leu-Thr (270 μg, 0.17 μmol, 7%), *m/z*: [M+H]^+^ Calcd for C_65_H_108_N_13_O_34_ 1614.71; found 1614.68, [M+2H]^2+^ Calcd for C_65_H_109_N_13_O_34_ 807.86; found 808.09 (Fig. S16*F*); MANT-Ser-Arg-Asn(Man_3_GlcNAc(Fuc)GlcNAc)-Leu-Thr (1.54 μg, 0.0009 μmol, 0.04%), *m/z*: [M+H]^+^ Calcd for C_71_H_118_N_13_O_38_ 1760.77; found 1760.69, [M+2H]^2+^ Calcd for C_71_H_119_N_13_O_38_ 880.89; found 881.18 (Fig. S16*G*); MANT-Ser-Arg-Asn(Man_5_GlcNAc_2_)-Leu-Thr (370 μg, 0.19 μmol, 10%), *m/z*: [M+H]^+^ Calcd for C_77_H_128_N_13_O_44_ 1939.82; found 1939.43, [M+2H]^2+^ Calcd for C_77_H_129_N_13_O_44_ 969.91; found 970.21 (Fig. S16*H*); and MANT-Ser-Arg-Asn(Man_8_GlcNAc_2_)-Leu-Thr (5.93 μg, 0.00246 μmol, 0.1%), *m/z*: [M+2H]^2+^ Calcd for C_95_H_159_N_13_O_59_ 1213.49; found 1213.36 (Fig. S16*I*).

### Substrate specificity of Ngly2

Enzymatic reaction for Ngly2 was performed in 50 μl of 50 mM sodium citrate buffer (pH 4.0), 5 μM substrate, and 0.3 μg of recombinant protein, with incubation at 30 °C for 30 min. The reaction mixture was subjected to HPLC analysis using a TSKgel ODS80Ts column. For the product analysis, the following HPLC conditions were applied: eluent A, distilled water containing 0.1% TFA and eluent B, ACN containing 0.1% TFA. The column temperature was set to 40 °C. The column was equilibrated with eluent A/eluent B (86/14) at a flow rate of 1 ml/min. After injecting a sample, the concentration of eluent B was increased linearly from 14% to 24% over 7 min. The original substrate, namely, glycosylated pentapeptide, and the deglycosylated product were detected by measuring fluorescence (excitation 340 nm, emission 440 nm).

### Cryo-EM data acquisition

We achieved the best ice quality and particle dispersion using copper R2/1 QuantiFoil grids (QuantiFoil GmbH), which were glow-discharged with a PIB-10 (Vacuum Device Co) for 30 s in air. After glow discharge, 2.5 μl of 1.1 mg/ml Ngly2 sample (20 mM Tris–HCl [pH 8.0], 150 mM NaCl) was applied before blotting for 3 s with a blot force of five using a Vitrobot Mark IV (Thermo Fisher Scientific) and immediately plunged into liquid ethane.

Data were acquired on a Thermo Fisher Scientific Titan Krios G4 (Thermo Fisher Scientific) equipped with a cold-FEG, Selectris-X energy filter, and Falcon 4 direct detector at a magnification of 215,000×, equivalent to 0.57 Å/pixel on specimen. Total electron dose per image was 50 e^−^/Å^2^ with an exposure time of 1.83 s, and eight images were collected simultaneously with a hole. Aberration-free beam image shift acquisition was used as implemented in the EPU acquisition software with a target defocus range of between 0.8 and 1.6 μm underfocus.

### Cryo-EM data processing

CryoSPARC (74) version 4.4.1, was used for all image processings, as summarized in Figure S17. Exposures were streamed directly to CryoSPARC Live where patch motion correction was carried out with electron-event representation movies fractionated into 50 frames (1e^−^/Å^2^/frame) with an upsampling of 1 (for 4096 × 4096 micrographs), which were saved in 16 bit mrc file. Micrographs with a poor contrast transfer function (CTF) fit estimate were removed. The first 547 processed micrographs were exported from CryoSPARC Live into the main CryoSPARC environment, and a subset of 30 micrographs were selected manually. These particles were 2D classified into 10 classes, and two classes consisting of most of the selected particles were used for template picking. After adjusting the local power scores and normalized cross-correlation scores to select only clear particles, 19,085 particles were extracted into a 512 × 512 pixel box with downsampling to 128 × 128 pixel boxes (equivalent to 2.28 Å/pixel) before classification into 50 classes. The default parameters were used, except for “Initial classification uncertainty factor” set to 5, “Number of final full iterations” set to 2, and “Number of online-EM iterations” set to 30, with two graphics processing units used. The four clearest, unique classes were selected and used to pick a larger exported subset of micrographs comprising 1062 micrographs, where 24,949 particles were extracted using the same parameters. Two-dimensional classification into 80 classes (other parameters remaining the same except for the addition of a 150 Å circular mask) permitted the selection of 75 classes, comprising 23,467 particles. These particles were used to generate six exploratory *ab initio* maps (other parameters having the default settings), which immediately proceeded to heterogeneous reconstruction (with the default parameters except that “Window inner radius” was set to 0.5). Nonuniform (NU) refinement (75) of the class most closely matching the 2D projections resulted in an 8 Å map. Further 2D classification of the previous 75 classes into 30 classes (other parameters remaining the same as applied previously) permitted the selection of seven unique classes, which were used to pick 3567 micrographs for a total of 143,628 particles. These were then extracted into smaller 450-pixel boxes downsampled to 150-pixel boxes. These particles were extracted and 2D classified as before, and the four highest-resolution classes were selected for *C*1 NU refinement. Here, it became clear that the complex was a symmetric dimer, and thus heterogeneous refinement of the extract particle set was carried out with one “good” target and two “junk” classes with *C*2 symmetry using the previous *C*1 NU refined map aligned to symmetry. This resulted in 74,546 particles assigned to the “good” target class, which achieved 4.2 Å resolution with NU refinement. An exploratory symmetry expansion and 3D classification (CryoSPARC 3D classification is the closest equivalent to “alignment disabled” 3D classification in RELION) revealed no structural heterogeneity, and thus further work remained with *C*2 symmetry imposed. The full dataset was exported from CryoSPARC Live, and template picked using the four clearest unique classes from above, which were extracted into 450 pixel boxes downsampled to 150 pixel boxes for a total of 2,187,466 particles. These particles were 2D classified into 300 classes with the default parameters apart from the following: “Window inner radius,” 0.7; “Initial classification uncertainty factor,” 8; “Number of final full iterations,” 2; “Number of online-EM iterations,” 100; and “Batchsize per class,” 300, using two graphics processing units. A total of 281 classes were selected, comprising 2,047,370 particles, which then underwent heterogeneous refinement into six classes. The highest-resolution class, containing 579,111 particles, then proceeded to NU refinement, where it reached the (downsampled) sampling limit. After re-extracting with 1.5× downsampling and recentering on refined shifts, 572,828 particles remained as CryoSPARC automatically removes any particles where the box clips the edge of the micrograph. The beam image shifts were imported from the EPU .xml files, and the particles and micrographs were assigned to each of the beam shift groups. After global CTF refinement to correct for beam tilt, trefoil, and magnification anisotropy and local CTF refinement to optimize particle defocus, 2.8 Å resolution was achieved. After reference-based motion correction (default parameters), the resolution improved almost imperceptibly. Experimental 2D classification was carried out, and the 44 highest-resolution classes comprising 371,054 particles were selected and then downsampled back to 300-pixel boxes. Afterward, another round of global CTF refinement (beam tilt and magnification anisotropy only) and local CTF refinement were performed, before local refinement with NU refinement, being enabled the production of a 2.7 Å map. Local resolution was estimated using the CryoSPARC implementation of the blocres algorithm from Bsoft (76) before filtering the map by local resolution.

### Model building and validation

An initial model of Ngly2 dimer was constructed by AlphaFold-Multimer (doi.org/10.1101/2021.10.04.463034) and manually fitted to the final density map using Coot (developed by Paul Emsley and Kevin D. Cowtan) (77). Several prominent densities were identified near asparagine residues, which corresponded to *N*-glycosylation. *N*-Acetylglucosamine and fucose were built depending on each density size. The model of the entire structure was iteratively refined using “phenix.real_space_refine” of the Phenix package (78), with secondary structure restraints. Model validation for stereochemistry was performed in MolProbity (79) (Table S2). Molecular graphics and density maps were prepared with PyMOL (https://pymol.org/2/) and UCSF ChimeraX (80).

### *In silico* modeling of Ngly2 and glycopeptide complex using AlphaFold3

The structure of the Ngly2 in complex with glycopeptide (Lys-Val-Ala-Asn(CHO)-Lys-Thr; CHO = GlcNAcMan(GlcNAcMan)ManGlcNAcGlcNAcβ1-) was predicted by AlphaFold3 (48). The structural map predicted by AlphaFold3 was plotted in PyMOL (Schrödinger, Inc).

### Quantitative real-time PCR

The mRNA expression levels of various genes were analyzed using cDNAs generated from zebrafish tissues, embryos, and larval fish at different developmental stages. Real-time PCR was conducted using PowerUP SYBR Green Master Mix for qPCR (Applied Biosystems; catalog no.: A25742) with the specific primers 5′-GATGTTGGCGATGGATGTGC-3′ and 5′-CAGGCCTCCCCTTTTGTAGG-3′ for *ngly2* and 5′-AGCACCCTGTGCTGCTCACT-3′ and 5′-CGCCATACAGAGCAGAAGCC-3′ for *actb* using QuantStudio 5 Real-Time PCR System (Applied Biosystems). The expression levels of the target genes were normalized to the corresponding *actb* mRNA expression level.

### Indirect immunofluorescence

To determine the subcellular localization of Ngly2, C-terminal HA tagging of Ngly2 was performed by PCR using specific primers 5′-CAGATTACGCTTGAGCGGCC-3′ and 5′-GAACATCGTATGGGTATTGATAAAACACTAG-3′. Immunofluorescent staining was performed in *ngly2*-transfected BRF41 cells cultured on glass coverslips. Cells were fixed with 4% paraformaldehyde/PBS and then permeabilized with 0.1% Triton X-100–PBS. Permeabilized cells were blocked with 1% bovine serum albumin. Blocked cells were incubated with anti-HA mouse monoclonal antibody (1/200 dilution, H9658; Sigma–Aldrich) and anti-Lamp1 rabbit polyclonal antibody (1/200 dilution; ab24170; Abcam), followed by incubation with Alexa Fluor 488 goat anti-mouse IgG (H + L) (1/500 dilution, A11029; Invitrogen) and Alexa Fluor 555 goat anti-rabbit IgG (H + L) (1/500 dilution, A21429; Invitrogen). The coverslips were mounted on a glass slide and observed using a confocal laser scanning microscope (FV3000; Olympus).

### Establishment of Ngly2-KO zebrafish using CRISPR–Cas9

sgRNAs for zebrafish *ngly2* were designed using CRISPRdirect (https://crispr.dbcls.jp) (50). The synthetic sgRNAs (*ngly2* gRNA 1 for 68–90: GUCGUUUCCUUGAGAAGACGguuuuagagcuagaaauagcaaguuaaaauaaggcuaguccguuaucaacuugaaaaaguggcaccgagucggugcuuuu and *ngly2* gRNA 2 for 139–161: GGCCCAGCGAUGCUCUUGGguuuuagagcuagaaauagcaaguuaaaauaaggcuaguccguuaucaacuugaaaaaguggcaccgagucggugcuuuu) were obtained from FASMAC. The two sgRNAs (25 ng/μl) with 400 ng/μl Cas9 Nuclease protein NLS (NIPPON GENE, catalog no.: 316-08651) were microinjected into one-cell-stage zebrafish embryos (F_0_ founder) derived from RW zebrafish. For the genotyping, genomic DNA was extracted from zebrafish tail fin. Genomic fragments at the target sites for the sgRNAs were amplified by PCR using OneTaq 2× Master Mix (New England Biolabs; catalog no.: M0482) and specific primers 5′-CAGTGTGGTGGAATTCACACGCCACGTGTTCAC-3′ and 5′-TAGACTCGAGCGGCCTTGTTTTGTCTATTGTAAGACT-3′. The PCR was performed under the following conditions: 30 cycles of 94 °C for 20 s, 60 °C for 20 s, and 68 °C for 1 min. The amplified PCR products were subjected to 2% agarose gel electrophoresis.

The F_0_ generation with mosaic *ngly2* mutation was selected and crossed with RW to obtain the F_1_ generation. The *ngly2* mutation in F_1_ was confirmed by agarose gel electrophoresis of the PCR product around the target site. The amplified PCR products were subcloned into the pcDNA 3.1 plasmid, followed by sequencing. The F_1_ generation having the same mutation pattern in the *ngly2* genome was crossed to establish the F_2_ generation.

### Isolation of free glycans derived from zebrafish-fertilized eggs

The purification of free glycans was carried out essentially based on a previously established method (81). Briefly, fertilized eggs from WT and Ngly2-KO strains were collected after spawning. One hundred twenty milligrams of fertilized eggs were homogenized in an equivalent volume of ethanol using a BioMasher II (Nippi; catalog no.: 893062). After centrifugation at 20,000*g* and 4 °C for 10 min, the resulting supernatant was collected and evaporated to dryness. The dried samples were dissolved in 0.5 ml of distilled water and subjected to desalting using an InsertSepGC column (150 mg/3 ml; GL Science, catalog no.: 5010-68000), as reported previously (81). The free glycan fraction was eluted with 2.5 ml of 40% ACN containing 0.05% TFA and then evaporated to dryness. The dried samples were redissolved in 0.5 ml of distilled water and desalted using a PD-10 column, followed by 2PA labeling. The 2PA-labeled samples were detected by HPLC using a TSKgel DEAE-5PW column, as reported previously (82, 83). The flowthrough fraction was collected as neutral glycans for further structural analyses. To remove sialic acids, the glycan sample was incubated in 50 mM sodium acetate buffer (pH 5.0) with 1 mU α2-3,6,8,9 sialidase (neuraminidase from *Arthrobacter ureafaciens*; Roche, catalog no.: 10269611001) at 37 °C for overnight. To remove sialidase-resistant peaks, the sample was hydrolyzed by 0.1 N TFA at 80 °C for 2 h (84).

The neutral glycan fraction was treated with 1 mg/ml glucoamylase (FUJIFILM Wako Pure Chemical Corporation; catalog no.: 077-04071) in 50 mM sodium acetate buffer (pH 5.0) at 37 °C for overnight. The glucoamylase-treated samples were separated by size fractionation HPLC with an NH2P-40-3E column (3.0 ϕ × 250 mm; Shodex, catalog no.: F7630007). The mobile phase consisted of 93% ACN in 0.3% acetate buffer (adjusted to pH 7.0 by adding ammonia eluent A) and 20% ACN in 0.3% acetate buffer (pH 7.0 adjusted by ammonia; eluent B). The flow rate was 0.40 ml/min at 25 °C. The gradient program was as follows (indicated as % of eluent B): 0 to 0.5 min, isocratic 1%; 0.5 to 3 min, 1 to 24%; 3 to 33 min, 24 to 55%; 33.1 to 35 min, isocratic 70%; 35.1 to 40 min, isocratic 100%; and 40.1 to 50 min, isocratic 1%. Fluorescence was detected at an emission wavelength of 380 nm with an excitation wavelength of 310 nm.

The fractionated neutral glycan fraction was further analyzed using a Shimadzu LCMS-9030 mass spectrometer coupled with a Nexera X3 ultra high-performance liquid chromatograph (Shimadzu) using TSKgel Amide-80 (2.0 ϕ × 150 mm; Tosoh Bioscience; catalog no.: 0021865). The gradient program was as follows (indicated as % of eluent B): eluent A, 80% ACN with 50 mM formic acid/ammonium (pH 4.5) and eluent B, 50 mM formic acid/ammonium (pH 4.5): 0 to 2 min, isocratic 6.3%; 2 to 30 min, 6.35 to 37.5%; 30 to 40 min, 37.5 to 50%; 40 to 42 min, isocratic 50%; and 42.1 to 50 min, isocratic 6.3%, with a flow rate of 0.2 ml/min. The column temperature was set to 45 °C. Fluorescence was detected at an emission wavelength of 380 nm with an excitation wavelength of 310 nm. The mass spectrometer was operated with an electrospray source in positive ionization mode. The electrospray ionization source conditions were as follows: nebulizer gas rate of 2.0 l/min, heating gas rate of 10.0 l/min, desolvation line temperature of 250 °C, heat block temperature of 400 °C, probe voltage of +4.50 kV, and interface temperature of 300 °C. The MS was obtained using 0.1 s event time. Labsolution software was used for instrument operation and data analysis. To investigate the glycan structures in detail, the fractionated samples were subjected to enzymatic digestion (4 U α1-3,4 fucosidase [from *P. dulcis*; New England Biolabs; catalog no.: M0482]; 5 mU β1-3,4 galactosidase [from bovine testis; Agilent; catalog no.: GKX-5013]; 0.05 U β-*N*-acetylhexosaminidase [from *Canavalia ensiformis*; Seikagaku Biobusiness Corporation; catalog no.: 100094]) at 37 °C for overnight, with the buffer provided by the manufacturer. For β-*N*-acetylhexosaminidase, reaction was carried out in 50 mM sodium acetate buffer (pH 5.0).

## Data availability

All data are available in the article and supporting information files.

## Supporting information

This article contains supporting information (70).

## Conflict of interest

The authors declare that they have no conflicts of interest with the contents of this article.
