# Supplementary material for: Structural characterization of zebrafish Ngly2, an ovary-enriched acid PNGase required for egg-free glycan production
Source: J Biol Chem. 2025 Nov 5;301(12):110906. doi: 10.1016/j.jbc.2025.110906 (PMC12753239; doi:10.1016/j.jbc.2025.110906)
Supplement: Supplemental Tables [file mmc1.pptx]

## Slide 1
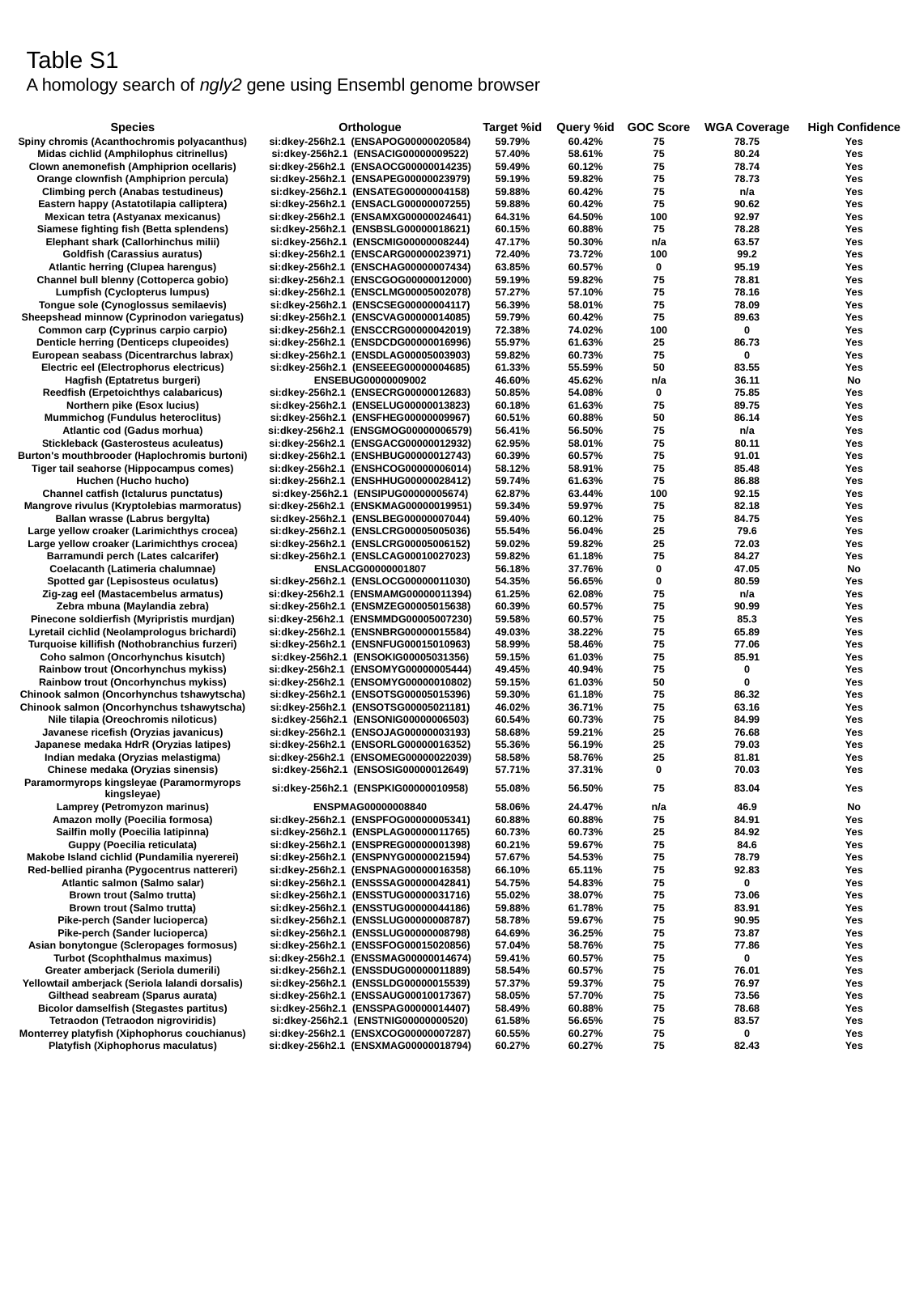

Table S1
A homology search of ngly2 gene using Ensembl genome browser
| Species | Orthologue | Target %id | Query %id | GOC Score | WGA Coverage | High Confidence |
| --- | --- | --- | --- | --- | --- | --- |
| Spiny chromis (Acanthochromis polyacanthus) | si:dkey-256h2.1 (ENSAPOG00000020584) | 59.79% | 60.42% | 75 | 78.75 | Yes |
| Midas cichlid (Amphilophus citrinellus) | si:dkey-256h2.1 (ENSACIG00000009522) | 57.40% | 58.61% | 75 | 80.24 | Yes |
| Clown anemonefish (Amphiprion ocellaris) | si:dkey-256h2.1 (ENSAOCG00000014235) | 59.49% | 60.12% | 75 | 78.74 | Yes |
| Orange clownfish (Amphiprion percula) | si:dkey-256h2.1 (ENSAPEG00000023979) | 59.19% | 59.82% | 75 | 78.73 | Yes |
| Climbing perch (Anabas testudineus) | si:dkey-256h2.1 (ENSATEG00000004158) | 59.88% | 60.42% | 75 | n/a | Yes |
| Eastern happy (Astatotilapia calliptera) | si:dkey-256h2.1 (ENSACLG00000007255) | 59.88% | 60.42% | 75 | 90.62 | Yes |
| Mexican tetra (Astyanax mexicanus) | si:dkey-256h2.1 (ENSAMXG00000024641) | 64.31% | 64.50% | 100 | 92.97 | Yes |
| Siamese fighting fish (Betta splendens) | si:dkey-256h2.1 (ENSBSLG00000018621) | 60.15% | 60.88% | 75 | 78.28 | Yes |
| Elephant shark (Callorhinchus milii) | si:dkey-256h2.1 (ENSCMIG00000008244) | 47.17% | 50.30% | n/a | 63.57 | Yes |
| Goldfish (Carassius auratus) | si:dkey-256h2.1 (ENSCARG00000023971) | 72.40% | 73.72% | 100 | 99.2 | Yes |
| Atlantic herring (Clupea harengus) | si:dkey-256h2.1 (ENSCHAG00000007434) | 63.85% | 60.57% | 0 | 95.19 | Yes |
| Channel bull blenny (Cottoperca gobio) | si:dkey-256h2.1 (ENSCGOG00000012000) | 59.19% | 59.82% | 75 | 78.81 | Yes |
| Lumpfish (Cyclopterus lumpus) | si:dkey-256h2.1 (ENSCLMG00005002078) | 57.27% | 57.10% | 75 | 78.16 | Yes |
| Tongue sole (Cynoglossus semilaevis) | si:dkey-256h2.1 (ENSCSEG00000004117) | 56.39% | 58.01% | 75 | 78.09 | Yes |
| Sheepshead minnow (Cyprinodon variegatus) | si:dkey-256h2.1 (ENSCVAG00000014085) | 59.79% | 60.42% | 75 | 89.63 | Yes |
| Common carp (Cyprinus carpio carpio) | si:dkey-256h2.1 (ENSCCRG00000042019) | 72.38% | 74.02% | 100 | 0 | Yes |
| Denticle herring (Denticeps clupeoides) | si:dkey-256h2.1 (ENSDCDG00000016996) | 55.97% | 61.63% | 25 | 86.73 | Yes |
| European seabass (Dicentrarchus labrax) | si:dkey-256h2.1 (ENSDLAG00005003903) | 59.82% | 60.73% | 75 | 0 | Yes |
| Electric eel (Electrophorus electricus) | si:dkey-256h2.1 (ENSEEEG00000004685) | 61.33% | 55.59% | 50 | 83.55 | Yes |
| Hagfish (Eptatretus burgeri) | ENSEBUG00000009002 | 46.60% | 45.62% | n/a | 36.11 | No |
| Reedfish (Erpetoichthys calabaricus) | si:dkey-256h2.1 (ENSECRG00000012683) | 50.85% | 54.08% | 0 | 75.85 | Yes |
| Northern pike (Esox lucius) | si:dkey-256h2.1 (ENSELUG00000013823) | 60.18% | 61.63% | 75 | 89.75 | Yes |
| Mummichog (Fundulus heteroclitus) | si:dkey-256h2.1 (ENSFHEG00000009967) | 60.51% | 60.88% | 50 | 86.14 | Yes |
| Atlantic cod (Gadus morhua) | si:dkey-256h2.1 (ENSGMOG00000006579) | 56.41% | 56.50% | 75 | n/a | Yes |
| Stickleback (Gasterosteus aculeatus) | si:dkey-256h2.1 (ENSGACG00000012932) | 62.95% | 58.01% | 75 | 80.11 | Yes |
| Burton's mouthbrooder (Haplochromis burtoni) | si:dkey-256h2.1 (ENSHBUG00000012743) | 60.39% | 60.57% | 75 | 91.01 | Yes |
| Tiger tail seahorse (Hippocampus comes) | si:dkey-256h2.1 (ENSHCOG00000006014) | 58.12% | 58.91% | 75 | 85.48 | Yes |
| Huchen (Hucho hucho) | si:dkey-256h2.1 (ENSHHUG00000028412) | 59.74% | 61.63% | 75 | 86.88 | Yes |
| Channel catfish (Ictalurus punctatus) | si:dkey-256h2.1 (ENSIPUG00000005674) | 62.87% | 63.44% | 100 | 92.15 | Yes |
| Mangrove rivulus (Kryptolebias marmoratus) | si:dkey-256h2.1 (ENSKMAG00000019951) | 59.34% | 59.97% | 75 | 82.18 | Yes |
| Ballan wrasse (Labrus bergylta) | si:dkey-256h2.1 (ENSLBEG00000007044) | 59.40% | 60.12% | 75 | 84.75 | Yes |
| Large yellow croaker (Larimichthys crocea) | si:dkey-256h2.1 (ENSLCRG00005005036) | 55.54% | 56.04% | 25 | 79.6 | Yes |
| Large yellow croaker (Larimichthys crocea) | si:dkey-256h2.1 (ENSLCRG00005006152) | 59.02% | 59.82% | 25 | 72.03 | Yes |
| Barramundi perch (Lates calcarifer) | si:dkey-256h2.1 (ENSLCAG00010027023) | 59.82% | 61.18% | 75 | 84.27 | Yes |
| Coelacanth (Latimeria chalumnae) | ENSLACG00000001807 | 56.18% | 37.76% | 0 | 47.05 | No |
| Spotted gar (Lepisosteus oculatus) | si:dkey-256h2.1 (ENSLOCG00000011030) | 54.35% | 56.65% | 0 | 80.59 | Yes |
| Zig-zag eel (Mastacembelus armatus) | si:dkey-256h2.1 (ENSMAMG00000011394) | 61.25% | 62.08% | 75 | n/a | Yes |
| Zebra mbuna (Maylandia zebra) | si:dkey-256h2.1 (ENSMZEG00005015638) | 60.39% | 60.57% | 75 | 90.99 | Yes |
| Pinecone soldierfish (Myripristis murdjan) | si:dkey-256h2.1 (ENSMMDG00005007230) | 59.58% | 60.57% | 75 | 85.3 | Yes |
| Lyretail cichlid (Neolamprologus brichardi) | si:dkey-256h2.1 (ENSNBRG00000015584) | 49.03% | 38.22% | 75 | 65.89 | Yes |
| Turquoise killifish (Nothobranchius furzeri) | si:dkey-256h2.1 (ENSNFUG00015010963) | 58.99% | 58.46% | 75 | 77.06 | Yes |
| Coho salmon (Oncorhynchus kisutch) | si:dkey-256h2.1 (ENSOKIG00005031356) | 59.15% | 61.03% | 75 | 85.91 | Yes |
| Rainbow trout (Oncorhynchus mykiss) | si:dkey-256h2.1 (ENSOMYG00000005444) | 49.45% | 40.94% | 75 | 0 | Yes |
| Rainbow trout (Oncorhynchus mykiss) | si:dkey-256h2.1 (ENSOMYG00000010802) | 59.15% | 61.03% | 50 | 0 | Yes |
| Chinook salmon (Oncorhynchus tshawytscha) | si:dkey-256h2.1 (ENSOTSG00005015396) | 59.30% | 61.18% | 75 | 86.32 | Yes |
| Chinook salmon (Oncorhynchus tshawytscha) | si:dkey-256h2.1 (ENSOTSG00005021181) | 46.02% | 36.71% | 75 | 63.16 | Yes |
| Nile tilapia (Oreochromis niloticus) | si:dkey-256h2.1 (ENSONIG00000006503) | 60.54% | 60.73% | 75 | 84.99 | Yes |
| Javanese ricefish (Oryzias javanicus) | si:dkey-256h2.1 (ENSOJAG00000003193) | 58.68% | 59.21% | 25 | 76.68 | Yes |
| Japanese medaka HdrR (Oryzias latipes) | si:dkey-256h2.1 (ENSORLG00000016352) | 55.36% | 56.19% | 25 | 79.03 | Yes |
| Indian medaka (Oryzias melastigma) | si:dkey-256h2.1 (ENSOMEG00000022039) | 58.58% | 58.76% | 25 | 81.81 | Yes |
| Chinese medaka (Oryzias sinensis) | si:dkey-256h2.1 (ENSOSIG00000012649) | 57.71% | 37.31% | 0 | 70.03 | Yes |
| Paramormyrops kingsleyae (Paramormyrops kingsleyae) | si:dkey-256h2.1 (ENSPKIG00000010958) | 55.08% | 56.50% | 75 | 83.04 | Yes |
| Lamprey (Petromyzon marinus) | ENSPMAG00000008840 | 58.06% | 24.47% | n/a | 46.9 | No |
| Amazon molly (Poecilia formosa) | si:dkey-256h2.1 (ENSPFOG00000005341) | 60.88% | 60.88% | 75 | 84.91 | Yes |
| Sailfin molly (Poecilia latipinna) | si:dkey-256h2.1 (ENSPLAG00000011765) | 60.73% | 60.73% | 25 | 84.92 | Yes |
| Guppy (Poecilia reticulata) | si:dkey-256h2.1 (ENSPREG00000001398) | 60.21% | 59.67% | 75 | 84.6 | Yes |
| Makobe Island cichlid (Pundamilia nyererei) | si:dkey-256h2.1 (ENSPNYG00000021594) | 57.67% | 54.53% | 75 | 78.79 | Yes |
| Red-bellied piranha (Pygocentrus nattereri) | si:dkey-256h2.1 (ENSPNAG00000016358) | 66.10% | 65.11% | 75 | 92.83 | Yes |
| Atlantic salmon (Salmo salar) | si:dkey-256h2.1 (ENSSSAG00000042841) | 54.75% | 54.83% | 75 | 0 | Yes |
| Brown trout (Salmo trutta) | si:dkey-256h2.1 (ENSSTUG00000031716) | 55.02% | 38.07% | 75 | 73.06 | Yes |
| Brown trout (Salmo trutta) | si:dkey-256h2.1 (ENSSTUG00000044186) | 59.88% | 61.78% | 75 | 83.91 | Yes |
| Pike-perch (Sander lucioperca) | si:dkey-256h2.1 (ENSSLUG00000008787) | 58.78% | 59.67% | 75 | 90.95 | Yes |
| Pike-perch (Sander lucioperca) | si:dkey-256h2.1 (ENSSLUG00000008798) | 64.69% | 36.25% | 75 | 73.87 | Yes |
| Asian bonytongue (Scleropages formosus) | si:dkey-256h2.1 (ENSSFOG00015020856) | 57.04% | 58.76% | 75 | 77.86 | Yes |
| Turbot (Scophthalmus maximus) | si:dkey-256h2.1 (ENSSMAG00000014674) | 59.41% | 60.57% | 75 | 0 | Yes |
| Greater amberjack (Seriola dumerili) | si:dkey-256h2.1 (ENSSDUG00000011889) | 58.54% | 60.57% | 75 | 76.01 | Yes |
| Yellowtail amberjack (Seriola lalandi dorsalis) | si:dkey-256h2.1 (ENSSLDG00000015539) | 57.37% | 59.37% | 75 | 76.97 | Yes |
| Gilthead seabream (Sparus aurata) | si:dkey-256h2.1 (ENSSAUG00010017367) | 58.05% | 57.70% | 75 | 73.56 | Yes |
| Bicolor damselfish (Stegastes partitus) | si:dkey-256h2.1 (ENSSPAG00000014407) | 58.49% | 60.88% | 75 | 78.68 | Yes |
| Tetraodon (Tetraodon nigroviridis) | si:dkey-256h2.1 (ENSTNIG00000000520) | 61.58% | 56.65% | 75 | 83.57 | Yes |
| Monterrey platyfish (Xiphophorus couchianus) | si:dkey-256h2.1 (ENSXCOG00000007287) | 60.55% | 60.27% | 75 | 0 | Yes |
| Platyfish (Xiphophorus maculatus) | si:dkey-256h2.1 (ENSXMAG00000018794) | 60.27% | 60.27% | 75 | 82.43 | Yes |

## Slide 2
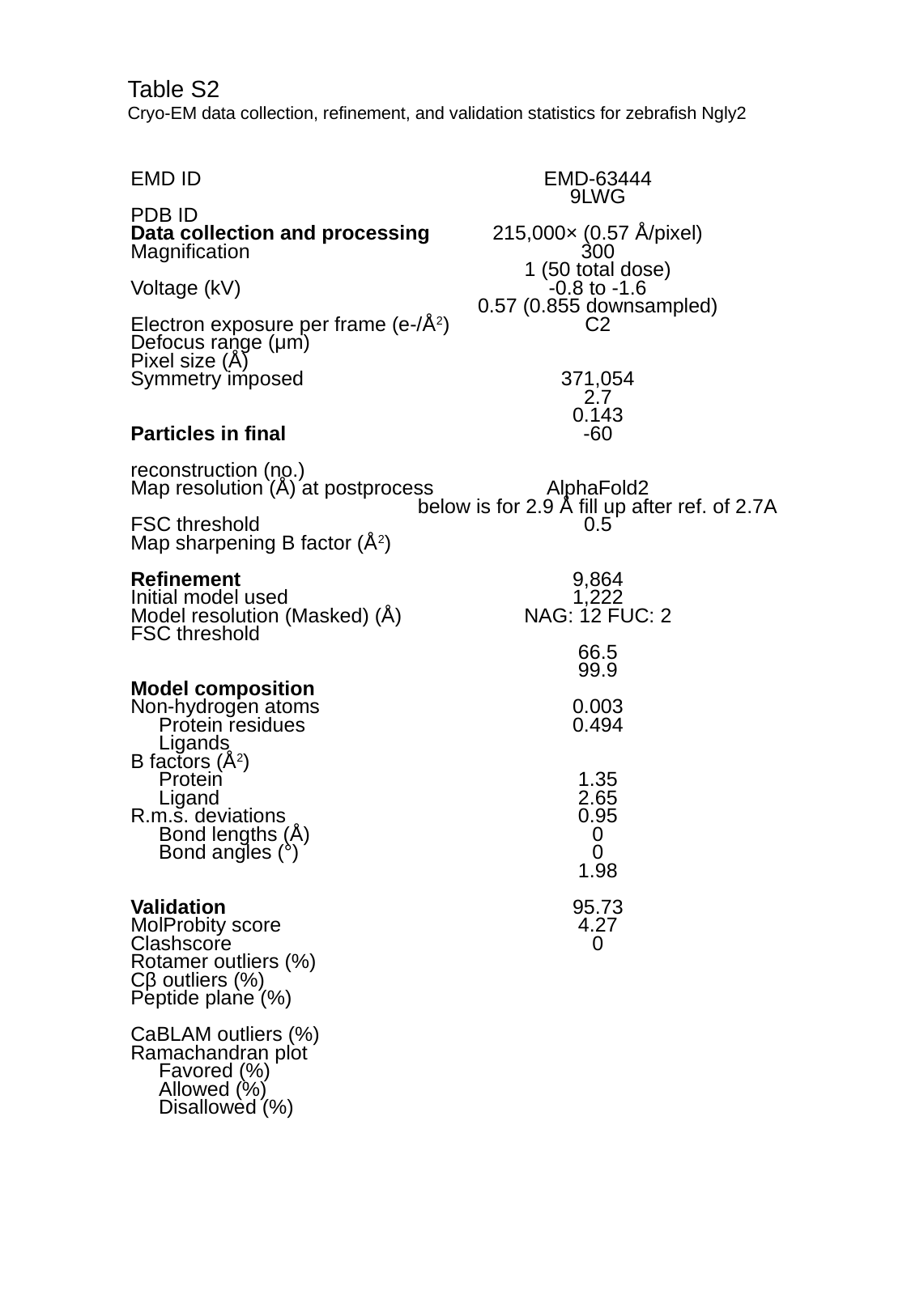

Table S2
Cryo-EM data collection, refinement, and validation statistics for zebrafish Ngly2
EMD ID
PDB ID
Data collection and processing
Magnification
Voltage (kV)
Electron exposure per frame (e-/Å2)
Defocus range (μm)
Pixel size (Å)
Symmetry imposed
Particles in final
reconstruction (no.)
Map resolution (Å) at postprocess
FSC threshold
Map sharpening B factor (Å2)
Refinement
Initial model used
Model resolution (Masked) (Å)
FSC threshold
Model composition
Non-hydrogen atoms
 Protein residues
 Ligands
B factors (Å2)
 Protein
 Ligand
R.m.s. deviations
 Bond lengths (Å)
 Bond angles (°)
Validation
MolProbity score
Clashscore
Rotamer outliers (%)
Cβ outliers (%)
Peptide plane (%)
CaBLAM outliers (%)
Ramachandran plot
 Favored (%)
 Allowed (%)
 Disallowed (%)
EMD-63444
9LWG
215,000× (0.57 Å/pixel)
300
1 (50 total dose)
-0.8 to -1.6
0.57 (0.855 downsampled)
C2
371,054
2.7
0.143
-60
AlphaFold2
below is for 2.9 Å fill up after ref. of 2.7A
0.5
9,864
1,222
NAG: 12 FUC: 2
66.5
99.9
0.003
0.494
1.35
2.65
0.95
0
0
1.98
95.73
4.27
0

## Slide 3
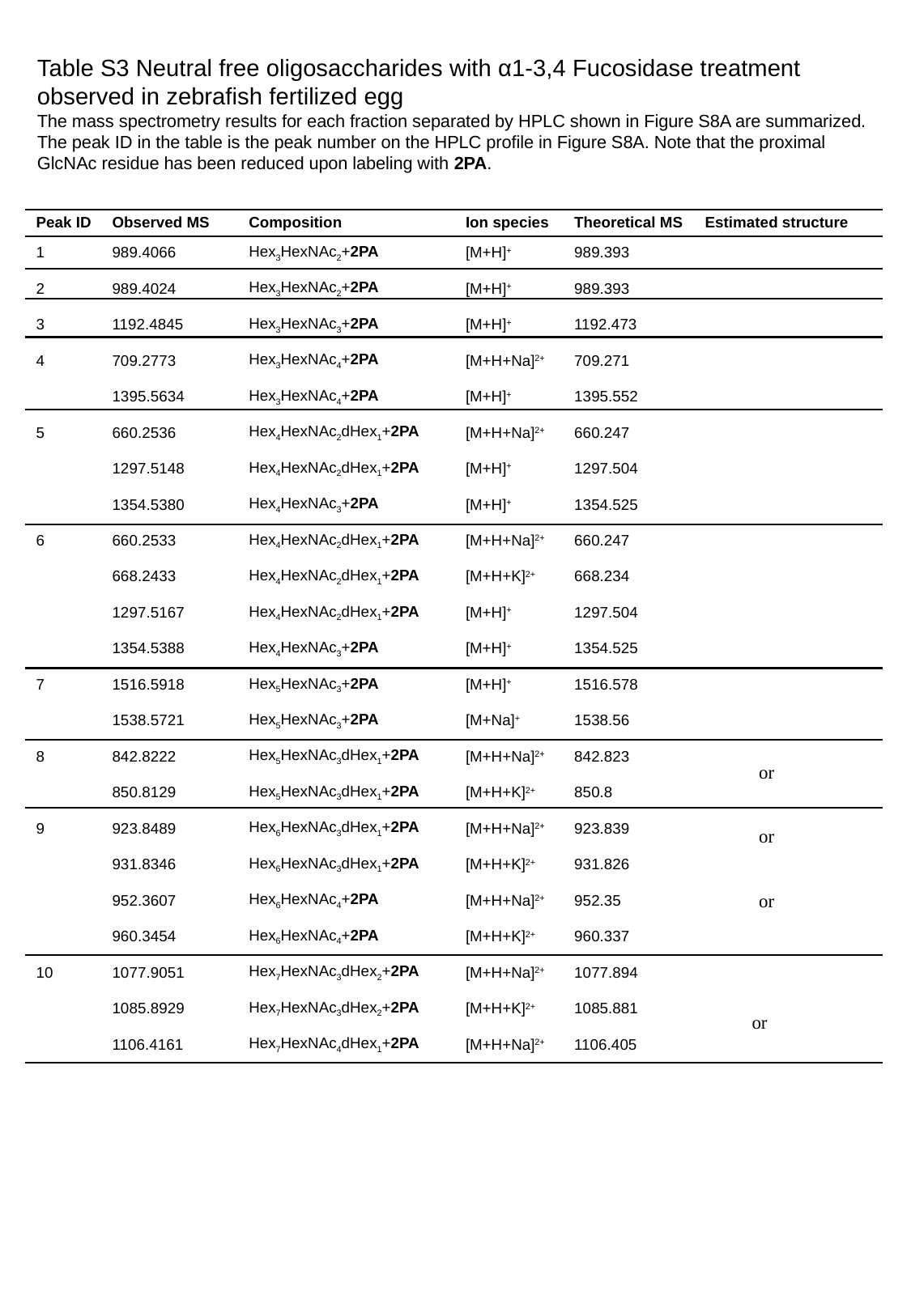

Table S3 Neutral free oligosaccharides with α1-3,4 Fucosidase treatment observed in zebrafish fertilized egg
The mass spectrometry results for each fraction separated by HPLC shown in Figure S8A are summarized. The peak ID in the table is the peak number on the HPLC profile in Figure S8A. Note that the proximal GlcNAc residue has been reduced upon labeling with 2PA.
| Peak ID | Observed MS | Composition | Ion species | Theoretical MS | Estimated structure |
| --- | --- | --- | --- | --- | --- |
| 1 | 989.4066 | Hex3HexNAc2+2PA | [M+H]+ | 989.393 | |
| 2 | 989.4024 | Hex3HexNAc2+2PA | [M+H]+ | 989.393 | |
| 3 | 1192.4845 | Hex3HexNAc3+2PA | [M+H]+ | 1192.473 | |
| 4 | 709.2773 | Hex3HexNAc4+2PA | [M+H+Na]2+ | 709.271 | |
| | 1395.5634 | Hex3HexNAc4+2PA | [M+H]+ | 1395.552 | |
| 5 | 660.2536 | Hex4HexNAc2dHex1+2PA | [M+H+Na]2+ | 660.247 | |
| | 1297.5148 | Hex4HexNAc2dHex1+2PA | [M+H]+ | 1297.504 | |
| | 1354.5380 | Hex4HexNAc3+2PA | [M+H]+ | 1354.525 | |
| 6 | 660.2533 | Hex4HexNAc2dHex1+2PA | [M+H+Na]2+ | 660.247 | |
| | 668.2433 | Hex4HexNAc2dHex1+2PA | [M+H+K]2+ | 668.234 | |
| | 1297.5167 | Hex4HexNAc2dHex1+2PA | [M+H]+ | 1297.504 | |
| | 1354.5388 | Hex4HexNAc3+2PA | [M+H]+ | 1354.525 | |
| 7 | 1516.5918 | Hex5HexNAc3+2PA | [M+H]+ | 1516.578 | |
| | 1538.5721 | Hex5HexNAc3+2PA | [M+Na]+ | 1538.56 | |
| 8 | 842.8222 | Hex5HexNAc3dHex1+2PA | [M+H+Na]2+ | 842.823 | |
| | 850.8129 | Hex5HexNAc3dHex1+2PA | [M+H+K]2+ | 850.8 | |
| 9 | 923.8489 | Hex6HexNAc3dHex1+2PA | [M+H+Na]2+ | 923.839 | |
| | 931.8346 | Hex6HexNAc3dHex1+2PA | [M+H+K]2+ | 931.826 | |
| | 952.3607 | Hex6HexNAc4+2PA | [M+H+Na]2+ | 952.35 | |
| | 960.3454 | Hex6HexNAc4+2PA | [M+H+K]2+ | 960.337 | |
| 10 | 1077.9051 | Hex7HexNAc3dHex2+2PA | [M+H+Na]2+ | 1077.894 | |
| | 1085.8929 | Hex7HexNAc3dHex2+2PA | [M+H+K]2+ | 1085.881 | |
| | 1106.4161 | Hex7HexNAc4dHex1+2PA | [M+H+Na]2+ | 1106.405 | |
or
or
or
or

## Slide 4
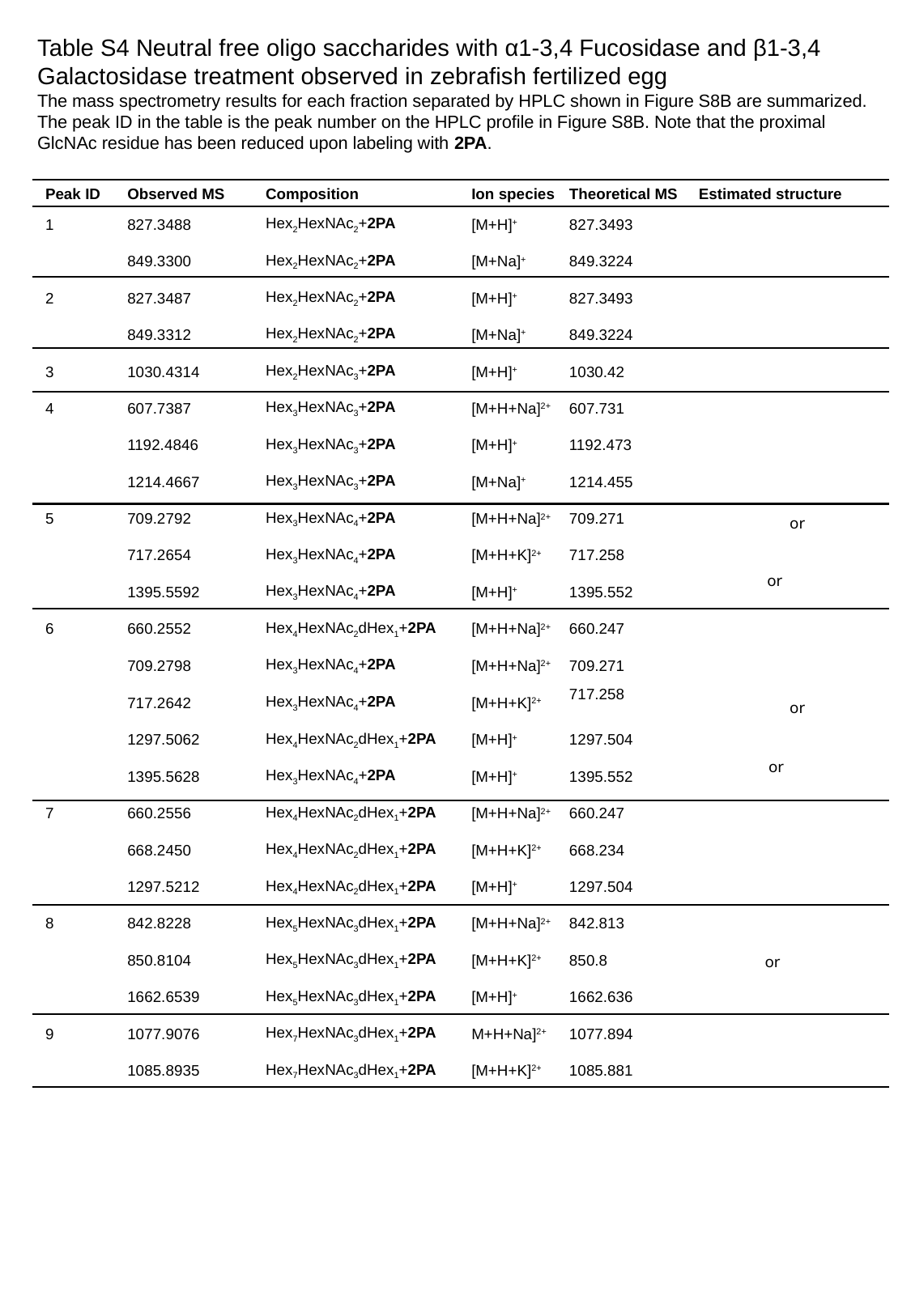

Table S4 Neutral free oligo saccharides with α1-3,4 Fucosidase and β1-3,4 Galactosidase treatment observed in zebrafish fertilized egg
The mass spectrometry results for each fraction separated by HPLC shown in Figure S8B are summarized. The peak ID in the table is the peak number on the HPLC profile in Figure S8B. Note that the proximal GlcNAc residue has been reduced upon labeling with 2PA.
| Peak ID | Observed MS | Composition | Ion species | Theoretical MS | Estimated structure |
| --- | --- | --- | --- | --- | --- |
| 1 | 827.3488 | Hex2HexNAc2+2PA | [M+H]+ | 827.3493 | |
| | 849.3300 | Hex2HexNAc2+2PA | [M+Na]+ | 849.3224 | |
| 2 | 827.3487 | Hex2HexNAc2+2PA | [M+H]+ | 827.3493 | |
| | 849.3312 | Hex2HexNAc2+2PA | [M+Na]+ | 849.3224 | |
| 3 | 1030.4314 | Hex2HexNAc3+2PA | [M+H]+ | 1030.42 | |
| 4 | 607.7387 | Hex3HexNAc3+2PA | [M+H+Na]2+ | 607.731 | |
| | 1192.4846 | Hex3HexNAc3+2PA | [M+H]+ | 1192.473 | |
| | 1214.4667 | Hex3HexNAc3+2PA | [M+Na]+ | 1214.455 | |
| 5 | 709.2792 | Hex3HexNAc4+2PA | [M+H+Na]2+ | 709.271 | |
| | 717.2654 | Hex3HexNAc4+2PA | [M+H+K]2+ | 717.258 | |
| | 1395.5592 | Hex3HexNAc4+2PA | [M+H]+ | 1395.552 | |
| 6 | 660.2552 | Hex4HexNAc2dHex1+2PA | [M+H+Na]2+ | 660.247 | |
| | 709.2798 | Hex3HexNAc4+2PA | [M+H+Na]2+ | 709.271 | |
| | 717.2642 | Hex3HexNAc4+2PA | [M+H+K]2+ | 717.258 | |
| | 1297.5062 | Hex4HexNAc2dHex1+2PA | [M+H]+ | 1297.504 | |
| | 1395.5628 | Hex3HexNAc4+2PA | [M+H]+ | 1395.552 | |
| 7 | 660.2556 | Hex4HexNAc2dHex1+2PA | [M+H+Na]2+ | 660.247 | |
| | 668.2450 | Hex4HexNAc2dHex1+2PA | [M+H+K]2+ | 668.234 | |
| | 1297.5212 | Hex4HexNAc2dHex1+2PA | [M+H]+ | 1297.504 | |
| 8 | 842.8228 | Hex5HexNAc3dHex1+2PA | [M+H+Na]2+ | 842.813 | |
| | 850.8104 | Hex5HexNAc3dHex1+2PA | [M+H+K]2+ | 850.8 | |
| | 1662.6539 | Hex5HexNAc3dHex1+2PA | [M+H]+ | 1662.636 | |
| 9 | 1077.9076 | Hex7HexNAc3dHex1+2PA | M+H+Na]2+ | 1077.894 | |
| | 1085.8935 | Hex7HexNAc3dHex1+2PA | [M+H+K]2+ | 1085.881 | |
or
or
or
or
or

## Slide 5
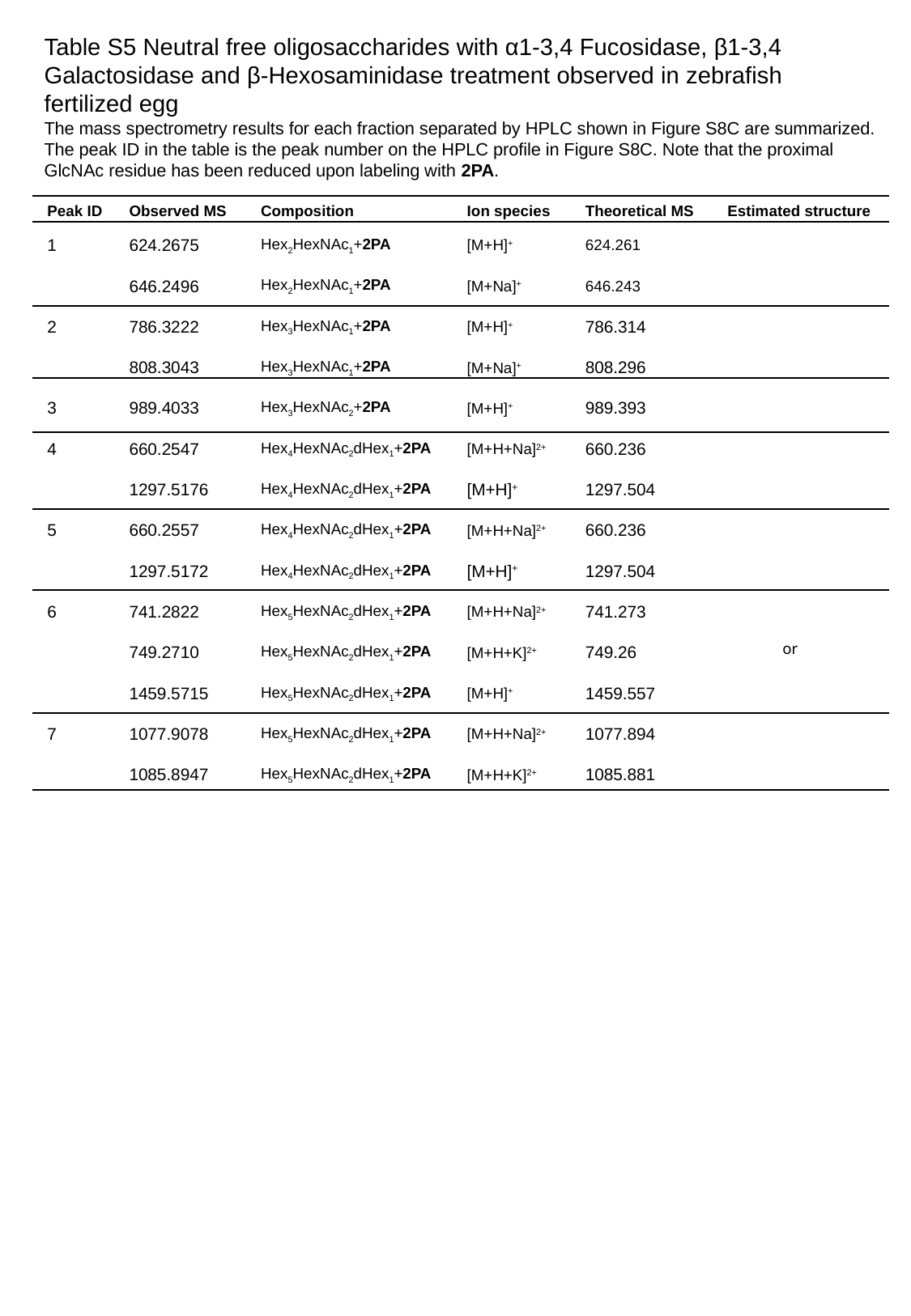

Table S5 Neutral free oligosaccharides with α1-3,4 Fucosidase, β1-3,4 Galactosidase and β-Hexosaminidase treatment observed in zebrafish fertilized egg
The mass spectrometry results for each fraction separated by HPLC shown in Figure S8C are summarized. The peak ID in the table is the peak number on the HPLC profile in Figure S8C. Note that the proximal GlcNAc residue has been reduced upon labeling with 2PA.
| Peak ID | Observed MS | Composition | Ion species | Theoretical MS | Estimated structure |
| --- | --- | --- | --- | --- | --- |
| 1 | 624.2675 | Hex2HexNAc1+2PA | [M+H]+ | 624.261 | |
| | 646.2496 | Hex2HexNAc1+2PA | [M+Na]+ | 646.243 | |
| 2 | 786.3222 | Hex3HexNAc1+2PA | [M+H]+ | 786.314 | |
| | 808.3043 | Hex3HexNAc1+2PA | [M+Na]+ | 808.296 | |
| 3 | 989.4033 | Hex3HexNAc2+2PA | [M+H]+ | 989.393 | |
| 4 | 660.2547 | Hex4HexNAc2dHex1+2PA | [M+H+Na]2+ | 660.236 | |
| | 1297.5176 | Hex4HexNAc2dHex1+2PA | [M+H]+ | 1297.504 | |
| 5 | 660.2557 | Hex4HexNAc2dHex1+2PA | [M+H+Na]2+ | 660.236 | |
| | 1297.5172 | Hex4HexNAc2dHex1+2PA | [M+H]+ | 1297.504 | |
| 6 | 741.2822 | Hex5HexNAc2dHex1+2PA | [M+H+Na]2+ | 741.273 | |
| | 749.2710 | Hex5HexNAc2dHex1+2PA | [M+H+K]2+ | 749.26 | |
| | 1459.5715 | Hex5HexNAc2dHex1+2PA | [M+H]+ | 1459.557 | |
| 7 | 1077.9078 | Hex5HexNAc2dHex1+2PA | [M+H+Na]2+ | 1077.894 | |
| | 1085.8947 | Hex5HexNAc2dHex1+2PA | [M+H+K]2+ | 1085.881 | |
or
