# Supplementary material for: Structural characterization of zebrafish Ngly2, an ovary-enriched acid PNGase required for egg-free glycan production
Source: J Biol Chem. 2025 Nov 5;301(12):110906. doi: 10.1016/j.jbc.2025.110906 (PMC12753239; doi:10.1016/j.jbc.2025.110906)
Supplement: Supplemental Figures [file mmc2.pptx]

## Slide 1
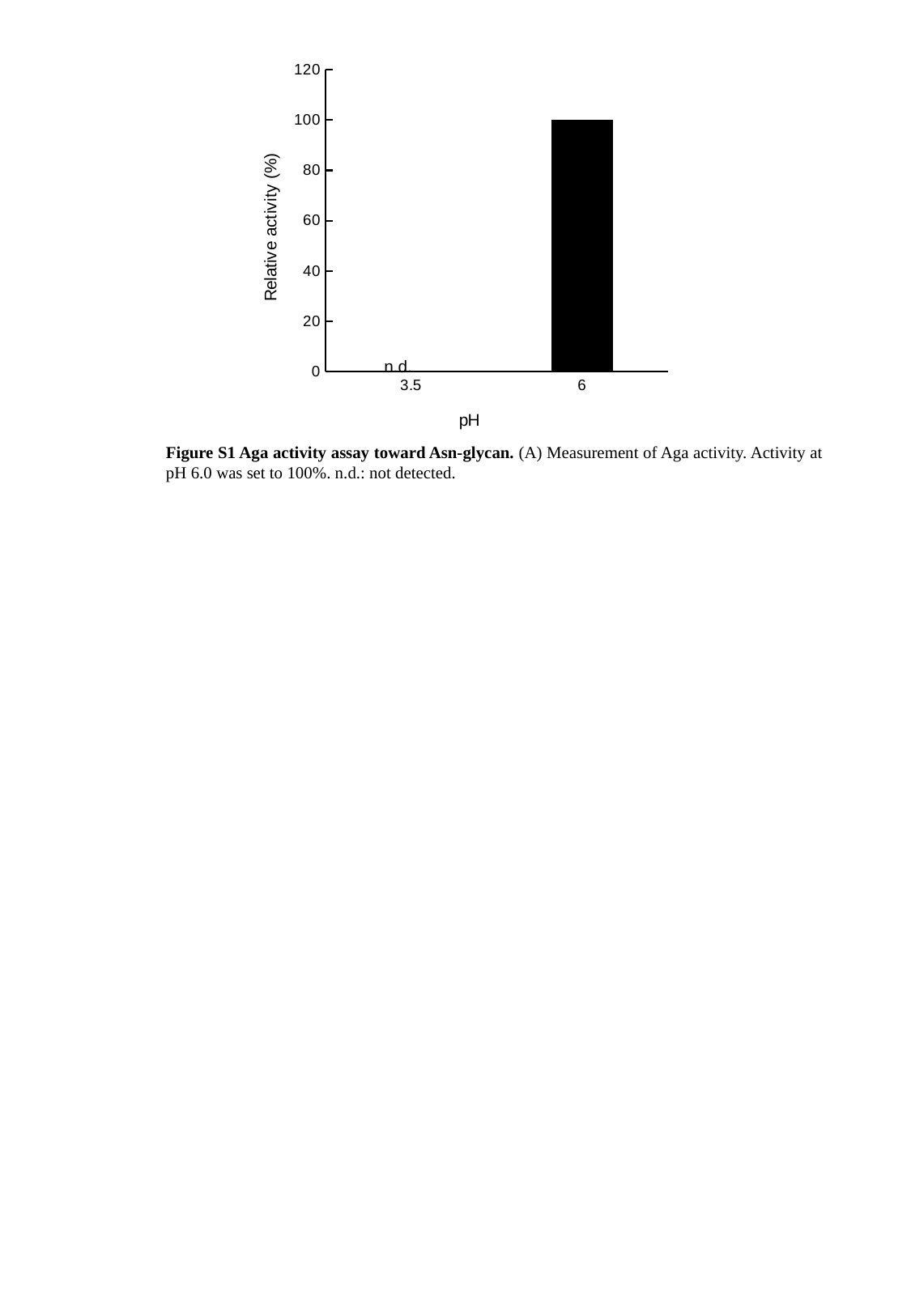

### Chart
| Category | |
|---|---|
| 3.5 | 0.0 |
| 6 | 100.0 |n.d.
Figure S1 Aga activity assay toward Asn-glycan. (A) Measurement of Aga activity. Activity at pH 6.0 was set to 100%. n.d.: not detected.

## Slide 2
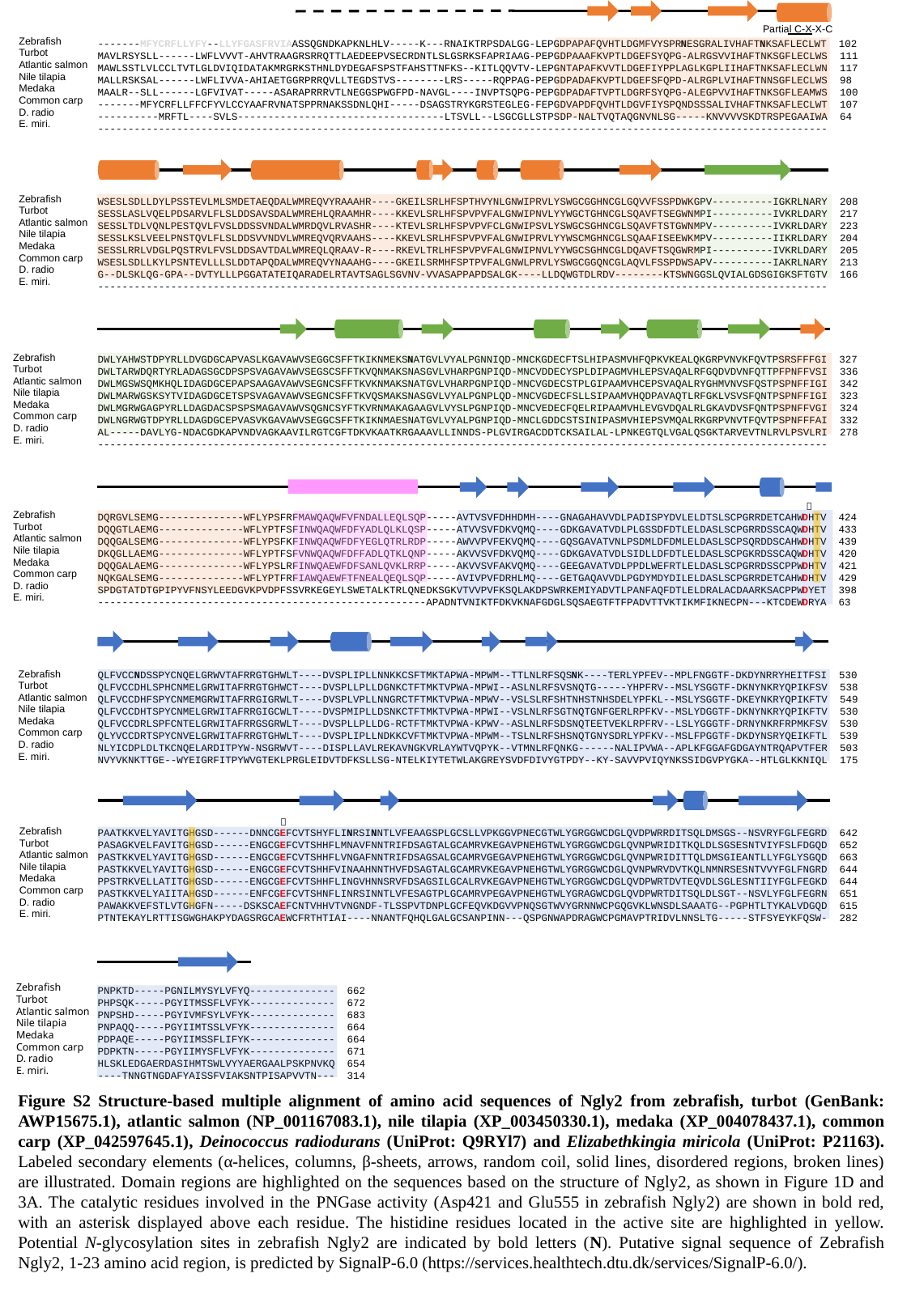

Partial C-X-X-C
 -------MFYCRFLLYFY--LLYFGASFRVIAASSQGNDKAPKNLHLV-----K---RNAIKTRPSDALGG-LEPGDPAPAFQVHTLDGMFVYSPRNESGRALIVHAFTNKSAFLECLWT 102
 MAVLRSYSLL------LWFLVVVT-AHVTRAAGRSRRQTTLAEDEEPVSECRDNTLSLGSRKSFAPRIAAG-PEPGDPAAAFKVPTLDGEFSYQPG-ALRGSVVIHAFTNKSGFLECLWS 111
 MAWLSSTLVLCCLTVTLGLDVIQIDATAKMRGRKSTHNLDYDEGAFSPSTFAHSTTNFKS--KITLQQVTV-LEPGNTAPAFKVVTLDGEFIYPPLAGLKGPLIIHAFTNKSAFLECLWN 117
 MALLRSKSAL------LWFLIVVA-AHIAETGGRPRRQVLLTEGDSTVS--------LRS-----RQPPAG-PEPGDPADAFKVPTLDGEFSFQPD-ALRGPLVIHAFTNNSGFLECLWS 98
 MAALR--SLL------LGFVIVAT-----ASARAPRRRVTLNEGGSPWGFPD-NAVGL----INVPTSQPG-PEPGDPADAFTVPTLDGRFSYQPG-ALEGPVVIHAFTNKSGFLEAMWS 100
 -------MFYCRFLLFFCFYVLCCYAAFRVNATSPPRNAKSSDNLQHI-----DSAGSTRYKGRSTEGLEG-FEPGDVAPDFQVHTLDGVFIYSPQNDSSSALIVHAFTNKSAFLECLWT 107
 ----------MRFTL----SVLS----------------------------------LTSVLL--LSGCGLLSTPSDP-NALTVQTAQGNVNLSG-----KNVVVVSKDTRSPEGAAIWA 64
 ------------------------------------------------------------------------------------------------------------------------
 WSESLSDLLDYLPSSTEVLMLSMDETAEQDALWMREQVYRAAAHR----GKEILSRLHFSPTHVYNLGNWIPRVLYSWGCGGHNCGLGQVVFSSPDWKGPV----------IGKRLNARY 208
 SESSLASLVQELPDSARVLFLSLDDSAVSDALWMREHLQRAAMHR----KKEVLSRLHFSPVPVFALGNWIPNVLYYWGCTGHNCGLSQAVFTSEGWNMPI----------IVKRLDARY 217
 SESSLTDLVQNLPESTQVLFVSLDDSSVNDALWMRDQVLRVASHR----KTEVLSRLHFSPVPVFCLGNWIPSVLYSWGCSGHNCGLSQAVFTSTGWNMPV----------IVKRLDARY 223
 SESSLKSLVEELPNSTQVLFLSLDDSVVNDVLWMREQVQRVAAHS----KKEVLSRLHFSPVPVFALGNWIPRVLYYWSCMGHNCGLSQAAFISEEWKMPV----------IIKRLDARY 204
 SESSLRRLVDGLPQSTRVLFVSLDDSAVTDALWMREQLQRAAV-R----RKEVLTRLHFSPVPVFALGNWIPNVLYYWGCSGHNCGLDQAVFTSQGWRMPI----------IVKRLDARY 205
 WSESLSDLLKYLPSNTEVLLLSLDDTAPQDALWMREQVYNAAAHG----GKEILSRMHFSPTPVFALGNWLPRVLYSWGCGGQNCGLAQVLFSSPDWSAPV----------IAKRLNARY 213
 G--DLSKLQG-GPA--DVTYLLLPGGATATEIQARADELRTAVTSAGLSGVNV-VVASAPPAPDSALGK----LLDQWGTDLRDV--------KTSWNGGSLQVIALGDSGIGKSFTGTV 166
 ------------------------------------------------------------------------------------------------------------------------
 DWLYAHWSTDPYRLLDVGDGCAPVASLKGAVAWVSEGGCSFFTKIKNMEKSNATGVLVYALPGNNIQD-MNCKGDECFTSLHIPASMVHFQPKVKEALQKGRPVNVKFQVTPSRSFFFGI 327
 DWLTARWDQRTYRLADAGSGCDPSPSVAGAVAWVSEGSCSFFTKVQNMAKSNASGVLVHARPGNPIQD-MNCVDDECYSPLDIPAGMVHLEPSVAQALRFGQDVDVNFQTTPFPNFFVSI 336
 DWLMGSWSQMKHQLIDAGDGCEPAPSAAGAVAWVSEGNCSFFTKVKNMAKSNATGVLVHARPGNPIQD-MNCVGDECSTPLGIPAAMVHCEPSVAQALRYGHMVNVSFQSTPSPNFFIGI 342
 DWLMARWGSKSYTVIDAGDGCETSPSVAGAVAWVSEGNCSFFTKVQSMAKSNASGVLVYALPGNPLQD-MNCVGDECFSLLSIPAAMVHQDPAVAQTLRFGKLVSVSFQNTPSPNFFIGI 323
 DWLMGRWGAGPYRLLDAGDACSPSPSMAGAVAWVSQGNCSYFTKVRNMAKAGAAGVLVYSLPGNPIQD-MNCVEDECFQELRIPAAMVHLEVGVDQALRLGKAVDVSFQNTPSPNFFVGI 324
 DWLNGRWGTDPYRLLDAGDGCEPVASVKGAVAWVSEGGCSFFTKIKNMAESNATGVLVYALPGNPIQD-MNCLGDDCSTSINIPASMVHIEPSVMQALRKGRPVNVTFQVTPSPNFFFAI 332
 AL-----DAVLYG-NDACGDKAPVNDVAGKAAVILRGTCGFTDKVKAATKRGAAAVLLINNDS-PLGVIRGACDDTCKSAILAL-LPNKEGTQLVGALQSGKTARVEVTNLRVLPSVLRI 278
 ------------------------------------------------------------------------------------------------------------------------
 DQRGVLSEMG--------------WFLYPSFRFMAWQAQWFVFNDALLEQLSQP-----AVTVSVFDHHDMH----GNAGAHAVVDLPADISPYDVLELDTSLSCPGRRDETCAHWDHTV 424
 DQQGTLAEMG--------------WFLYPTFSFINWQAQWFDFYADLQLKLQSP-----ATVVSVFDKVQMQ----GDKGAVATVDLPLGSSDFDTLELDASLSCPGRRDSSCAQWDHTV 433
 DQQGALSEMG--------------WFLYPSFKFINWQAQWFDFYEGLQTRLRDP-----AWVVPVFEKVQMQ----GQSGAVATVNLPSDMLDFDMLELDASLSCPSQRDDSCAHWDHTV 439
 DKQGLLAEMG--------------WFLYPTFSFVNWQAQWFDFFADLQTKLQNP-----AKVVSVFDKVQMQ----GDKGAVATVDLSIDLLDFDTLELDASLSCPGKRDSSCAQWDHTV 420
 DQQGALAEMG--------------WFLYPSLRFINWQAEWFDFSANLQVKLRRP-----AKVVSVFAKVQMQ----GEEGAVATVDLPPDLWEFRTLELDASLSCPGRRDSSCPPWDHTV 421
 NQKGALSEMG--------------WFLYPTFRFIAWQAEWFTFNEALQEQLSQP-----AVIVPVFDRHLMQ----GETGAQAVVDLPGDYMDYDILELDASLSCPGRRDETCAHWDHTV 429
 SPDGTATDTGPIPYVFNSYLEEDGVKPVDPFSSVRKEGEYLSWETALKTRLQNEDKSGKVTVVPVFKSQLAKDPSWRKEMIYADVTLPANFAQFDTLELDRALACDAARKSACPPWDYET 398
 ------------------------------------------------------APADNTVNIKTFDKVKNAFGDGLSQSAEGTFTFPADVTTVKTIKMFIKNECPN---KTCDEWDRYA 63
 QLFVCCNDSSPYCNQELGRWVTAFRRGTGHWLT----DVSPLIPLLNNKKCSFTMKTAPWA-MPWM--TTLNLRFSQSNK----TERLYPFEV--MPLFNGGTF-DKDYNRRYHEITFSI 530
 QLFVCCDHLSPHCNMELGRWITAFRRGTGHWCT----DVSPLLPLLDGNKCTFTMKTVPWA-MPWI--ASLNLRFSVSNQTG-----YHPFRV--MSLYSGGTF-DKNYNKRYQPIKFSV 538
 QLFVCCDHFSPYCNMEMGRWITAFRRGIGRWLT----DVSPLVPLLNNGRCTFTMKTVPWA-MPWV--VSLSLRFSHTNHSTNHSDELYPFKL--MSLYSGGTF-DKEYNKRYQPIKFTV 549
 QLFVCCDHTSPYCNMELGRWITAFRRGIGCWLT----DVSPMIPLLDSNKCTFTMKTVPWA-MPWI--VSLNLRFSGTNQTGNFGERLRPFKV--MSLYDGGTF-DKNYNKRYQPIKFTV 530
 QLFVCCDRLSPFCNTELGRWITAFRRGSGRWLT----DVSPLLPLLDG-RCTFTMKTVPWA-KPWV--ASLNLRFSDSNQTEETVEKLRPFRV--LSLYGGGTF-DRNYNKRFRPMKFSV 530
 QLYVCCDRTSPYCNVELGRWITAFRRGTGHWLT----DVSPLIPLLNDKKCVFTMKTVPWA-MPWM--TSLNLRFSHSNQTGNYSDRLYPFKV--MSLFPGGTF-DKDYNSRYQEIKFTL 539
 NLYICDPLDLTKCNQELARDITPYW-NSGRWVT----DISPLLAVLREKAVNGKVRLAYWTVQPYK--VTMNLRFQNKG------NALIPVWA--APLKFGGAFGDGAYNTRQAPVTFER 503
 NVYVKNKTTGE--WYEIGRFITPYWVGTEKLPRGLEIDVTDFKSLLSG-NTELKIYTETWLAKGREYSVDFDIVYGTPDY--KY-SAVVPVIQYNKSSIDGVPYGKA--HTLGLKKNIQL 175
 PAATKKVELYAVITGHGSD------DNNCGEFCVTSHYFLINRSINNTLVFEAAGSPLGCSLLVPKGGVPNECGTWLYGRGGWCDGLQVDPWRRDITSQLDMSGS--NSVRYFGLFEGRD 642
 PASAGKVELFAVITGHGSD------ENGCGEFCVTSHHFLMNAVFNNTRIFDSAGTALGCAMRVKEGAVPNEHGTWLYGRGGWCDGLQVNPWRIDITKQLDLSGSESNTVIYFSLFDGQD 652
 PASTKKVELYAVITGHGSD------ENGCGEFCVTSHHFLVNGAFNNTRIFDSAGSALGCAMRVGEGAVPNEHGTWLYGRGGWCDGLQVNPWRIDITTQLDMSGIEANTLLYFGLYSGQD 663
 PASTKKVELYAVITGHGSD------ENGCGEFCVTSHHFVINAAHNNTHVFDSAGTALGCAMRVKEGAVPNEHGTWLYGRGGWCDGLQVNPWRVDVTKQLNMNRSESNTVVYFGLFNGRD 644
 PPSTRKVELLATITGHGSD------ENGCGEFCVTSHHFLINGVHNNSRVFDSAGSILGCALRVKEGAVPNEHGTWLYGRGGWCDGLQVDPWRTDVTEQVDLSGLESNTIIYFGLFEGKD 644
 PASTKKVELYAIITAHGSD------ENFCGEFCVTSHNFLINRSINNTLVFESAGTPLGCAMRVPEGAVPNEHGTWLYGRAGWCDGLQVDPWRTDITSQLDLSGT--NSVLYFGLFEGRN 651
 PAWAKKVEFSTLVTGHGFN-----DSKSCAEFCNTVHHVTVNGNDF-TLSSPVTDNPLGCFEQVKDGVVPNQSGTWVYGRNNWCPGQGVKLWNSDLSAAATG--PGPHTLTYKALVDGQD 615
 PTNTEKAYLRTTISGWGHAKPYDAGSRGCAEWCFRTHTIAI----NNANTFQHQLGALGCSANPINN---QSPGNWAPDRAGWCPGMAVPTRIDVLNNSLTG-----STFSYEYKFQSW- 282
 PNPKTD-----PGNILMYSYLVFYQ-------------- 662
 PHPSQK-----PGYITMSSFLVFYK-------------- 672
 PNPSHD-----PGYIVMFSYLVFYK-------------- 683
 PNPAQQ-----PGYIIMTSSLVFYK-------------- 664
 PDPAQE-----PGYIIMSSFLIFYK-------------- 664
 PDPKTN-----PGYIIMYSFLVFYK-------------- 671
 HLSKLEDGAERDASIHMTSWLVYYAERGAALPSKPNVKQ 654
 ----TNNGTNGDAFYAISSFVIAKSNTPISAPVVTN--- 314
Zebrafish
Turbot
Atlantic salmon
Nile tilapia
Medaka
Common carp
D. radio
E. miri.
Zebrafish
Turbot
Atlantic salmon
Nile tilapia
Medaka
Common carp
D. radio
E. miri.
Zebrafish
Turbot
Atlantic salmon
Nile tilapia
Medaka
Common carp
D. radio
E. miri.
＊
Zebrafish
Turbot
Atlantic salmon
Nile tilapia
Medaka
Common carp
D. radio
E. miri.
Zebrafish
Turbot
Atlantic salmon
Nile tilapia
Medaka
Common carp
D. radio
E. miri.
＊
Zebrafish
Turbot
Atlantic salmon
Nile tilapia
Medaka
Common carp
D. radio
E. miri.
Zebrafish
Turbot
Atlantic salmon
Nile tilapia
Medaka
Common carp
D. radio
E. miri.
Figure S2 Structure-based multiple alignment of amino acid sequences of Ngly2 from zebrafish, turbot (GenBank: AWP15675.1), atlantic salmon (NP_001167083.1), nile tilapia (XP_003450330.1), medaka (XP_004078437.1), common carp (XP_042597645.1), Deinococcus radiodurans (UniProt: Q9RYl7) and Elizabethkingia miricola (UniProt: P21163). Labeled secondary elements (α-helices, columns, β-sheets, arrows, random coil, solid lines, disordered regions, broken lines) are illustrated. Domain regions are highlighted on the sequences based on the structure of Ngly2, as shown in Figure 1D and 3A. The catalytic residues involved in the PNGase activity (Asp421 and Glu555 in zebrafish Ngly2) are shown in bold red, with an asterisk displayed above each residue. The histidine residues located in the active site are highlighted in yellow. Potential N-glycosylation sites in zebrafish Ngly2 are indicated by bold letters (N). Putative signal sequence of Zebrafish Ngly2, 1-23 amino acid region, is predicted by SignalP-6.0 (https://services.healthtech.dtu.dk/services/SignalP-6.0/).

## Slide 3
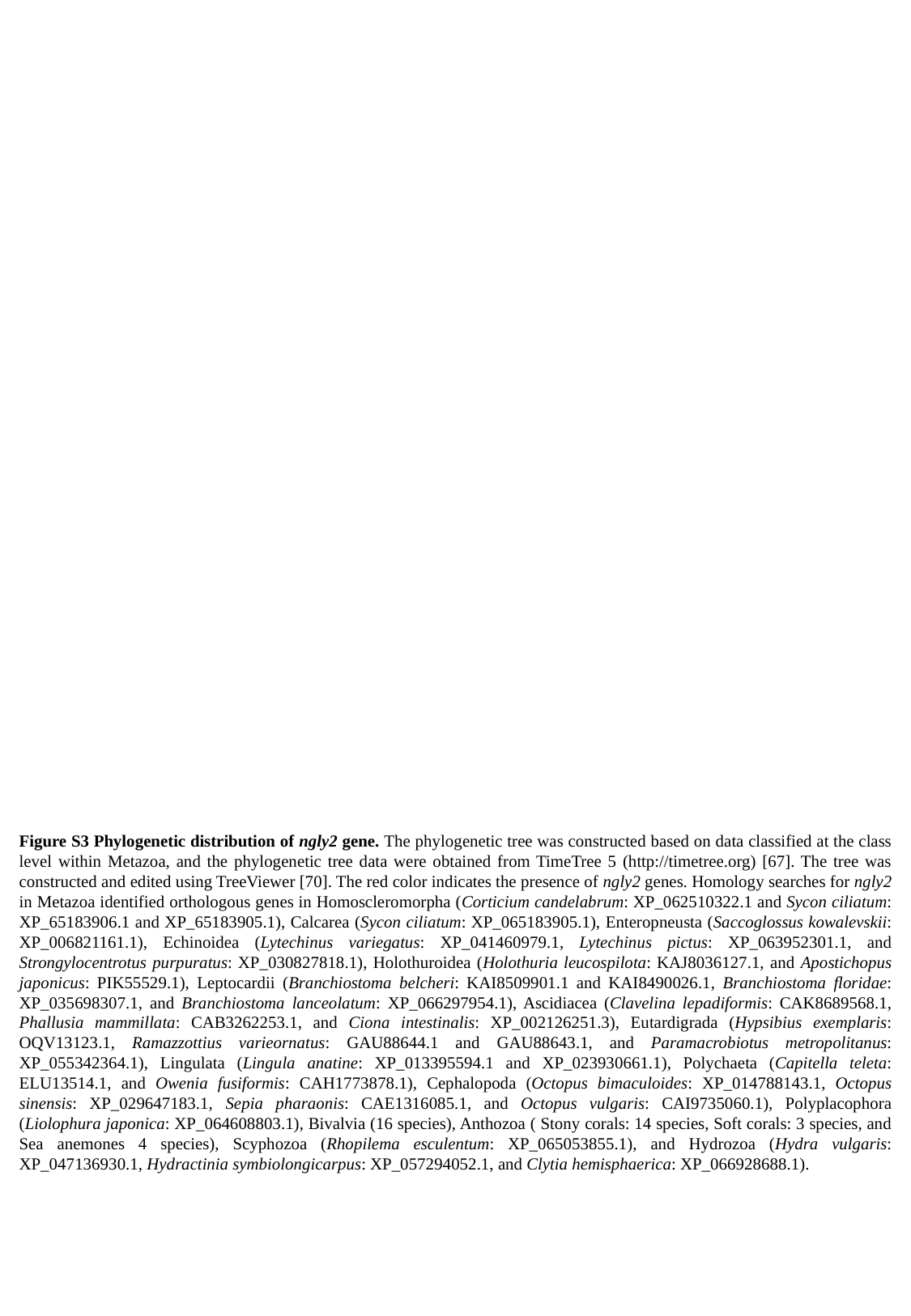

Figure S3 Phylogenetic distribution of ngly2 gene. The phylogenetic tree was constructed based on data classified at the class level within Metazoa, and the phylogenetic tree data were obtained from TimeTree 5 (http://timetree.org) [67]. The tree was constructed and edited using TreeViewer [70]. The red color indicates the presence of ngly2 genes. Homology searches for ngly2 in Metazoa identified orthologous genes in Homoscleromorpha (Corticium candelabrum: XP_062510322.1 and Sycon ciliatum: XP_65183906.1 and XP_65183905.1), Calcarea (Sycon ciliatum: XP_065183905.1), Enteropneusta (Saccoglossus kowalevskii: XP_006821161.1), Echinoidea (Lytechinus variegatus: XP_041460979.1, Lytechinus pictus: XP_063952301.1, and Strongylocentrotus purpuratus: XP_030827818.1), Holothuroidea (Holothuria leucospilota: KAJ8036127.1, and Apostichopus japonicus: PIK55529.1), Leptocardii (Branchiostoma belcheri: KAI8509901.1 and KAI8490026.1, Branchiostoma floridae: XP_035698307.1, and Branchiostoma lanceolatum: XP_066297954.1), Ascidiacea (Clavelina lepadiformis: CAK8689568.1, Phallusia mammillata: CAB3262253.1, and Ciona intestinalis: XP_002126251.3), Eutardigrada (Hypsibius exemplaris: OQV13123.1, Ramazzottius varieornatus: GAU88644.1 and GAU88643.1, and Paramacrobiotus metropolitanus: XP_055342364.1), Lingulata (Lingula anatine: XP_013395594.1 and XP_023930661.1), Polychaeta (Capitella teleta: ELU13514.1, and Owenia fusiformis: CAH1773878.1), Cephalopoda (Octopus bimaculoides: XP_014788143.1, Octopus sinensis: XP_029647183.1, Sepia pharaonis: CAE1316085.1, and Octopus vulgaris: CAI9735060.1), Polyplacophora (Liolophura japonica: XP_064608803.1), Bivalvia (16 species), Anthozoa ( Stony corals: 14 species, Soft corals: 3 species, and Sea anemones 4 species), Scyphozoa (Rhopilema esculentum: XP_065053855.1), and Hydrozoa (Hydra vulgaris: XP_047136930.1, Hydractinia symbiolongicarpus: XP_057294052.1, and Clytia hemisphaerica: XP_066928688.1).

## Slide 4
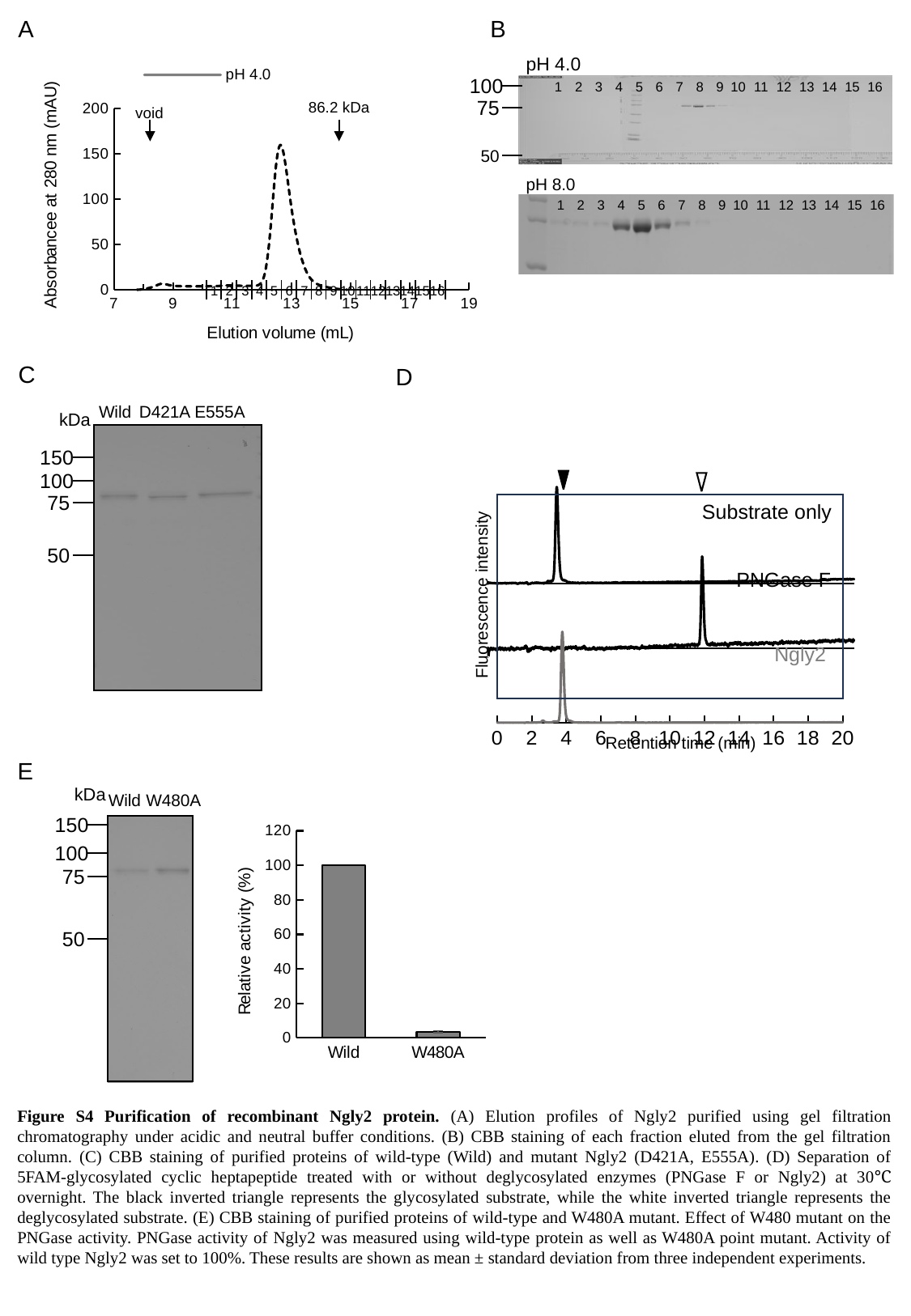

A
B
pH 4.0
1
2
3
4
5
6
7
8
9
10
11
12
13
14
15
16
### Chart
| Category | pH 4.0 | pH 8.0 |
|---|---|---|86.2 kDa
1
2
3
4
5
6
7
8
9
10
11
12
13
14
15
16
void
100
75
50
pH 8.0
1
2
3
4
5
6
7
8
9
10
11
12
13
14
15
16
C
D
Wild
D421A
E555A
kDa
150
100
### Chart
| Category | |
|---|---|Substrate only
### Chart
| Category | |
|---|---|PNGase F
Fluorescence intensity
### Chart
| Category | |
|---|---|Ngly2
Retention time (min)
75
50
E
kDa
Wild
W480A
150
100
75
50
### Chart
| Category | |
|---|---|
| Wild | 100.0 |
| W480A | 3.6162348192993314 |Figure S4 Purification of recombinant Ngly2 protein. (A) Elution profiles of Ngly2 purified using gel filtration chromatography under acidic and neutral buffer conditions. (B) CBB staining of each fraction eluted from the gel filtration column. (C) CBB staining of purified proteins of wild-type (Wild) and mutant Ngly2 (D421A, E555A). (D) Separation of 5FAM-glycosylated cyclic heptapeptide treated with or without deglycosylated enzymes (PNGase F or Ngly2) at 30℃ overnight. The black inverted triangle represents the glycosylated substrate, while the white inverted triangle represents the deglycosylated substrate. (E) CBB staining of purified proteins of wild-type and W480A mutant. Effect of W480 mutant on the PNGase activity. PNGase activity of Ngly2 was measured using wild-type protein as well as W480A point mutant. Activity of wild type Ngly2 was set to 100%. These results are shown as mean ± standard deviation from three independent experiments.

## Slide 5
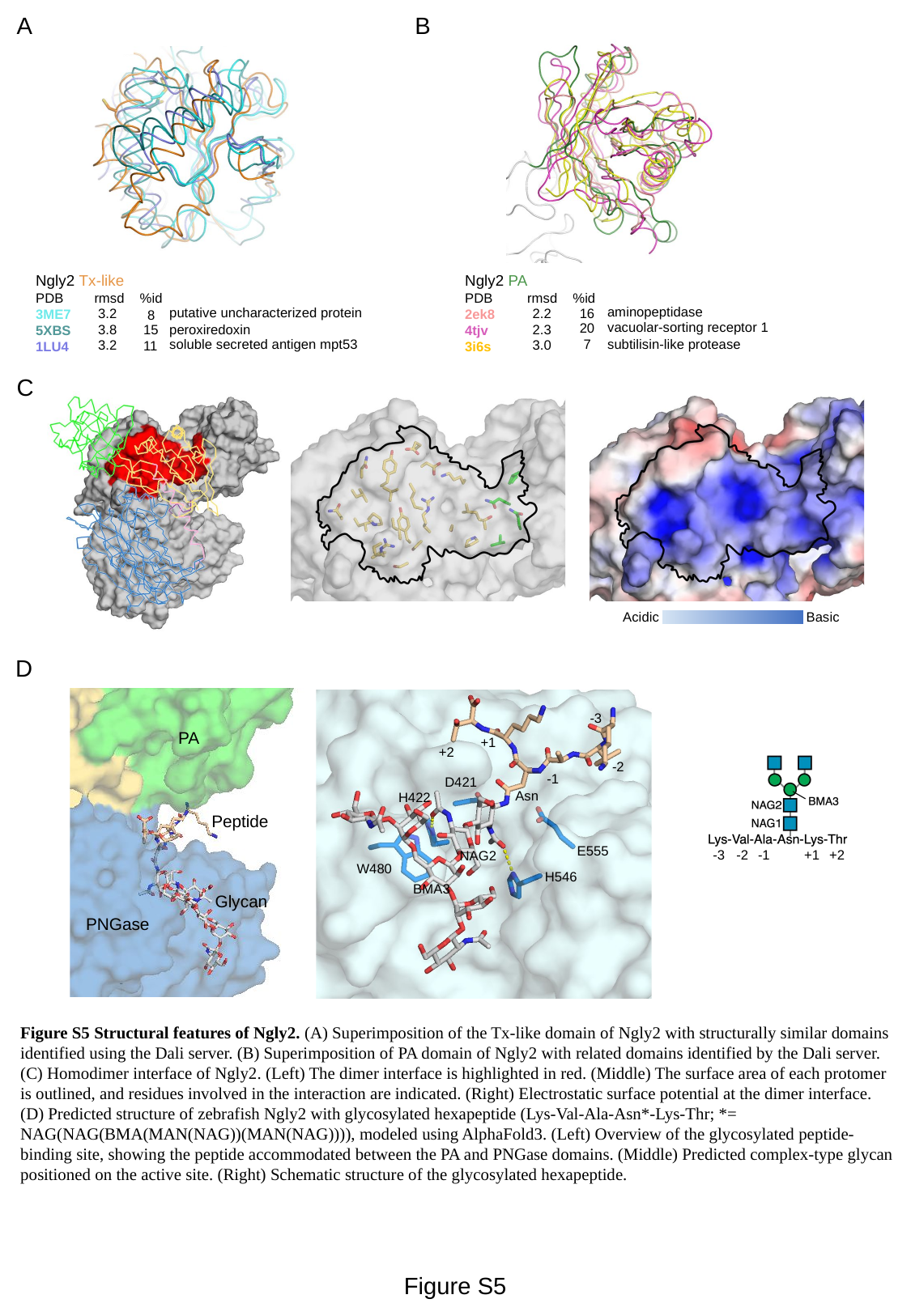

B
A
Ngly2 Tx-like
PDB rmsd %id
3ME7
5XBS
1LU4
putative uncharacterized protein
3.2
8
3.8
15
peroxiredoxin
soluble secreted antigen mpt53
3.2
11
Ngly2 PA
PDB rmsd %id
2ek8
4tjv
3i6s
2.2
16
20
2.3
7
3.0
aminopeptidase
vacuolar-sorting receptor 1
subtilisin-like protease
C
Acidic
Basic
D
PA
PNGase
Peptide
Glycan
-3
+1
+2
-2
-1
D421
Asn
H422
E555
NAG2
W480
H546
BMA3
-3
-2
-1
+1
+2
Figure S5 Structural features of Ngly2. (A) Superimposition of the Tx-like domain of Ngly2 with structurally similar domains identified using the Dali server. (B) Superimposition of PA domain of Ngly2 with related domains identified by the Dali server. (C) Homodimer interface of Ngly2. (Left) The dimer interface is highlighted in red. (Middle) The surface area of each protomer is outlined, and residues involved in the interaction are indicated. (Right) Electrostatic surface potential at the dimer interface. (D) Predicted structure of zebrafish Ngly2 with glycosylated hexapeptide (Lys-Val-Ala-Asn*-Lys-Thr; *= NAG(NAG(BMA(MAN(NAG))(MAN(NAG)))), modeled using AlphaFold3. (Left) Overview of the glycosylated peptide-binding site, showing the peptide accommodated between the PA and PNGase domains. (Middle) Predicted complex-type glycan positioned on the active site. (Right) Schematic structure of the glycosylated hexapeptide.
Figure S5

## Slide 6
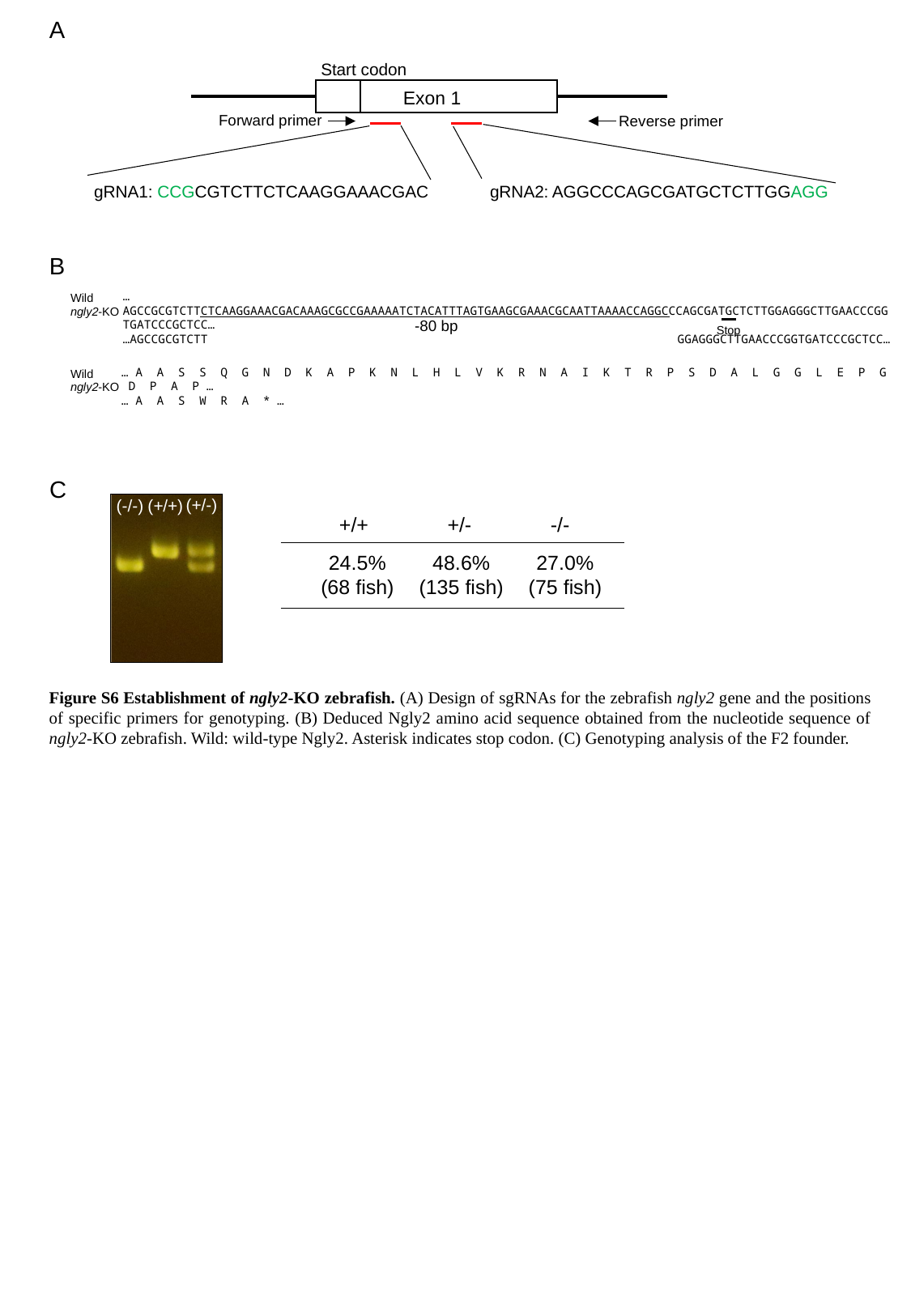

A
Start codon
Exon 1
Forward primer
Reverse primer
gRNA1: CCGCGTCTTCTCAAGGAAACGAC
gRNA2: AGGCCCAGCGATGCTCTTGGAGG
B
…AGCCGCGTCTTCTCAAGGAAACGACAAAGCGCCGAAAAATCTACATTTAGTGAAGCGAAACGCAATTAAAACCAGGCCCAGCGATGCTCTTGGAGGGCTTGAACCCGGTGATCCCGCTCC…
…AGCCGCGTCTT　　　　　　　　　　　　　　　　　　　　　　　　　　　　　　　　　　　　　　　 GGAGGGCTTGAACCCGGTGATCCCGCTCC…
Wild
ngly2-KO
-80 bp
Stop
… A A S S Q G N D K A P K N L H L V K R N A I K T R P S D A L G G L E P G D P A P …
… A A S W R A * …
Wild
ngly2-KO
C
(+/-)
(+/+)
(-/-)
+/+ +/- -/-
24.5%
(68 fish)
48.6%
(135 fish)
27.0%
(75 fish)
Figure S6 Establishment of ngly2-KO zebrafish. (A) Design of sgRNAs for the zebrafish ngly2 gene and the positions of specific primers for genotyping. (B) Deduced Ngly2 amino acid sequence obtained from the nucleotide sequence of ngly2-KO zebrafish. Wild: wild-type Ngly2. Asterisk indicates stop codon. (C) Genotyping analysis of the F2 founder.

## Slide 7
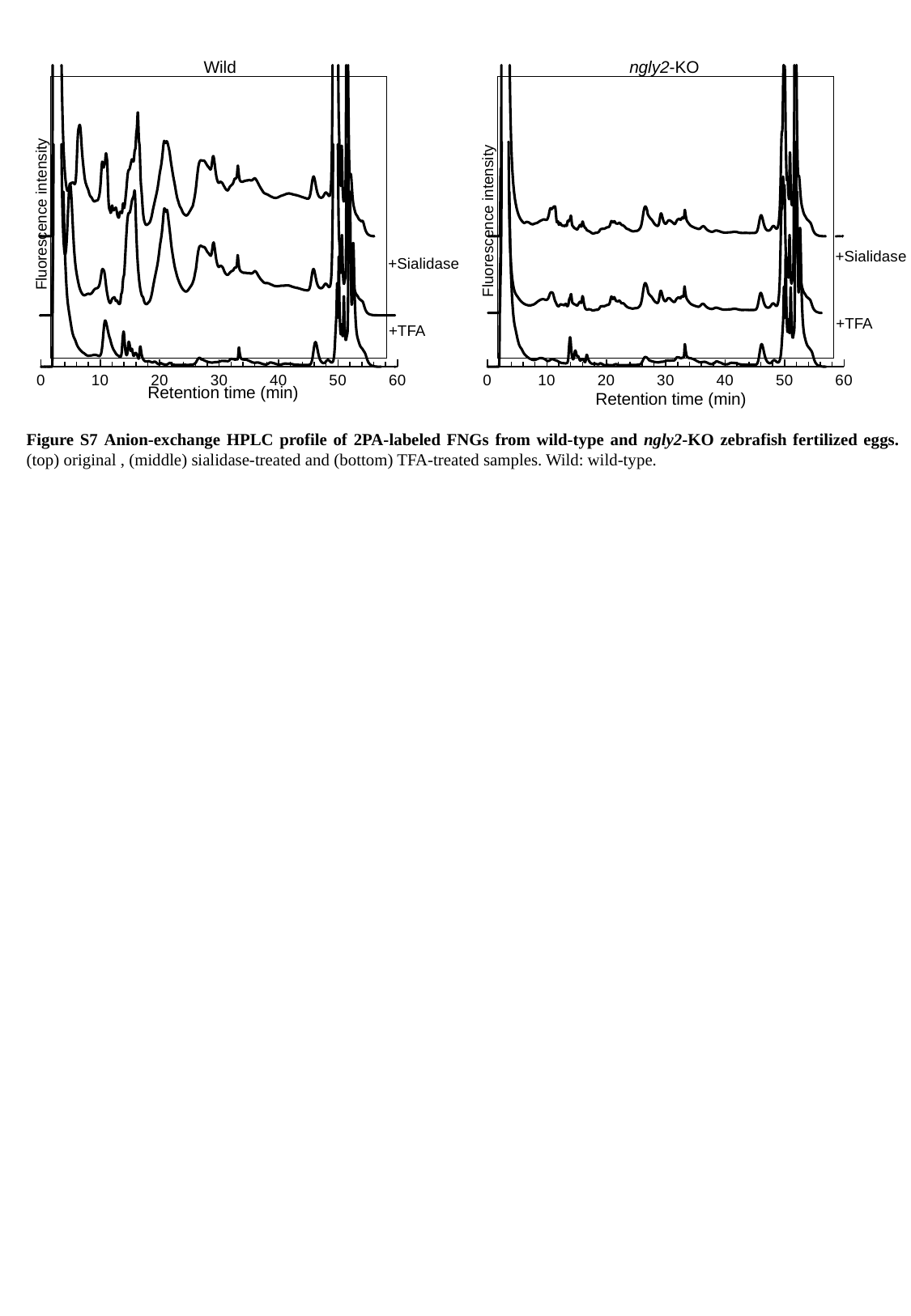

ngly2-KO
Wild
### Chart
| Category | |
|---|---|
### Chart
| Category | |
|---|---|
### Chart
| Category | |
|---|---|
### Chart
| Category | |
|---|---|
### Chart
| Category | |
|---|---|
### Chart
| Category | |
|---|---|Fluorescence intensity
Fluorescence intensity
+Sialidase
+Sialidase
+TFA
+TFA
Retention time (min)
Retention time (min)
Figure S7 Anion-exchange HPLC profile of 2PA-labeled FNGs from wild-type and ngly2-KO zebrafish fertilized eggs. (top) original , (middle) sialidase-treated and (bottom) TFA-treated samples. Wild: wild-type.

## Slide 8
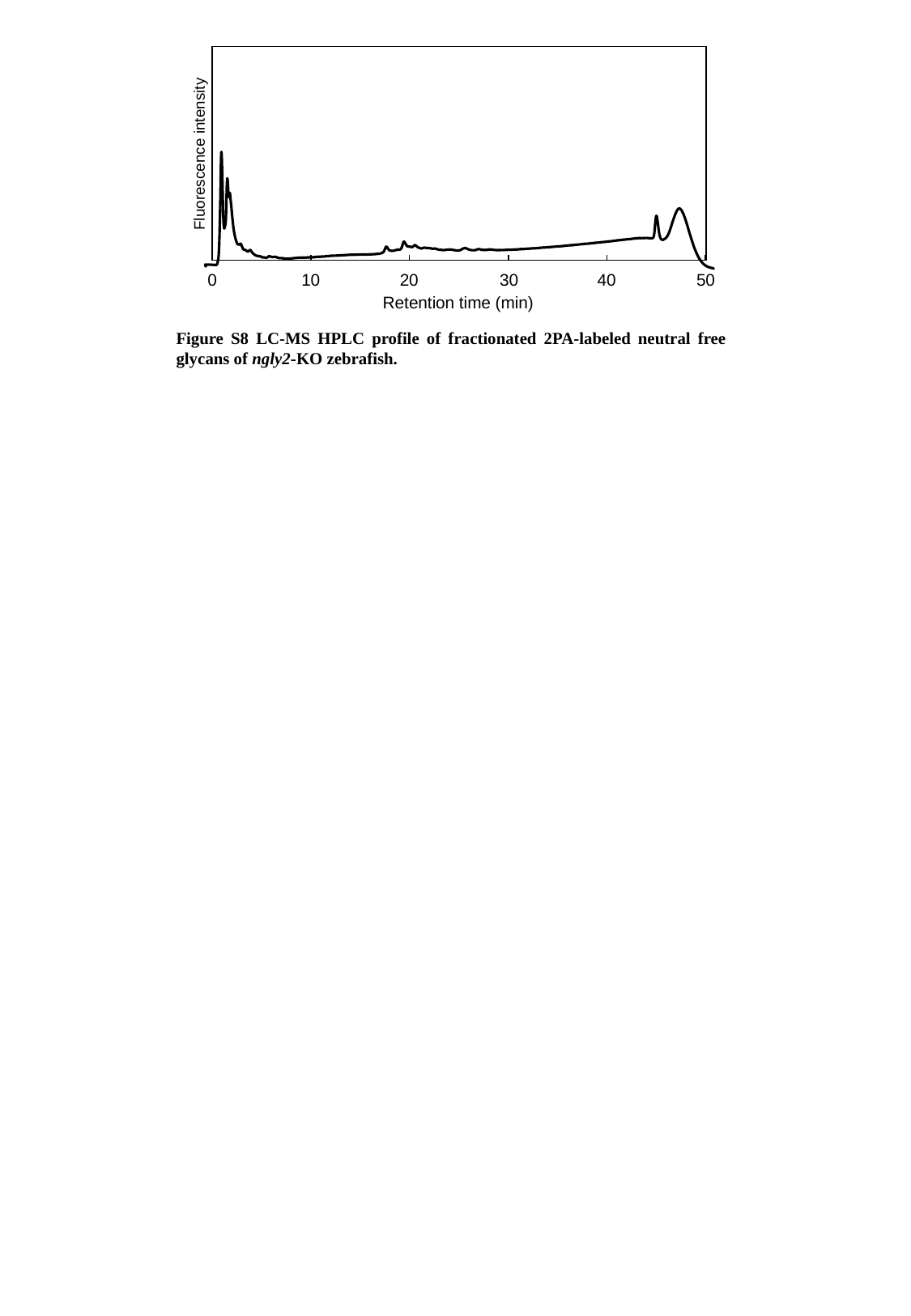

### Chart
| Category | |
|---|---|
Fluorescence intensity
0
10
20
30
40
50
Retention time (min)
Figure S8 LC-MS HPLC profile of fractionated 2PA-labeled neutral free glycans of ngly2-KO zebrafish.

## Slide 9
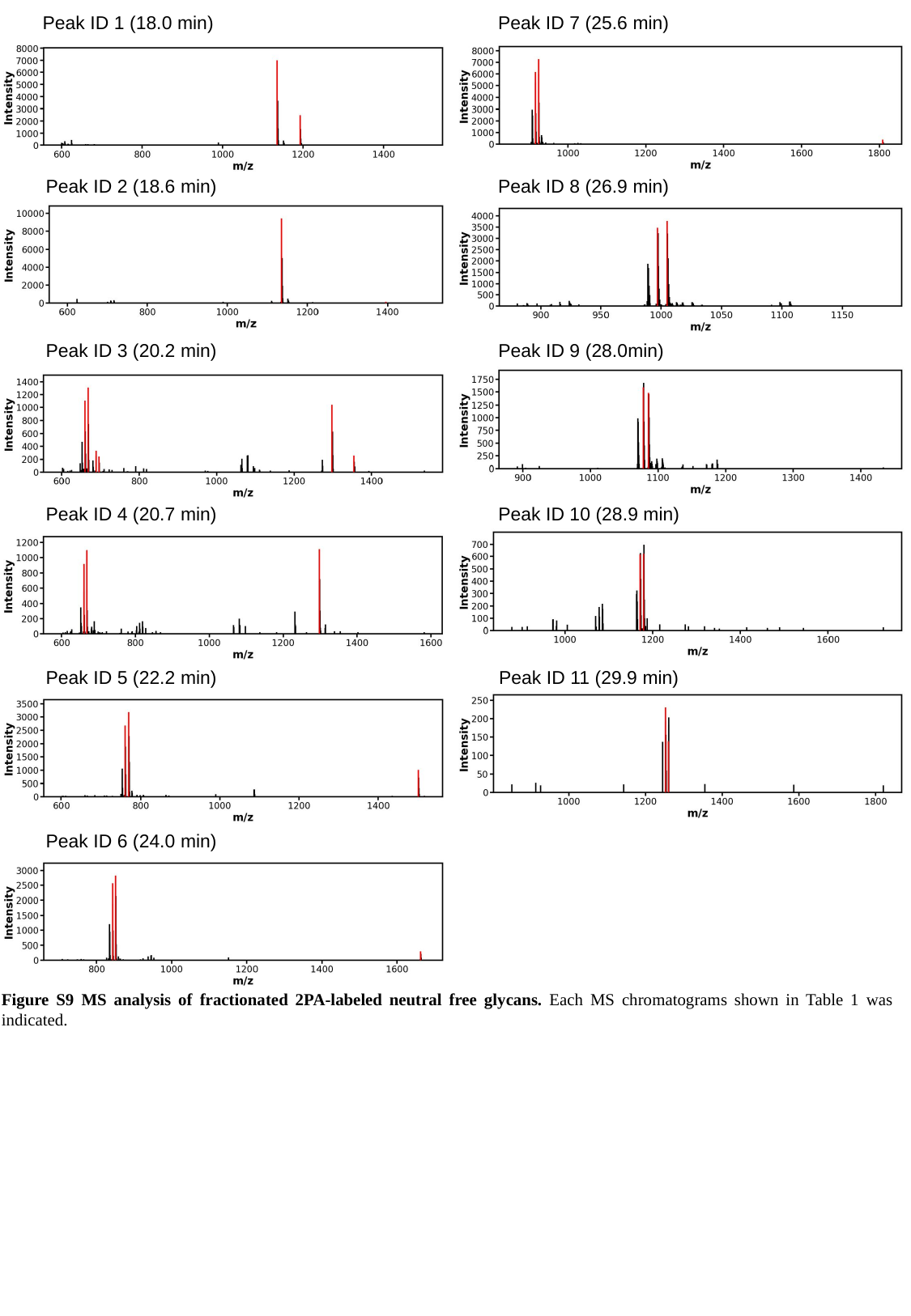

Peak ID 1 (18.0 min)
Peak ID 7 (25.6 min)
Peak ID 2 (18.6 min)
Peak ID 8 (26.9 min)
Peak ID 3 (20.2 min)
Peak ID 9 (28.0min)
Peak ID 4 (20.7 min)
Peak ID 10 (28.9 min)
Peak ID 5 (22.2 min)
Peak ID 11 (29.9 min)
Peak ID 6 (24.0 min)
Figure S9 MS analysis of fractionated 2PA-labeled neutral free glycans. Each MS chromatograms shown in Table 1 was indicated.

## Slide 10
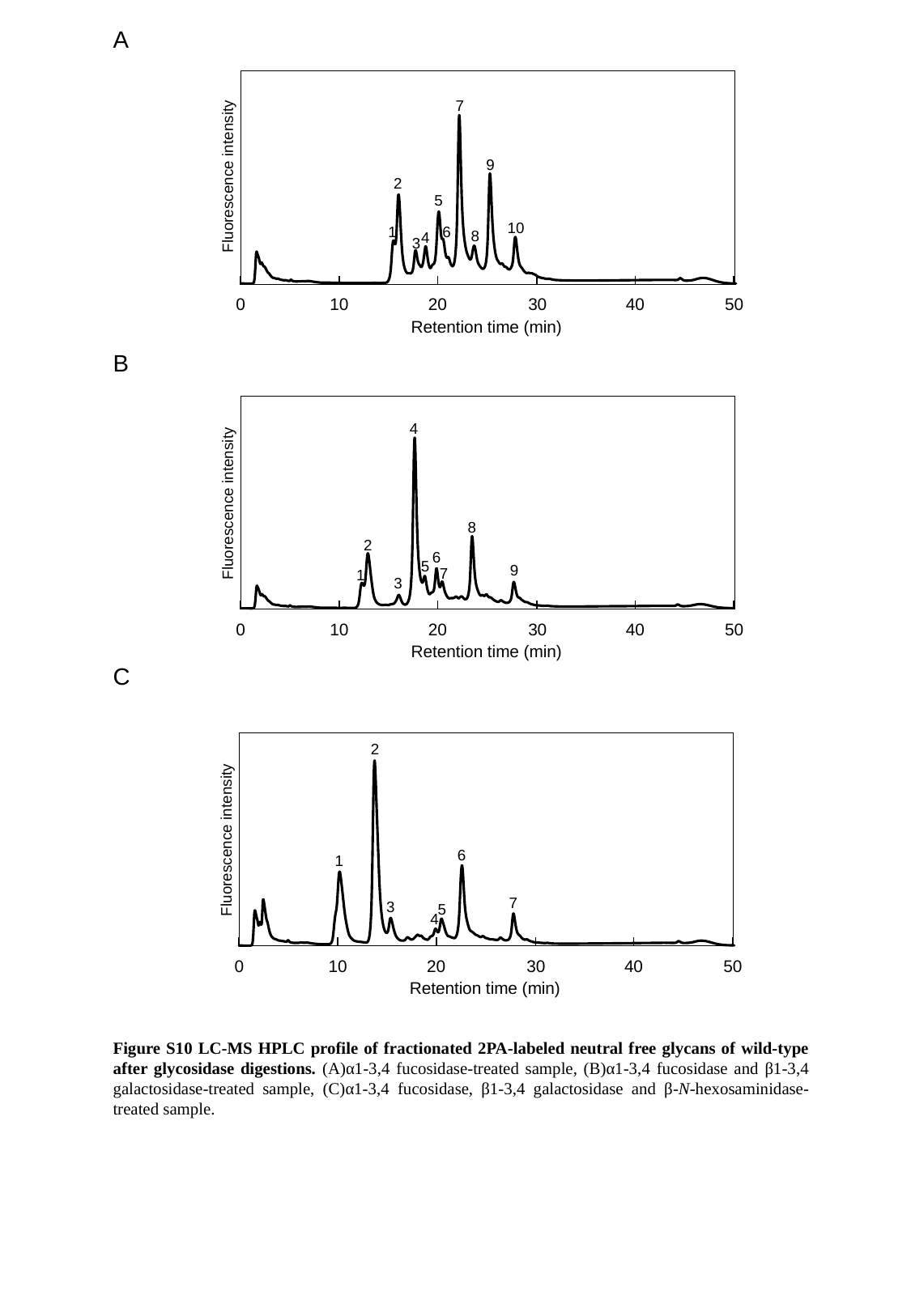

A
 Fluorescence intensity
0
10
20
30
40
50
Retention time (min)
### Chart
| Category | |
|---|---|7
9
2
5
10
6
1
8
4
3
B
### Chart
| Category | |
|---|---|
Fluorescence intensity
0
10
20
30
40
50
Retention time (min)
4
8
2
6
5
9
7
1
3
C
### Chart
| Category | |
|---|---|
Fluorescence intensity
0
10
20
30
40
50
Retention time (min)
2
6
1
7
3
5
4
Figure S10 LC-MS HPLC profile of fractionated 2PA-labeled neutral free glycans of wild-type after glycosidase digestions. (A)α1-3,4 fucosidase-treated sample, (B)α1-3,4 fucosidase and β1-3,4 galactosidase-treated sample, (C)α1-3,4 fucosidase, β1-3,4 galactosidase and β-N-hexosaminidase-treated sample.

## Slide 11
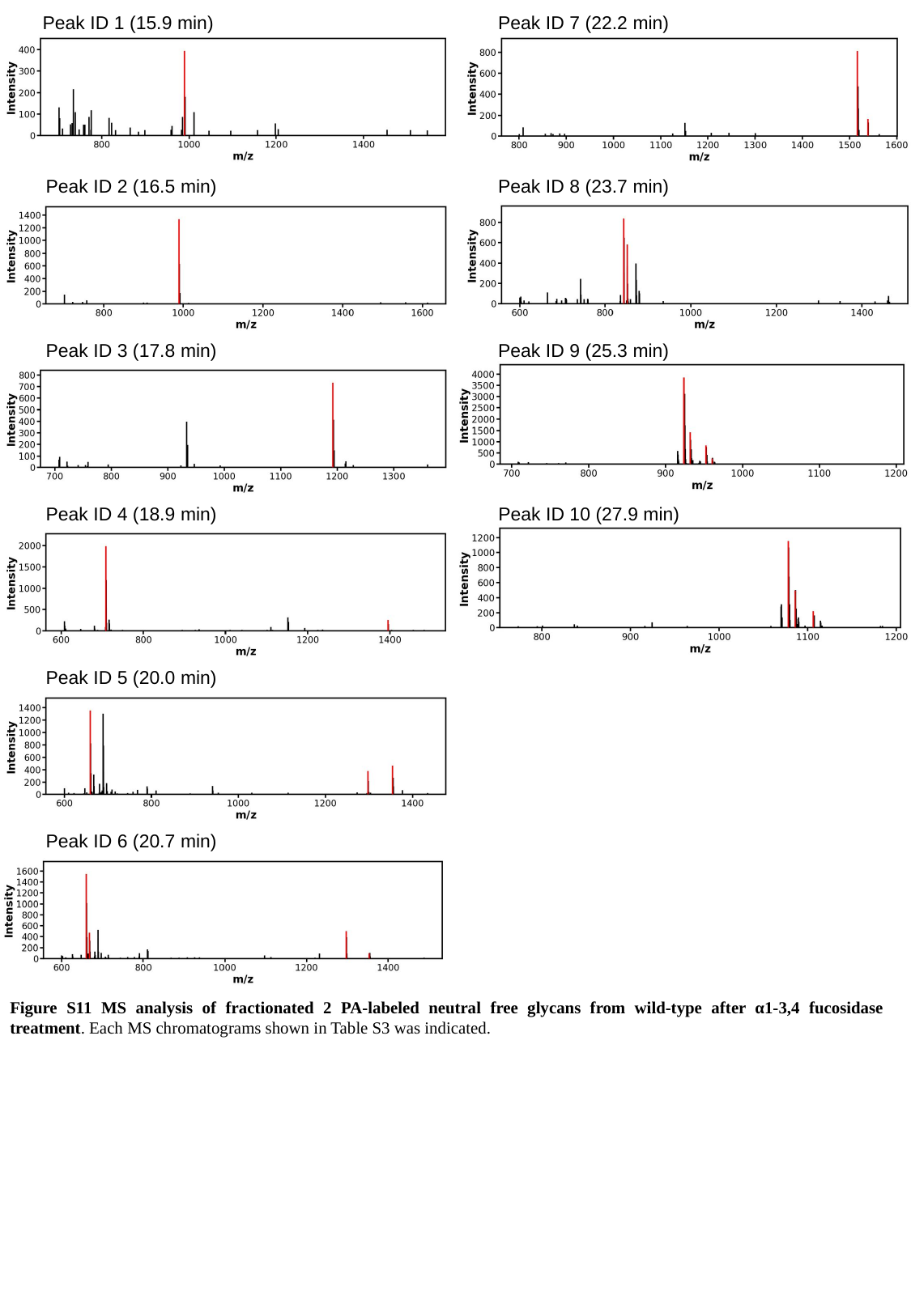

Peak ID 1 (15.9 min)
Peak ID 7 (22.2 min)
Peak ID 2 (16.5 min)
Peak ID 8 (23.7 min)
Peak ID 3 (17.8 min)
Peak ID 9 (25.3 min)
Peak ID 4 (18.9 min)
Peak ID 10 (27.9 min)
Peak ID 5 (20.0 min)
Peak ID 6 (20.7 min)
Figure S11 MS analysis of fractionated 2 PA-labeled neutral free glycans from wild-type after α1-3,4 fucosidase treatment. Each MS chromatograms shown in Table S3 was indicated.

## Slide 12
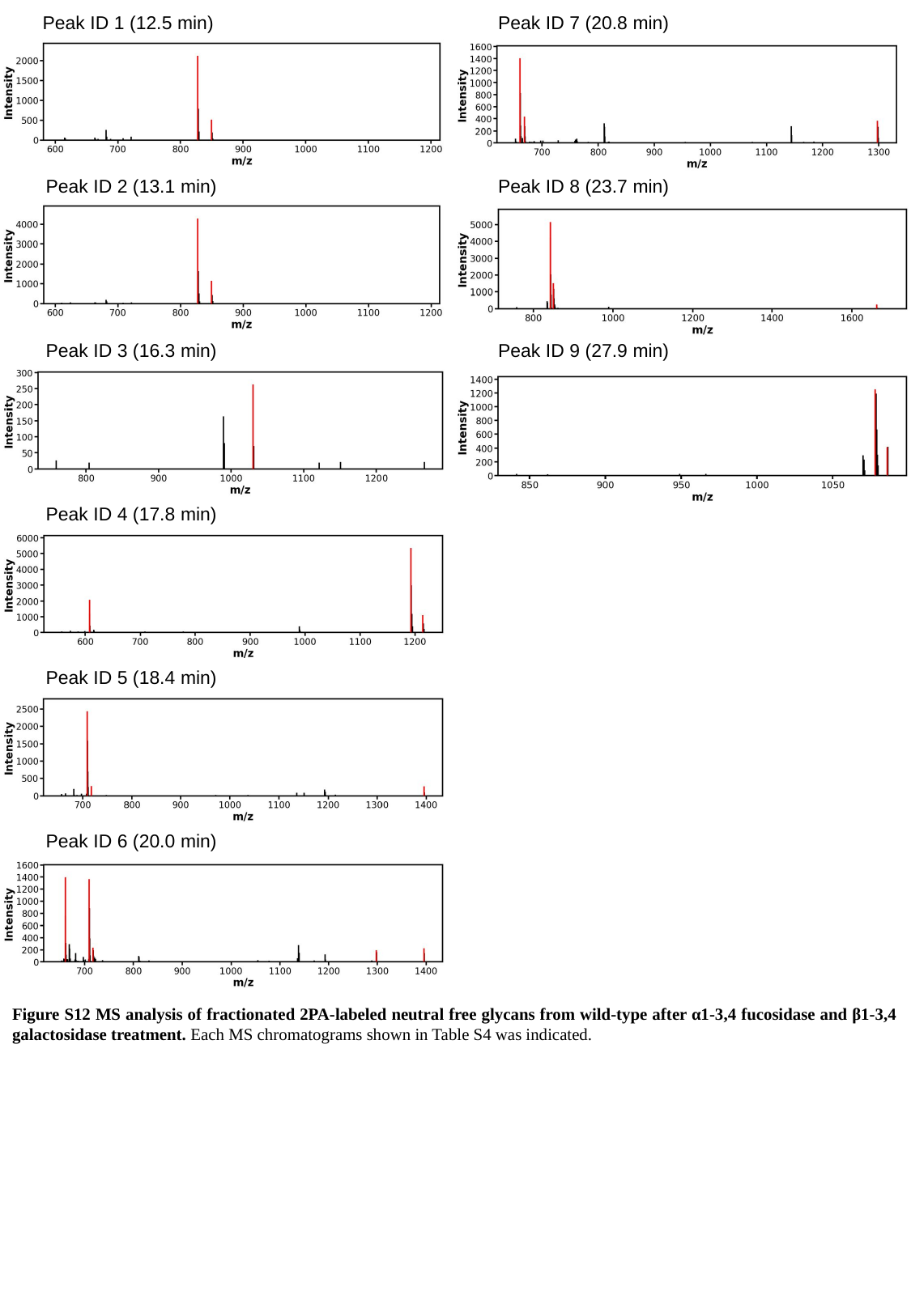

Peak ID 1 (12.5 min)
Peak ID 7 (20.8 min)
Peak ID 2 (13.1 min)
Peak ID 8 (23.7 min)
Peak ID 3 (16.3 min)
Peak ID 9 (27.9 min)
Peak ID 4 (17.8 min)
Peak ID 5 (18.4 min)
Peak ID 6 (20.0 min)
Figure S12 MS analysis of fractionated 2PA-labeled neutral free glycans from wild-type after α1-3,4 fucosidase and β1-3,4 galactosidase treatment. Each MS chromatograms shown in Table S4 was indicated.

## Slide 13
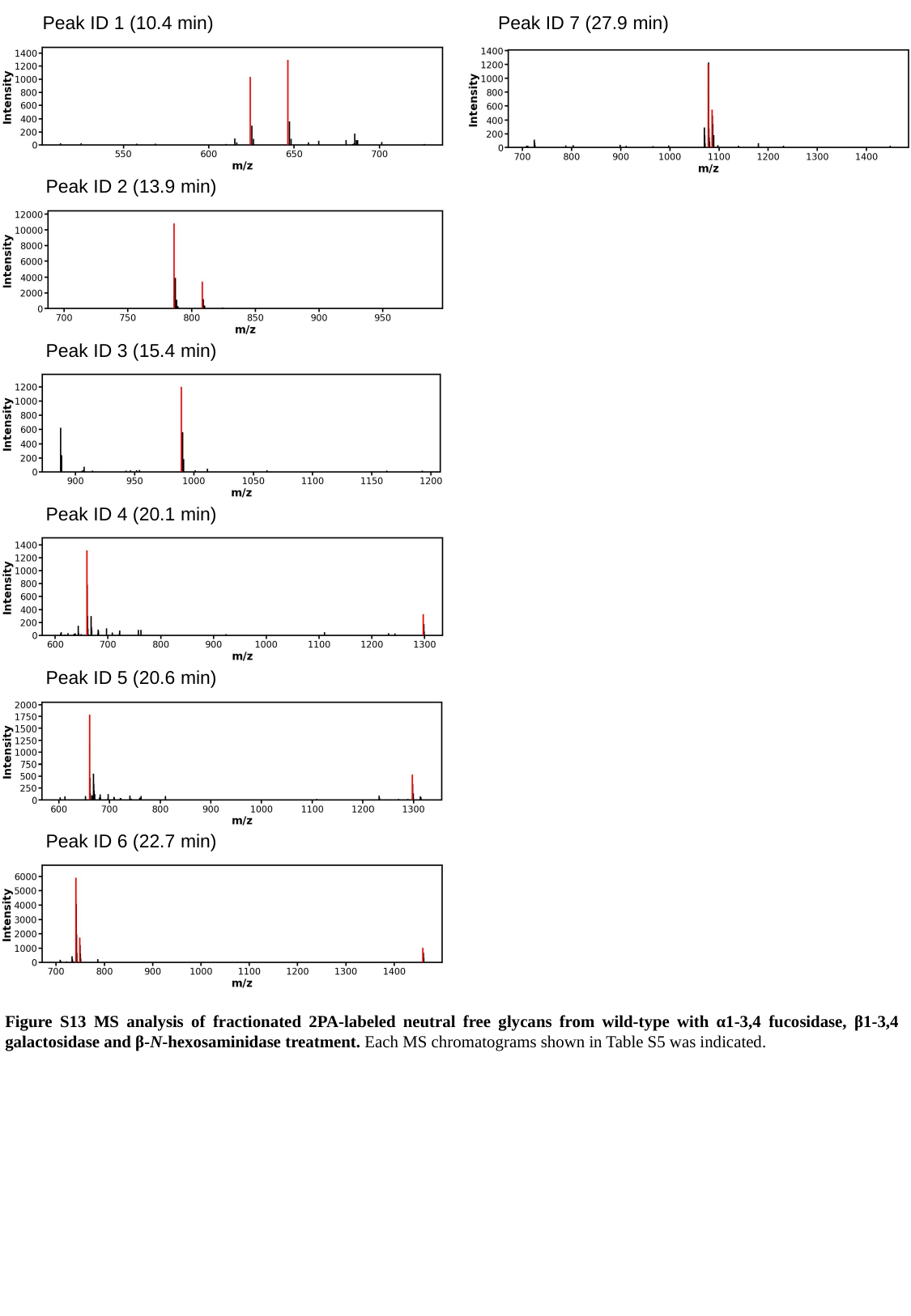

Peak ID 1 (10.4 min)
Peak ID 7 (27.9 min)
Peak ID 2 (13.9 min)
Peak ID 3 (15.4 min)
Peak ID 4 (20.1 min)
Peak ID 5 (20.6 min)
Peak ID 6 (22.7 min)
Figure S13 MS analysis of fractionated 2PA-labeled neutral free glycans from wild-type with α1-3,4 fucosidase, β1-3,4 galactosidase and β-N-hexosaminidase treatment. Each MS chromatograms shown in Table S5 was indicated.

## Slide 14
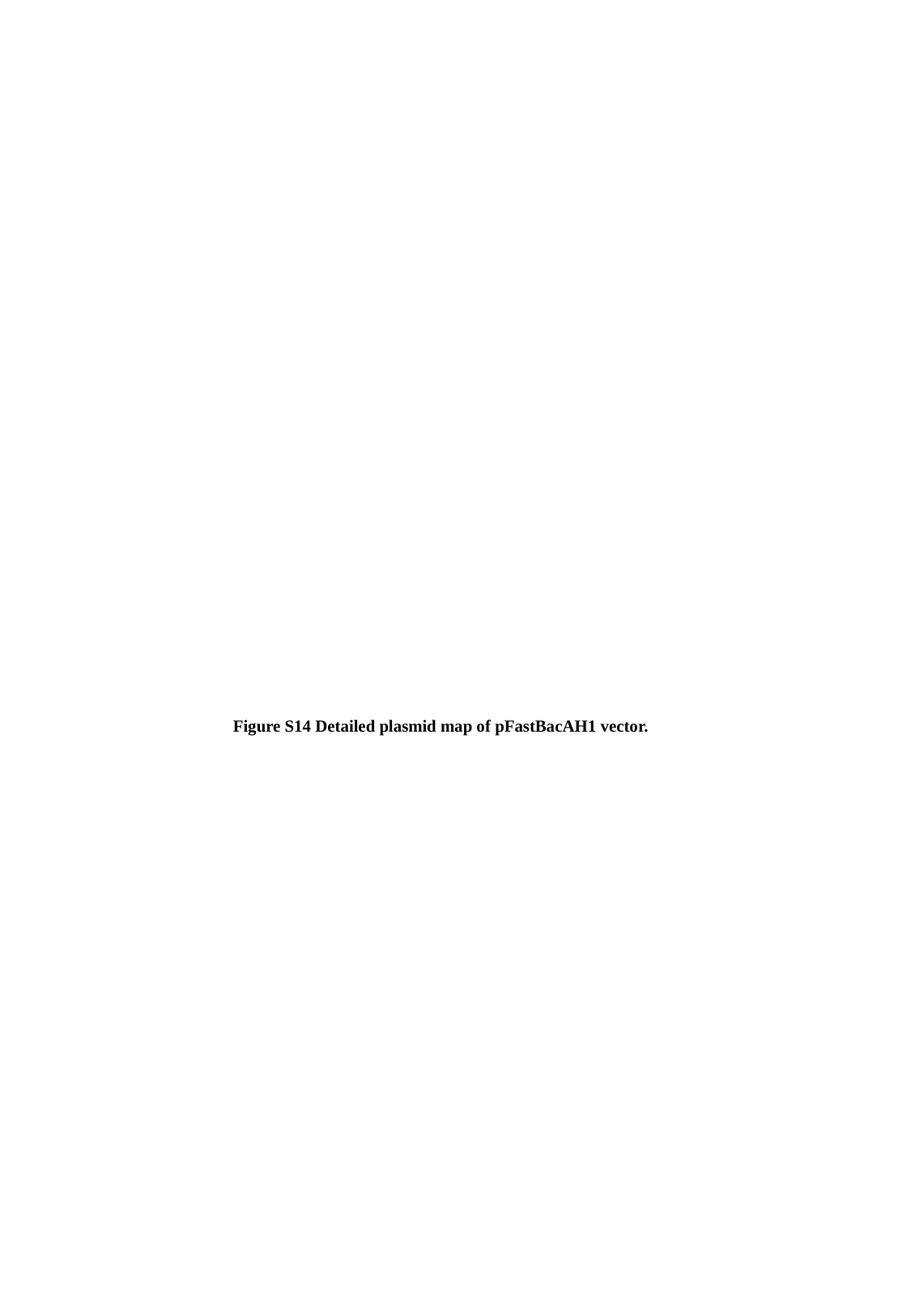

Figure S14 Detailed plasmid map of pFastBacAH1 vector.

## Slide 15
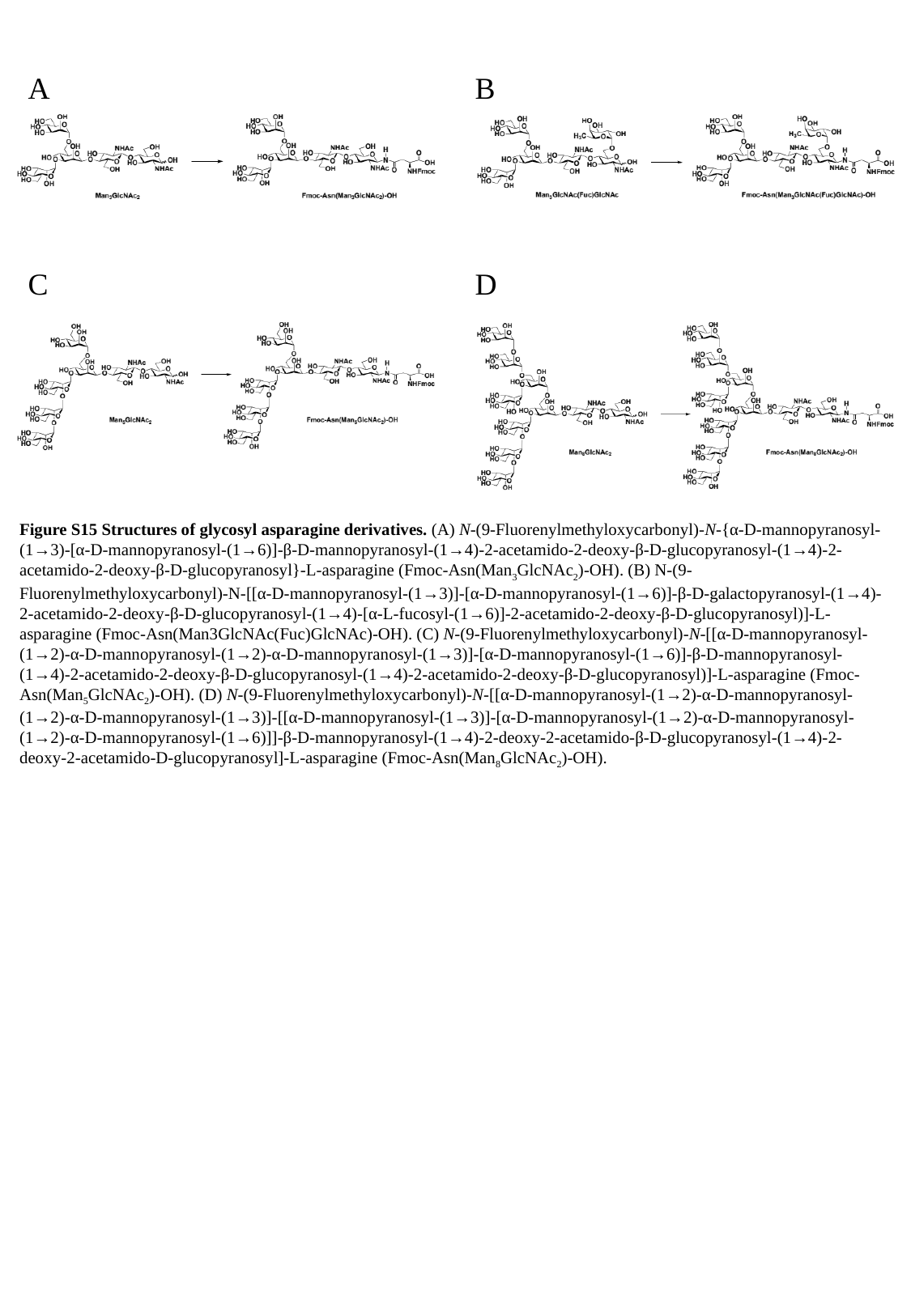

A
B
C
D
Figure S15 Structures of glycosyl asparagine derivatives. (A) N-(9-Fluorenylmethyloxycarbonyl)-N-{α-D-mannopyranosyl-(1→3)-[α-D-mannopyranosyl-(1→6)]-β-D-mannopyranosyl-(1→4)-2-acetamido-2-deoxy-β-D-glucopyranosyl-(1→4)-2-acetamido-2-deoxy-β-D-glucopyranosyl}-L-asparagine (Fmoc-Asn(Man3GlcNAc2)-OH). (B) N-(9-Fluorenylmethyloxycarbonyl)-N-[[α-D-mannopyranosyl-(1→3)]-[α-D-mannopyranosyl-(1→6)]-β-D-galactopyranosyl-(1→4)-2-acetamido-2-deoxy-β-D-glucopyranosyl-(1→4)-[α-L-fucosyl-(1→6)]-2-acetamido-2-deoxy-β-D-glucopyranosyl)]-L-asparagine (Fmoc-Asn(Man3GlcNAc(Fuc)GlcNAc)-OH). (C) N-(9-Fluorenylmethyloxycarbonyl)-N-[[α-D-mannopyranosyl-(1→2)-α-D-mannopyranosyl-(1→2)-α-D-mannopyranosyl-(1→3)]-[α-D-mannopyranosyl-(1→6)]-β-D-mannopyranosyl-(1→4)-2-acetamido-2-deoxy-β-D-glucopyranosyl-(1→4)-2-acetamido-2-deoxy-β-D-glucopyranosyl)]-L-asparagine (Fmoc-Asn(Man5GlcNAc2)-OH). (D) N-(9-Fluorenylmethyloxycarbonyl)-N-[[α-D-mannopyranosyl-(1→2)-α-D-mannopyranosyl-(1→2)-α-D-mannopyranosyl-(1→3)]-[[α-D-mannopyranosyl-(1→3)]-[α-D-mannopyranosyl-(1→2)-α-D-mannopyranosyl-(1→2)-α-D-mannopyranosyl-(1→6)]]-β-D-mannopyranosyl-(1→4)-2-deoxy-2-acetamido-β-D-glucopyranosyl-(1→4)-2-deoxy-2-acetamido-D-glucopyranosyl]-L-asparagine (Fmoc-Asn(Man8GlcNAc2)-OH).

## Slide 16
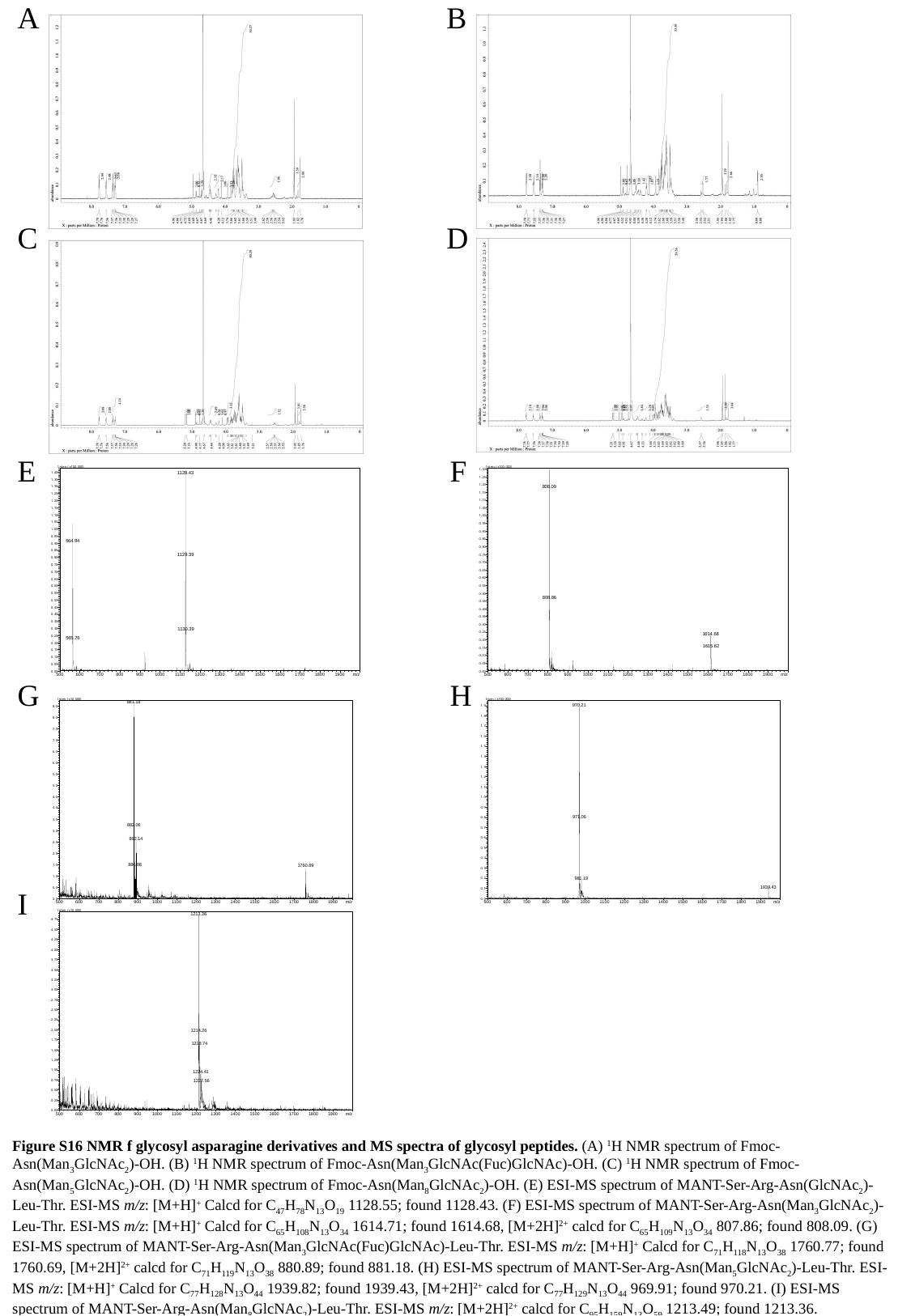

A
B
C
D
E
F
G
H
I
Figure S16 NMR f glycosyl asparagine derivatives and MS spectra of glycosyl peptides. (A) 1H NMR spectrum of Fmoc-Asn(Man3GlcNAc2)-OH. (B) 1H NMR spectrum of Fmoc-Asn(Man3GlcNAc(Fuc)GlcNAc)-OH. (C) 1H NMR spectrum of Fmoc-Asn(Man5GlcNAc2)-OH. (D) 1H NMR spectrum of Fmoc-Asn(Man8GlcNAc2)-OH. (E) ESI-MS spectrum of MANT-Ser-Arg-Asn(GlcNAc2)-Leu-Thr. ESI-MS m/z: [M+H]+ Calcd for C47H78N13O19 1128.55; found 1128.43. (F) ESI-MS spectrum of MANT-Ser-Arg-Asn(Man3GlcNAc2)-Leu-Thr. ESI-MS m/z: [M+H]+ Calcd for C65H108N13O34 1614.71; found 1614.68, [M+2H]2+ calcd for C65H109N13O34 807.86; found 808.09. (G) ESI-MS spectrum of MANT-Ser-Arg-Asn(Man3GlcNAc(Fuc)GlcNAc)-Leu-Thr. ESI-MS m/z: [M+H]+ Calcd for C71H118N13O38 1760.77; found 1760.69, [M+2H]2+ calcd for C71H119N13O38 880.89; found 881.18. (H) ESI-MS spectrum of MANT-Ser-Arg-Asn(Man5GlcNAc2)-Leu-Thr. ESI-MS m/z: [M+H]+ Calcd for C77H128N13O44 1939.82; found 1939.43, [M+2H]2+ calcd for C77H129N13O44 969.91; found 970.21. (I) ESI-MS spectrum of MANT-Ser-Arg-Asn(Man8GlcNAc2)-Leu-Thr. ESI-MS m/z: [M+2H]2+ calcd for C95H159N13O59 1213.49; found 1213.36.

## Slide 17
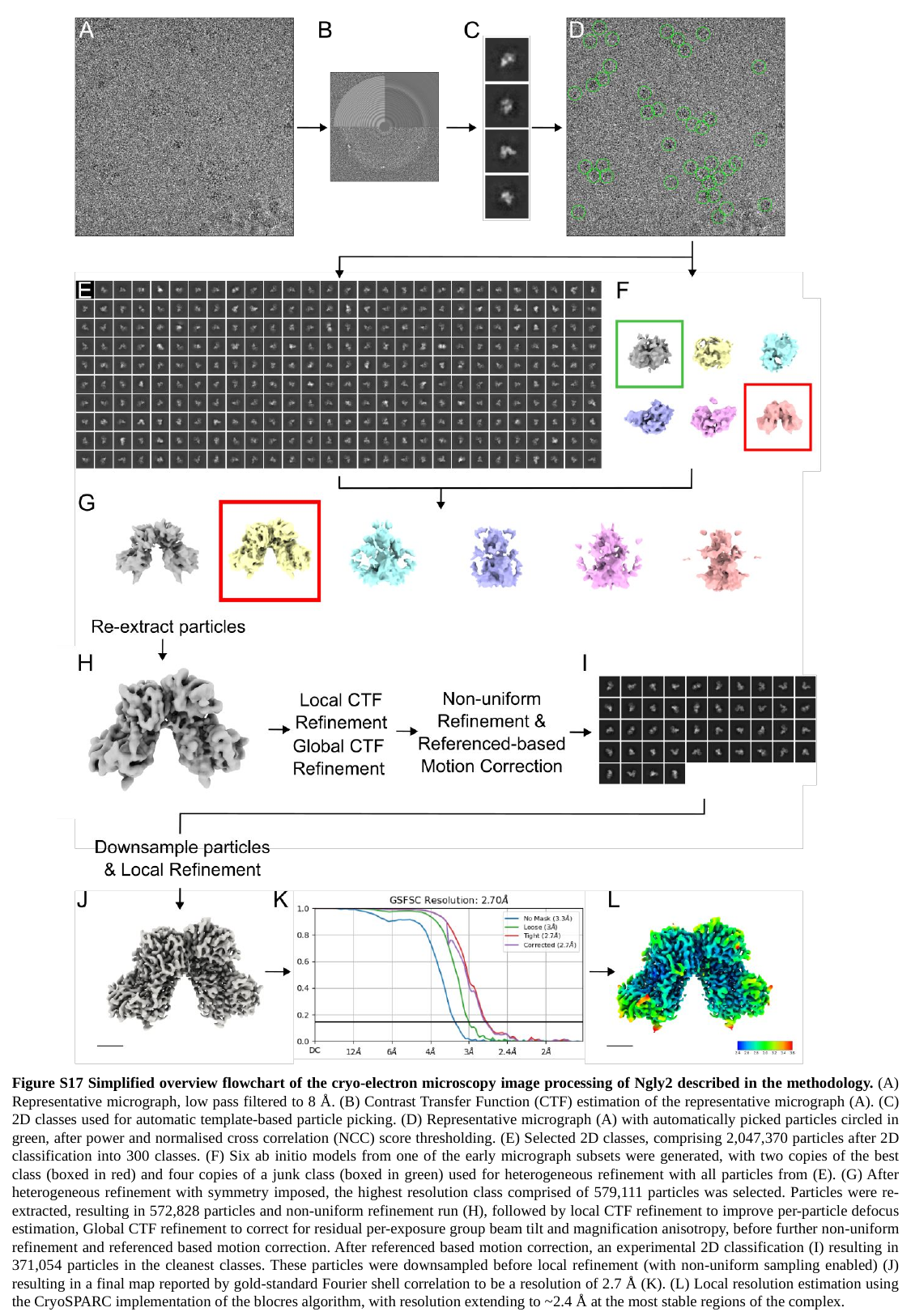

Figure S17 Simplified overview flowchart of the cryo-electron microscopy image processing of Ngly2 described in the methodology. (A) Representative micrograph, low pass filtered to 8 Å. (B) Contrast Transfer Function (CTF) estimation of the representative micrograph (A). (C) 2D classes used for automatic template-based particle picking. (D) Representative micrograph (A) with automatically picked particles circled in green, after power and normalised cross correlation (NCC) score thresholding. (E) Selected 2D classes, comprising 2,047,370 particles after 2D classification into 300 classes. (F) Six ab initio models from one of the early micrograph subsets were generated, with two copies of the best class (boxed in red) and four copies of a junk class (boxed in green) used for heterogeneous refinement with all particles from (E). (G) After heterogeneous refinement with symmetry imposed, the highest resolution class comprised of 579,111 particles was selected. Particles were re-extracted, resulting in 572,828 particles and non-uniform refinement run (H), followed by local CTF refinement to improve per-particle defocus estimation, Global CTF refinement to correct for residual per-exposure group beam tilt and magnification anisotropy, before further non-uniform refinement and referenced based motion correction. After referenced based motion correction, an experimental 2D classification (I) resulting in 371,054 particles in the cleanest classes. These particles were downsampled before local refinement (with non-uniform sampling enabled) (J) resulting in a final map reported by gold-standard Fourier shell correlation to be a resolution of 2.7 Å (K). (L) Local resolution estimation using the CryoSPARC implementation of the blocres algorithm, with resolution extending to ~2.4 Å at the most stable regions of the complex.
